# Organomagnesia: Reversibly High Carbon Dioxide Uptake by Magnesium Pyrazolates

**DOI:** 10.1002/advs.202403295

**Published:** 2024-06-21

**Authors:** Felix Kracht, Philipp Rolser, Paul Preisenberger, Cäcilia Maichle‐Mössmer, Reiner Anwander

**Affiliations:** ^1^ Institut für Anorganische Chemie Eberhard Karls Universität Tübingen Auf der Morgenstelle 18 72076 Tübingen Germany

**Keywords:** carbon dioxide, catalysis, epoxides, magnesium, pyrazolates

## Abstract

A series of new pyrazolate and mixed pyrazolate/pyrazole magnesium complexes is described and their reactivity toward carbon dioxide is examined. The dimeric complex [Mg(pz*^t^* ^Bu,^ *^t^* ^Bu^)_2_]_2_ inserts CO_2_ instantly and quantitatively forming the tetrameric complex [Mg(CO_2_·pz*^t^* ^Bu,^ *^t^* ^Bu^)_2_]_4_ and monomeric donor‐stabilized [Mg(CO_2_·pz*^t^* ^Bu,^ *^t^* ^Bu^)_2_(thf)_2_]. Complexes of the type [Mg*_x_* (pz^R,R^)_2_ *_x_* (Hpz^R,R^)*_y_*]*_n_* (R = *i*Pr, *t*Bu) engage in similar insertion reactions involving dissociation of the carbamic acid HOOCpz^R,R^. Even solid polymeric derivatives [Mg(pz^R,R^)_2_]*_n_* (R = Me, H) react instantaneously and exhaustively with CO_2_, the resulting [Mg(CO_2_·pz)_2_]*_m_* featuring a CO_2_ capacity of 35.7 wt% (8.2 mmol g^−1^). All described magnesium pyrazolates display completely reversible CO_2_ uptake in solution and in the solid state, respectively, as monitored via VT ^1^H NMR and in situ FTIR spectroscopy as well as thermogravimetric analysis. Fluorinated [Mg_2_(pz^CF3,CF3^)_4_(thf)_3_] does not yield any isolable CO_2_ insertion product but exhibits the highest activity in the catalytic transformation of epoxides and CO_2_ to cyclic carbonates.

## Graphical abstract

CO_2_ uptake of magnesium pyrazolates [Mg(pz^R,R^)_2_] to afford [Mg(CO_2_·pz^R,R^)_2_] is instant, quantitative, and reversible, both in solution and in the solid state. The CO_2_ affinity can be assessed by in situ Fourier‐transform infrared (FTIR)/nuclear magnetic resonance (NMR) spectroscopy, clearly surpassing that of corresponding trivalent and tetravalent rare‐earth‐metal derivatives, while pyrazolyl nucleophilicity drives the catalytic formation of cyclic carbonates in neat epoxide.

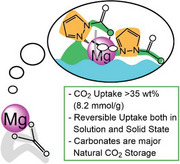

## 1 Introduction

Carbon dioxide emissions originating from fossil fuel (including energy sector and transportation; 2023: ca. 37 billion tons = 37 Gt) and as byproduct from industrial processes have been identified as the main causes of anthropogenic climate change.^[^ ^1^, ^2^, ^3^, ^4^, ^5^ ^]^ Accordingly, CO_2_ management has evolved as a top‐prioritized research field. One strategy to reduce CO_2_ emissions is to capture the post‐combustion CO_2_ before it gets released into the atmosphere, in order to either permanently sequester it or utilize it further as an effective C1 synthon in organic synthesis.^[^ ^6^, ^7^ ^]^ Crucially, any sustainable valorization of CO_2_‐loaded materials is dependent on energy‐saving, reversible processes. Since the 1930s, aqueous amine scrubbers have been used in industry, but this technology is affected by low capacities (max. < 15 wt% CO_2_), sensitivity to oxygen and high regeneration energies (high “energy penalty”).^[^ ^8^, ^9^ ^]^ Current advanced technologies for CO_2_ uptake are based on alkali/alkaline‐earth metal hydroxides, amine‐containing ionic liquids or amine‐functionalized high‐surface materials such as porous silica, zeolites, or metal‐organic frameworks (MOF),^[^ ^10^, ^11^ ^]^ with carbamate formation as the most efficient underlying principle. Striking are the properties of magnesium carboxylate‐based MOFs such as Mg(dobdc) (= Mg_2_[2,5‐dioxido‐1,4‐benzenedicarboxylate] featuring a CO_2_ capacity of up to 35 wt%,^[^ ^12^ ^]^ or the tetraamine‐appended MOF Mg(dobpdc) (= Mg_2_[4,4′‐dioxidobiphenyl‐3,3′‐dicarboxylate] which is currently the best system for cooperative CO_2_ capture from simulated flue gas.^[^ ^13^, ^14^ ^]^

**Figure 1:**
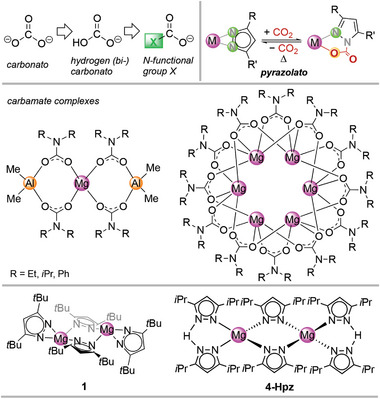
Top: design strategy of reversible molecular CO_2_ adsorbers. Middle: examples of structurally characterized magnesium carbamate complexes via CO_2_ insertion into Mg–N(amido) bonds. Bottom: examples of structurally characterized magnesium pyrazolate complexes.

Magnesium is also a crucial component in natural CO_2_ storage in the lithosphere (insoluble sedimentary carbonates: >60 000 000 Gt) and in oceans (ca. 38 000 Gt inorganic carbon: CO_2_, bicarbonate, carbonate).^[^ ^2^ ^]^ Our conceptual approach of CO_2_ capture is inspired by this natural role model and in a broader sense by the industrially applied (re)causticization of alkaline‐earth and alkali‐metal hydroxides.^[^ ^6^ ^]^ Accordingly, modification of the dianionic carbonato moiety in terms of charge and functional group (CO_3_^2−^ → HCO_3_^−^ = (HO)CO_2_^−^ → (**X**)CO_2_^−^; **X** = NR_2_ ≡ carbamato ligand) is envisaged to facilitate reversible CO_2_ uptake with minimum energy penalty (**Figure** **1**).^[^ ^15^ ^]^ In contrast to the aforementioned magnesium‐based MOFs, carbon dioxide is supposed to interact in a cooperative manner with the monoanionic nitrogen ligand **X** and the metal center.

The proof of concept was recently demonstrated for homoleptic cerium pyrazolate complexes which were found to reversibly insert CO_2_ to afford complexes [Ce^IV^(CO_2_·pz^Me,Me^)_4_] and [Ce^III^_4_(CO_2_·pz^Me,Me^)_12_] (max. 25 wt% CO_2_).^[^ ^16^ ^]^ We wondered about the applicability of this approach for magnesium as an environmentally even friendlier oxophilic earth‐abundant light metal. Pertinent magnesium‐carbamate complexes such as mixed metallic [(Me_2_Al)_2_({*µ*‐CO_2_·NR_2_}_2_)_2_Mg] (R = Et, *i*Pr) were already described 30 years ago, via CO_2_ insertion into Mg─N amido bonds (Figure 1).^[^ ^17^ ^]^ The first homoleptic magnesium carbamate complex, hexameric [Mg_6_(CO_2_·NR_2_)_12_] (R = Et, Ph) was reported in 2001.^[^ ^18^ ^]^ Since then, several more magnesium carbamate complexes appeared in the literature, however, CO_2_ insertion has proven irreversible.^[^ ^19^ ^]^ The first and only homoleptic magnesium pyrazolate complex [Mg(pz*^t^* ^Bu,^ *^t^* ^Bu^)_2_]_2_ (**1**) was reported by the group of Winter as a potential CVD precursor,^[^ ^20^ ^]^ along with the donor adducts [Mg(pz*^t^* ^Bu,^ *^t^* ^Bu^)_2_(thf)_2_]_2_ (**1**‐thf) and [Mg(pz*^t^* ^Bu,^ *^t^* ^Bu^)_2_(tmeda)] (**1**‐tmeda). Moreover, the two mixed pyrazolato/pyrazole complexes [Mg(pz*^t^* ^Bu,^ *^t^* ^Bu^)_2_(Hpz*^t^* ^Bu,^ *^t^* ^Bu^)]_2_ (**1**‐Hpz)^[^ ^21^ ^]^ and [Mg(pz*^i^* ^Pr,^ *^i^* ^Pr^)_2_(Hpz*^i^* ^Pr,^ *^i^* ^Pr^)]_2_ (**4**‐Hpz) are known.^[^ ^22^ ^]^ The present study aims to adapt the class of magnesium pyrazolates for efficient CO_2_ capture and contributes to a better understanding of the effectiveness of such pyrazolate complexes in the catalytic formation of cyclic carbonates.

## 2 Results and Discussion

### 2.1 Why Magnesium? Why Pyrazoles?

Magnesium features an earth‐abundant, non‐toxic, non‐strategic light metal and hence is prone to high CO_2_ uptake (resulting in low “mass penalty”). Pyrazoles feature the conceptually required adjacent nitrogen coordination sites, while displaying flexible coordination behavior as evidenced by many distinct coordination modes.^[^ ^23^ ^]^ As revealed by cerium pyrazolate complexes, the κ^2^(N,N’)‐coordinating pyrazolato ligand decisively promotes reversible and cooperative CO_2_ insertion by counteracting the mostly irreversible κ^2^(O,O’) coordination mode.^[^ ^24^, ^25^ ^]^ Moreover, pyrazoles are straightforwardly synthesized with easily tunable stereoelectronic properties via the 3,5‐substitution pattern. Such five‐membered heterocycles occur in natural products, albeit isolated pyrazoles cause severe toxicity for the nonsubstituted parent representative.^[^ ^26^ ^]^

### 2.2 Choice of Magnesium Pyrazolates

**Scheme 1:**
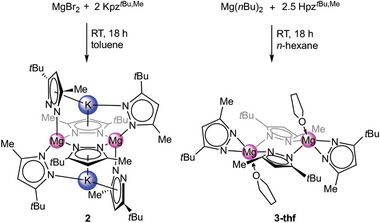
Synthesis of complexes **2** and **3**‐thf bearing the mixed 3‐*tert*‐butyl‐5‐methyl pyrazolato ligand.

This study was launched with the discrete *t*Bu‐substituted complexes [Mg(pz*^t^* ^Bu,^ *^t^* ^Bu^)_2_]_2_ (**1**) and [Mg(pz*^t^* ^Bu,^ *^t^* ^Bu^)_2_(thf)]_2_ (**1**‐thf) previously reported by Winter.^[^ ^20^ ^]^ However, due to the apparent high “mass penalty” caused by the *t*Bu moieties and the THF donor, further attempts were made to synthesize homoleptic magnesium pyrazolates with smaller substituents in the 3,5 positions, like *i*Pr/*i*Pr, mixed *t*Bu/Me, Me/Me, and H/H. To probe any marked electronic effects on CO_2_ insertion, the CF_3_/CF_3_ variant was considered as well. In our hands, salt‐metathesis reactions of magnesium bromide with 3,5‐substituted potassium pyrazolates bearing alkyl groups smaller than *t*Bu did not lead to the isolation of homoleptic magnesium pyrazolates. The mixed *t*Bu/Me pyrazolato ligand afforded ate complex [KMg(pz*^t^* ^Bu,Me^)_3_]_2_ (**2**) as the only isolable metathesis product (**Scheme** **1**). For the other alkyl‐substituted pyrazolato ligands either no reaction took place or a complex mixture of products was observed.

**Figure 2:**
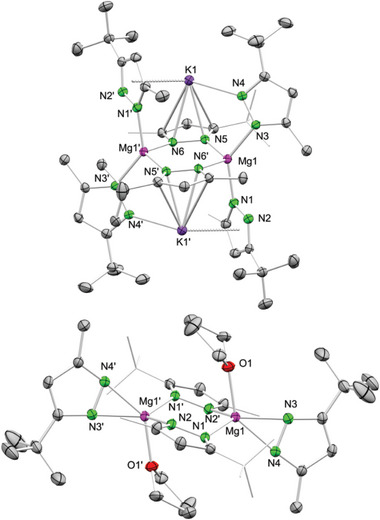
Crystal structures of [KMg(pz^*t*Bu,Me^)_3_]_2_ (**2**, top) and [Mg(pz^*t*Bu,Me^)_2_(thf)]_2_ (**3**‐thf, bottom). Ellipsoids are set at the 50% probability level. Hydrogen atoms are omitted and some Me/*t*Bu moities are displayed as wireframe for clarity. See supporting information for selected interatomic distances and angles.^[^ ^27^ ^]^

The single crystal X‐ray diffraction (SCXRD) analysis of **2** revealed a dimeric structure in which the two magnesium centers are bridged by two pyrazolato ligands in the µ−1κ(N):2κ(N’) mode forming a six‐membered ring, slightly distorted to a seat conformation (**Figure** **2**, top).^[^ ^27^ ^]^ In addition, each magnesium center is κ^1^(N)‐coordinated by two pyrazolato ligands above and beneath the metalacyclic ring implying a strongly distorted tetrahedral coordination of the magnesium atoms. The three distinct pyrazolato ligands also encapsulate the potassium cations by bridging to the magnesium centers with the approximate coordination modes µ−1κ(N):2κ(N’), µ−1κ(N):2η^5^(pz), and µ−1κ(N):2κ(N):3η^5^(pz). The equilateral triangle N5–K1–N6 is perpendicular to the pyrazolyl plane (90.05°), while the angle between the planes of the *η* ^4^‐ and *η* ^5^‐coordinating pyrazolatos is close to perpendicular (95.14°).

Despite the different coordination modes, the ^1^H NMR spectrum of **2** in [D_8_]toluene shows one signal set of the aromatic proton, the methyl and the *tert*‐butyl groups indicating a high ligand mobility in solution.

Applying a protonolysis protocol by reacting Mg(*n*Bu)_2_ with 2.5 equivalents of the corresponding pyrazole Hpz*^t^* ^Bu,Me^ in a *n*‐hexane/THF solution afforded the donor adduct [Mg(pz*^t^* ^Bu,Me^)_2_(thf)]_2_ (**3**‐thf) (Scheme 1, Figure 2, bottom).^[^ ^27^ ^]^ The crystal structure of **3**‐thf revealed a dimeric complex isostructural to Winter´s *t*Bu/*t*Bu‐congener [Mg(pz*^t^* ^Bu,^ *^t^* ^Bu^)_2_(thf)]_2_ with two µ−1κ(N):2κ(N’)‐bridging pyrazolato ligands, two terminal κ^2^(N,N’) pyrazolatos and two THF molecules.^[^ ^20^ ^]^

**Scheme 2:**
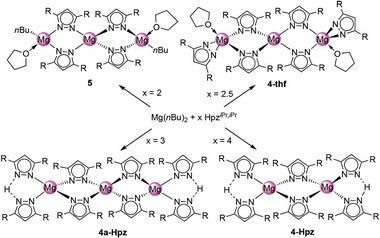
Formation of different complexes in the reaction of Mg(*n*Bu)_2_ with Hpz^*i*Pr,*i*Pr^ by using distinct ratios in THF. Complex **4**‐Hpz has been reported previously.^[^ ^22^ ^]^

The mixed pyrazolato/pyrazole complex [Mg(pz*^i^* ^Pr,^ *^i^* ^Pr^)_2_(Hpz*^i^* ^Pr,^ *^i^* ^Pr^)]_2_ (**4**‐Hpz) reported by Ruhlandt‐Senge and co‐workers was obtained via protonolysis of Mg(*n*Bu)_2_ with 4 equivalents of Hpz*^i^* ^Pr,^ *^i^* ^Pr^ in THF.^[^ ^22^ ^]^ In order to avoid two potential reactive sites for CO_2_ (vide infra) and since homoleptic [Mg(pz*^i^* ^Pr,^ *^i^* ^Pr^)_2_] has not yet been reported, we revisited the Mg(*n*Bu)_2_/Hpz*^i^* ^Pr,^ *^i^* ^Pr^ reaction. It was revealed that alkane elimination is very sensitive to the molar ratio employed. Applying a 1:2 ratio in THF did not give the anticipated homoleptic complex but incomplete protonolysis to trimetallic complex [Mg_3_(pz*^i^* ^Pr,^ *^i^* ^Pr^)_4_(*n*Bu)_2_(thf)_2_] (**5**) with two remaining *n*‐butyl moieties (**Scheme** **2**). The crystal structure revealed a central magnesium coordinated by four pyrazolato ligands, bridging to the peripheral magnesium centers in 1κ(N):2κ(N’) fashion (Figure S86, Supporting Information).^[^ ^27^ ^]^ The tetrahedral coordination of the outer magnesium atoms is completed by one *n*‐butyl moiety and one THF molecule each. Note that Mg‐alkyl moieties irreversibly insert CO_2_ to afford carboxylato ligands.^[^ ^24^, ^28^ ^]^

**Figure 3:**
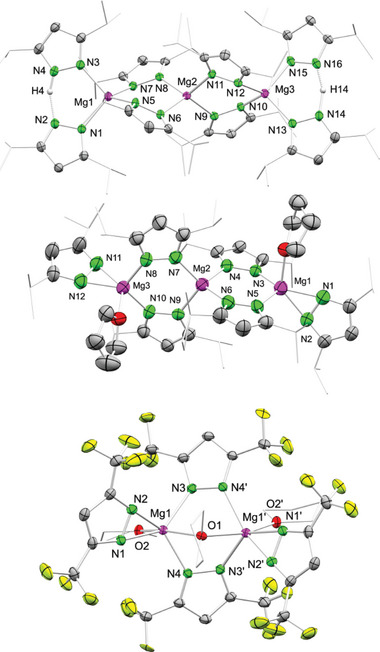
Crystal structures of [Mg_3_(pz^*i*Pr,*i*Pr^)_6_(Hpz^*i*Pr,*i*Pr^)_2_] (**4a**‐Hpz, top), [Mg_3_(pz^*i*Pr,*i*Pr^)_6_(thf)_2_] (**4**‐thf, middle) and [Mg_2_(pz^CF3,CF3^)_4_(thf)_3_] (**6**‐thf, bottom). Ellipsoids are set at the 50% probability level. Hydrogen atoms are omitted as well as the *i*Pr groups and THF ligands are displayed as wireframe for clarity. See supporting information for selected interatomic distances and angles.^[^ ^27^ ^]^

Aiming at a suitable compromise between incomplete protonolysis and coordination of excess pyrazole, the ratio of Mg(*n*Bu)_2_ to Hpz*^i^* ^Pr,^ *^i^* ^Pr^ was increased to 1:3. Now, crystallization accomplished the trimetallic mixed pyrazolato/pyrazole complex [Mg_3_(pz*^i^* ^Pr,^ *^i^* ^Pr^)_6_(Hpz*^i^* ^Pr,^ *^i^* ^Pr^)_2_] (**4a**‐Hpz, **Figure** **3**, top).^[^ ^27^ ^]^ Like in complex **5**, each magnesium center adopts a distorted tetrahedral coordination sphere. Also, the central magnesium is coordinated by four η^1^(N)‐pyrazolato ligands, bridging to the outer magnesium atoms in the same fashion as in **5**. Overall, a spiro arrangement of two six‐membered rings in half‐chair conformation about the central magnesium is observed. The outer magnesium atoms are further coordinated by pyrazolato and pyrazole ligands, which are connected via a N─H─N hydrogen bond. The pyrazolato and pyrazole ligands display a staggered arrangement when viewed along the axis of the magnesium centers.

The ^1^H NMR spectrum of **4a**‐Hpz in [D_8_]toluene indicated a mixture of different species and, hence, fragmentation in solution. Remarkably, two proton signals appeared at 19.89 and 19.51 ppm, respectively, highly shifted to lower field and with a ratio of 1:0.2 (Figure S9, Supporting Information). Additionally, three signals in the region for the proton at the pyrazole ring can be observed along with several septet and doublet signals in the expected region for *i*Pr moieties. The ^1^H NMR spectra of **4**‐Hpz and **4a**‐Hpz show the same signal pattern/fragments in [D_8_]toluene but different signal intensities, due to distinct pyrazolato/pyrazole ratios. The two strong low‐field shifted signals in **4**‐Hpz appear in the reversed ratio of 0.2:1. The intensity of all other signals of **4**‐Hpz are equally reversed, except for one signal at 5.84 ppm of the pyrazolato ring proton. The strongly low‐field shifted proton signals corroborate a low‐barrier hydrogen bond (LBHB) typical of symmetric hydrogen bonding.^[^ ^29^ ^]^

The very same fragmentation of **4**‐Hpz and **4a**‐Hpz in solution was confirmed by ^1^H DOSY (diffusion‐ordered spectroscopy) NMR experiments in [D_8_]toluene, revealing [Mg_2_(pz*^i^* ^Pr,^ *^i^* ^Pr^)_4_(Hpz*^i^* ^Pr,^ *^i^* ^Pr^)_2_] (*M*  =  958.02 g mol^−1^) and [Mg_2_(pz*^i^* ^Pr,^ *^i^* ^Pr^)_4_(Hpz*^i^* ^Pr,^ *^i^* ^Pr^)] (*M*  =  805.78 g mol^−1^) as preferred fragments (for an in‐depth discussion and other evaluation methods see the supporting information).^[^ ^30^ ^]^

Using a ratio of 1:2.5 of Mg(*n*Bu)_2_ and Hpz*^i^* ^Pr,^ *^i^* ^Pr^ in the presence of THF first led to the isolation of a white amorphous powder, which after several recrystallization steps in *n*‐pentane afforded single‐crystalline [Mg_3_(pz*^i^* ^Pr,^ *^i^* ^Pr^)_6_(thf)_2_] (**4**‐thf, Figure 3, middle).^[^ ^27^ ^]^ The white amorphous powder is most likely the homoleptic donor‐free [Mg(pz*^i^* ^Pr,^ *^i^* ^Pr^)_2_]*_n_* (**4**) forming an insoluble infinite chain structure. The crystal structure of **4**‐thf is similar to that of **4**‐Hpz except for the terminating donor ligand (THF versus Hpz). The syntheses of **3**‐thf and **4**‐thf underline that the formation of homoleptic [Mg(pz^R,R`^)_2_]*_n_* occurs preferentially when Mg(*n*Bu)_2_ and Hpz^R,R`^ are employed in a ratio of 1:2.5.

**Scheme 3:**
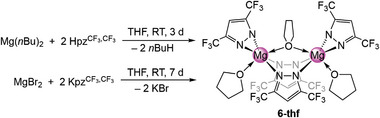
Synthesis of complex **6**‐thf according to salt‐metathesis and protonolysis protocols.

For the 3,5‐bis(trifluoromethyl)pyrazole, both the protonolysis and the salt‐metathesis route in THF yielded [Mg_2_(pz^CF3,CF3^)_4_(thf)_3_] (**6**‐thf) as a crystalline material (**Scheme** **3**). The crystal structure of **6**‐thf revealed 5‐coordinate magnesium centers in a strongly distorted trigonal bipyramidal geometry (Figure 3, bottom).^[^ ^27^ ^]^ The magnesium centers are bridged by two µ−1κ(N):2κ(N’) pyrazolato and one µ−1κ(O):2κ(O) THF ligand.^[^ ^31^ ^]^ The bridging THF distorts the six‐membered metallacycle to a twisted boat conformation. The ^1^H, ^13^C and ^19^F NMR spectra of **6**‐thf revealed high mobility of the pyrazolato ligands in solution at ambient temperature. Both ^1^ *J*_C,F_ (267.77 Hz) and ^2^ *J*_C,F_ couplings (36.58 Hz) were observed as quartets at 123.0 ppm and 142.7 ppm, respectively.^[^ ^32^ ^]^

Protonolysis of Mg(*n*Bu)_2_ with both the 3,5‐dimethylpyrazole and the unsubstituted “parent” pyrazole gave amorphous white powders, which are insoluble in common solvents. However, the ^13^C CP/MAS (magic‐angle spinning) spectra unambiguously revealed the formation of [Mg(pz^Me,Me^)_2_]*_n_* (**7**) and [Mg(pz)_2_]*_n_* (**8**). For compound **7** the carbon resonances in the 3 and 5 position appeared as one signal at 152.1 ppm, while the carbon in the 4 position and the Me moieties were detected at 107.1 ppm and as two close signals at 12.0 and 10.9 ppm, respectively. As expected, compound **8** shows only two ^13^C signals in the aromatic region at 141.6 and 103.1 ppm, respectively. Both compounds most likely form an infinite chain structure like [Zn(pz)_2_]*_n_* which was characterized by powder X‐ray diffraction.^[^ ^33^ ^]^ This is underlined by the fact, that the DRIFT spectra of **8** and [Zn(pz)_2_]*_n_* are nearly identical (Figure S79, Supporting Information).

The level of magnesium pyrazolate hydrolytic stability was probed for [Mg(pz*^t^* ^Bu,^ *^t^* ^Bu^)_2_]_2_ (**1**) and [Mg(pz)_2_]*_n_* (**8**). When exposed to air (and moisture) a solution of **1** in THF shows slow decomposition as indicated by anhould incipient precipitation (Mg(OH)_2_) and formation of the respective pyrazole. A morphology change of mostly insoluble, flaky **8**, mainly affecting the surface of the material, was clearly visible in SEM images upon exposure to ambient atmosphere (Figures  S81–S83, Supporting Information).

### 2.3 CO_2_ Insertion into Magnesium Bis(pyrazolate)s under Anhydrous Conditions

**Scheme 4:**
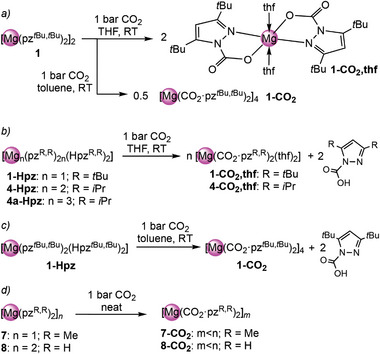
Reaction of magnesium pyrazolates with excess CO_2_.

Exposing dimeric [Mg(pz*^t^* ^Bu,^ *^t^* ^Bu^)_2_]_2_ (**1**) to 1 bar CO_2_ in THF led to the insertion of CO_2_ into the Mg─N(pyrazolato) bonds, forming the monomeric carbamate complex [Mg(CO_2_·pz*^t^* ^Bu,^ *^t^* ^Bu^)_2_(thf)_2_] (**1**‐CO_2_,thf) (**Scheme** **4**a).^[^ ^34^ ^]^ The reaction occurs instantly in a small‐scale reaction and single crystals could be obtained overnight at ambient temperature. The mass fraction of **1‐**CO_2_,thf amounts to 14.3 wt% CO_2_ or 3.3 mmol CO_2_ per gram.

**Figure 4:**
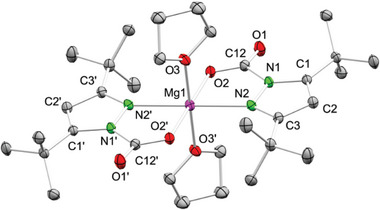
Crystal structure of [Mg(CO_2_·pz^*t*Bu,*t*Bu^)_2_(thf)_2_] (**1**‐CO_2_,thf). Ellipsoids are set at the 50% probability level. Hydrogen atoms as well as disorder in the thf ligands are omitted for clarity. See supporting information for selected interatomic distances and angles.^[^ ^27^ ^]^

Complex **1**‐CO_2_,thf displays a distorted octahedral coordination geometry, the magnesium center being coordinated by two nearly planar CO_2_·pz*^t^* ^Bu,^ *^t^* ^Bu^ carbamato moieties in the κ^2^(N,O) mode, and two apical THF donor molecules (**Figure** **4**).^[^ ^35^ ^]^ The inserted CO_2_ moiety exhibits an angle of 129.23(9)° and distinct C─O distances of 1.2165(12) and 1.2621(11) Å, which however, are less divergent from each other than in cerium complex [Ce(CO_2_·pz^Me,Me^)_4_] (1.207 Å and 1.291 Å).^[^ ^16a^ ^]^ This can be attributed to the less oxophilic character of magnesium compared to cerium.^[^ ^36^ ^]^ The pyrazole ring of the carbamato ligand loses its aromaticity compared to the pyrazolato ligand of precursor **1**, as revealed by distinct interatomic distances of both the C─C ring bonds (C2─C1: 1.3788(13)  Å; C2─C3: 1.4073(13)  Å) and the C─N ring bonds (C1─N1: 1.3800(12)  Å; C3─N2: 1.3310(12)  Å).

Carbon dioxide insertion into homoleptic pyrazolate **1** was initially observed by DRIFTS measurements showing the characteristic stretching vibration of the C─O double bond at $\tilde{\nu }$  =  1745 cm^−1^ and of the C─O single bond at $\tilde{\nu }$  =  1293 cm^−1^ (Figure S75, Supporting Information). The ^1^H NMR spectrum of **1**‐CO_2_,thf in [D_8_]THF revealed the characteristic splitting of the *t*Bu resonances into two signals at *δ*  =  1.48 ppm and *δ*  =  1.24 ppm (Figure S24, Supporting Information) due to the asymmetry caused by the inserted CO_2_. The ^13^C NMR signal of the inserted CO_2_ appeared at 149.0 ppm (Figure S25, Supporting Information) which is in the range of known complexes with CO_2_·pz^R,R^ moieties.^[^ ^16^, ^37^ ^]^

**Scheme 5:**
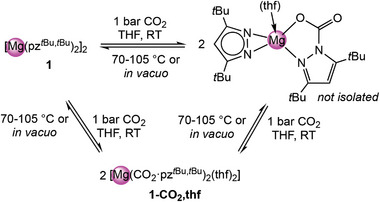
Reversibility of CO_2_ insertion into the Mg─N bond of [Mg(pz^*t*Bu,*t*Bu^)_2_]_2_ (**1**) at elaborate temperature or reduced pressure (10^‐2^ mbar).

Complex **1**‐CO_2_,thf is stable in solution at ambient temperature and as a solid at ‐40 °C. In neat form, CO_2_ slowly volatilizes at ambient temperature, as monitored by ^1^H NMR spectroscopy. Under reduced pressure (ca. 10^−2^ mbar), enhanced CO_2_ evaporation occurred, and after 6 h a mixture of **1**‐CO_2_,thf, **1**, and a proposed mono‐inserted complex was observed in the ^1^H NMR spectrum, in a ratio of 1:0.8:0.8 (Figure S26, Supporting Information). Admitting in turn 1 bar of CO_2_ led to the complete re‐formation of **1**‐CO_2_,thf. This reversible behavior was further investigated by variable‐temperature (VT) ^1^H NMR spectroscopy (Figure S27, Supporting Information). The signal of **1** emerged at 70 °C, while at 105 °C **1**‐CO_2_,thf was completely converted into **1**. Cooling to ambient temperature did not afford complete re‐insertion of CO_2_ but again a mixture of **1**‐CO_2_,thf, **1**, and the mono inserted complex in a ratio of 1:1:1. After renewed addition of 1 bar CO_2_, the mixture quantitatively reformed **1**‐CO_2_,thf, indicating that the overall CO_2_ insertion step is fully reversible (**Scheme** **5**).^[^ ^38^ ^]^

The CO_2_ de‐insertion process of **1**‐CO_2_,thf was further investigated using thermogravimetric analysis (TGA) by heating the solid from 28 °C to 450 °C and applying a heating rate of 2 K min^−1^ under constant argon flow (Figure S71, Supporting Information). The first step of 22.16 % mass loss between 78 °C and 134 °C corresponds to the release of the THF donor ligands (calcd. 23.5%). This is followed by two distinct de‐insertion steps of each CO_2_ (calcd. 7.2 % each) with mass‐loss events of 4.70 % between 134 °C and 170 °C and 6.15 % between 170 °C and 233 °C. The deviation from the calculated value can be explained by incipient gas formation before the TGA experiment and slightly overlapping release of donor THF and the first CO_2_ de‐insertion step. The two distinct de‐insertion steps observed by TGA support the proposed formation of the mono inserted complex, indicated by ^1^H NMR spectroscopy (Scheme 5).

The reaction of unsolvated **1** with CO_2_ in toluene or benzene did not afford a single‐crystalline product. However, the occurrence of a CO_2_ insertion was clearly indicated by the respective ^1^H NMR spectrum (Figure S30, Supporting Information). Five signals for the pyrazole backbone protons between 5.89 and 6.05 ppm each with an integral of 1 and nine signals for *t*Bu moieties between 0.87 and 1.78 ppm, of which eight have an integral of 9 and one has an integral of 18, are detected. This pattern indicates that **1** reacts with CO_2_ in non‐donating solvents to a putatively oligomeric species **1**‐CO_2_ (Scheme 4a). Compound **1**‐CO_2_ was further examined by ^1^H DOSY NMR spectroscopy in [D_8_]toluene (Figures S31–S33, Supporting Information). Accordingly, only one large signal was revealed with a diffusion coefficient of log*D* = −8.96 log(m^2^ s^−1^) for all the ^1^H NMR signals mentioned above. The molar mass calculation of the measured diffusion coefficient, approximated by a highly compact sphere, accounts for a molar mass of 1875 g mol^−1^. This suggests that **1**‐CO_2_ is a tetrameric oligomer/cluster containing four Mg(CO_2_·pz*^t^* ^Bu,^ *^t^* ^Bu^)_2_ units with *M* = 1883.59 g mol^−1^ (see Supporting Information for a more in‐depth discussion).^[^ ^30^ ^]^ The proposed complex [Mg(CO_2_·pz*^t^* ^Bu,^ *^t^* ^Bu^)_2_]_4_ (**1**‐CO_2_) achieves a capacity of 18.7 wt% CO_2_ or 4.3 mmol CO_2_ per gram.

Reacting **4**‐thf with 1 bar CO_2_ led to the formation of [Mg(CO_2_·pz*^i^* ^Pr,^ *^i^* ^Pr^)_2_(thf)_2_] (**4**‐CO_2_,thf) (not shown in Scheme 4). Although the ^1^H NMR spectrum of **4**‐thf is rather complicated (Figure S16, Supporting Information), due to possible fragmentation/oligomerization like in **4**‐Hpz and **4a**‐Hpz, the ^1^H NMR spectrum of **4**‐CO_2_,thf revealed complete and clean CO_2_ insertion as indicated by two distinct *i*Pr groups. The ^13^C NMR showed the corresponding *i*Pr signal patterns and a resonance at 149.6 ppm for inserted CO_2_. Unfortunately, complex **4**‐CO_2_,thf could not be obtained in single‐crystalline form. Conducting the **4**‐thf/CO_2_ reaction in toluene (not shown in Scheme 4), gave also a complex ^1^H NMR spectrum (Figures S40 and S41, Supporting Information), but resulted in a few crystals suitable for an XRD analysis. The crystal structure of LiMg_4_(CO_2_·pz*^i^* ^Pr,^ *^i^* ^Pr^)_9_, albeit of low quality (connectivity structure only) clearly revealed an exclusive carbamato environment of the metal centers (Figure S91, Supporting Information). The magnesium atoms are arranged tetrahedrally around a central lithium atom. Contamination of the reaction mixture with lithium originated from precursor Mg(*n*Bu)_2_ as revealed by ^7^Li NMR spectroscopy. We assume that crystallization of putative donor‐free [Mg_4_(CO_2_·pz*^i^* ^Pr,^ *^i^* ^Pr^)_8_] is strongly favored by the presence of the alkali metal.

Treatment of the mixed pyrazolato/pyrazole complexes **4**‐Hpz, **4a**‐Hpz, and known [Mg(pz*^t^* ^Bu,^ *^t^* ^Bu^)_2_(Hpz*^t^* ^Bu,^ *^t^* ^Bu^)]_2_ (**1**‐Hpz)^[^ ^22^ ^]^ with CO_2_ was performed in the same way as for **1** (Scheme 4b). The ^1^H NMR spectra of the **1**‐Hpz/CO_2_ reactions revealed signal sets identical to those of **1**‐CO_2_,thf ([D_8_]THF) and **1**‐CO_2_ (in [D_8_]toluene); for the reaction in THF, the formation of **1**‐CO_2_,thf could be confirmed by a unit‐cell check of the obtained crystals. In both solvents, an additional set of four new broad signals is detected: two in the range of *t*Bu moieties (*δ*  =  1.28 and 1.23 ppm), one in the aromatic range (*δ*  =  5.82 ppm) and one in the acidic proton range at *δ*  =  11.34 ppm, which does not align with free Hpz*^t^* ^Bu,^ *^t^* ^Bu^ (Figure S34, Supporting Information). Furthermore, the presence of two *t*Bu signals corroborates a reaction of CO_2_ with the donor pyrazole to yield the new carbamic acid HOOCpz*^t^* ^Bu,^ *^t^* ^Bu^. Since free pyrazoles, like aromatic amines, do not react with CO_2_ under these conditions, it can be hypothesized that the hydrogen‐bond pyrazolato/pyrazole arrangement is a prerequisite for forming the carbamic acid/ammonium carbamate.^[^ ^39^ ^]^ A similar motif was observed for compound [{Cd(*µ*‐ac)_2_(Hpz)_2_}*_n_*] (ac = acetate) in which the acetate has an H‐bond interaction with the pyrazole.^[^ ^33^ ^]^ Such two‐fold activation of CO_2_ also applies for the isopropyl‐substituted pyrazole adducts **4**‐Hpz and **4a**‐Hpz. The ^1^H NMR spectra display one signal set assignable to [Mg(CO_2_·pz*^i^* ^Pr,^ *^i^* ^Pr^)_2_(thf)_2_] (**4**‐CO_2_,thf) with the expected splitting of the *i*Pr signals. Additionally, one signal set appeared ascribed to the carbamic acid HOOCpz*^i^* ^Pr,^ *^i^* ^Pr^. Both starting materials lead to similar spectra, with varying signal intensities, due to the different pyrazolato/pyrazole ratios. As for [Mg(pz*^t^* ^Bu,^ *^t^* ^Bu^)_2_(Hpz*^t^* ^Bu,^ *^t^* ^Bu^)]_2_ (**1**‐Hpz), the CO_2_ insertion is reversible for the carbamato ligand in **4**‐CO_2_,thf, but remains irreversible for the disassociated carbamic acid, under the conditions applied (Scheme 4c).

Complex [Mg(pz*^t^* ^Bu,Me^)_2_(thf)]_2_ (**3**‐thf) featuring the mixed *t*Bu/Me 3,5‐substitution of the pyrazolato ligand clearly engaged in CO_2_ insertion. The resulting ^1^H NMR spectra, however, revealed very complicated signal pattern, which is why this complex was not pursued further. In contrast, CO_2_ insertion was not detected for fluorinated [Mg_2_(pz^CF3,CF3^)_4_(thf)_3_] (**6**‐thf), likely due to the decreased nucleophilicity of the pyrazolato nitrogen atoms (vide infra).

**Figure 5:**
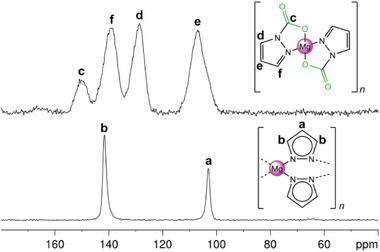
^13^C CP/MAS NMR spectra (75.47 MHz) of [Mg(pz)_2_]_*n*_ (**8**) and [Mg(CO_2_·pz)_2_]_*n*_ (**8**‐CO_2_).

Unexpectedly, the polymeric pyrazolates Mg(pz^Me,Me^)_2_]*_n_* (**7**) and [Mg(pz)_2_]*_n_* (**8**), which are insoluble in most common solvents, feature a rather remarkable CO_2_‐insertion behavior Scheme 3d). When exposing the solids in a very simple manner to an atmosphere of 1 bar CO_2_ for three hours, a mass gain of 29.4 wt% for **7** and 36.3 wt% for **8** were found, accompanied by slight morphology change and warming of the samples. This is close to the calculated exhaustive CO_2_ insertion into the Mg–N(pyrazolato) bond, accounting for 29.1 and 35.7 wt% CO_2_, respectively. The obtained materials [Mg(CO_2_·pz^Me,Me^)_2_]*_n_* (**7**‐CO_2_) and [Mg(CO_2_·pz)_2_]*_n_* (**8**‐CO_2_) were further examined by TGA (Figures S72 and S73, Supporting Information) and solid‐state NMR and FTIR spectroscopy. For **7**‐CO_2_, a CO_2_ release step of 29.5 % was observed when heating a sample from ambient temperature to 210 °C, in line with the CO_2_ mass fraction. Similarly, material **8**‐CO_2_ revealed a mass‐loss event of 34.3 %, in the range from 50 °C to 225 °C. Different from **1**‐CO_2_,thf and **8**‐CO_2_, where the CO_2_ de‐insertion only starts at elevated temperatures, the CO_2_ release for **7**‐CO_2_ already takes place at ambient temperature. The IR spectra of both complexes **7**‐CO_2_ and **8**‐CO_2_ (Figures S76 and S77, Supporting Information) show an intense and broad band between 1709 and 1744 cm^−1^ for the C─O double bond stretching vibration of the inserted CO_2_. The CO_2_ insertion/de‐insertion behavior of [Mg(pz)_2_]*_n_* (**8**) was further elucidated by conducting an in situ IR experiment. When replacing the argon atmosphere by CO_2_, immediate formation to **8**‐CO_2_ took place. When restoring the argon atmosphere and heating in 10 °C steps to 240 °C, the C─O double bond stretching vibration at 1709 cm^−1^ slowly decreased and the overall IR spectrum converted back to that of **8** (Figure S80, Supporting Information). The ^13^C CP/MAS NMR spectrum of **7**‐CO_2_ revealed distinct carbon atoms at the 3 and 5 positions (148.6 and 142.7 ppm) in comparison to **7** (152.1 ppm) The ^13^C signal of the inserted CO_2_ appeared as a shoulder to the signal at 148.6 ppm. To further clarify the signal assignment complex **7** was treated with labeled ^13^CO_2_, which resulted in two intense signals at 152.4 and 148.7 ppm. The signals of the methyl groups appeared as one signal at 12.3 ppm. Upon CO_2_ insertion, **7**‐CO_2_ is soluble in THF and the respective ^1^H NMR spectrum confirmed its formation by two signals at 2.46 and 2.12 ppm of the methyl groups in 3 and 5 position. The solution ^13^C NMR signal of the inserted CO_2_ could be resolved at 149.8 ppm. The ^13^C CP/MAS NMR spectrum of **8**‐CO_2_ shows four distinct signals in the aromatic region (**Figure** **5**). The inserted CO_2_ appeared as a signal at 150.2 ppm, and the carbon atoms in 3 and 5 position at 139.1 and 128.6 ppm, and the carbon in 4 position at 107.2 ppm. Repeated CO_2_ insertion/de‐insertion was probed for **7/7**‐CO_2_ and proven to be fully reversible by solid‐state NMR and FTIR spectroscopy (Figures S51 and S78, Supporting Information).

Comparative studies with literature known [Zn(pz)_2_]*_n_* and CO_2_ did not indicate any appreciable CO_2_ insertion.^[^ ^40^ ^]^ Although both metal centers have the same charge and similar ionic radii, the pronounced oxophilicity of magnesium^[^ ^36^ ^]^ combined with the harder Mg(II) ion determine the high CO_2_ affinity of the pyrazolate complexes compared to the zinc congeners.

Having established these solid/gas reactions for **7** and **8**, a solid sample of **1** was exposed similarly to 1 bar CO_2_. A mass gain of 18.4 % close to the calculated mass fraction of 18.7 wt% CO_2_ suggested the formation of putative **1**‐CO_2_ (vide supra). Dissolving the obtained solid in [D_8_]THF and [D_8_]toluene gave ^1^H NMR spectra identical to those of **1**‐CO_2_,thf and **1**‐CO_2_, respectively. The TGA of **1**‐CO_2_ showed a continuous mass loss with less distinct releasing steps, which would be expected for a cluster species. For further comparison, our previously reported ceric pyrazolate [Ce(Me_2_pz)_4_(thf)] did not insert the heteroallene when stored under 1 bar CO_2_ pressure for three days.^[^ ^16a^ ^]^

### 2.4 Catalytic Formation of Cyclic Carbonates from CO_2_ and Epoxides

The discrete complexes [Mg(pz*^t^* ^Bu,^ *^t^* ^Bu^)_2_]_2_ (**1**), [Mg(pz*^t^* ^Bu,Me^)_2_(thf)]_2_ (**3**‐thf), [Mg_3_(pz*^i^* ^Pr,^ *^i^* ^Pr^)_6_(Hpz*^i^* ^Pr,^ *^i^* ^Pr^)_2_] (**4a**‐Hpz), [Mg_3_(pz*^i^* ^Pr,^ *^i^* ^Pr^)_6_(thf)_2_] (**4**‐thf), and [Mg_2_(pz^CF3,CF3^)_4_(thf)_3_] (**6**‐thf) were probed as catalysts in the cycloaddition of epoxides and CO_2_ to cyclic carbonates (see Scheme S2, Supporting Information for a proposed mechanism). For better comparability, the conditions stated in **Table** **1** have been adapted to those reported previously for the rare‐earth‐metal pyrazolates.^[^ ^16a^ ^]^

**Table 1:** Formation of cyclic carbonates from epoxides and CO_2_, catalyzed by magnesium pyrazolates under study.

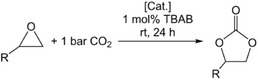				
Entry^a)^	Catalyst	R =	Conversion [%]^b)^	TON^c)^
1	**1**	Me	56	112
2^d)^	**1**	Me	85	85
3	**1**	Ph	4	8
4	**1**	*t*Bu	4	8
5	**1**	*n*Bu	7	14
6	**1**‐thf	Me	51	102
7	**3**‐thf	Me	43	86
8	**4a**‐Hpz	Me	45	90
9^e)^	**4a**‐Hpz	Me	77	51
10	**4a**‐Hpz	Ph	4	8
11	**4a**‐Hpz	*t*Bu	3	6
12	**4a**‐Hpz	*n*Bu	11	22
13	**4**‐thf	Me	42	84
14	**6**‐thf	Me	59	118
15	**6**‐thf	Ph	8	16
16	**6**‐thf	*t*Bu	4	8
17	**6**‐thf	*n*Bu	18	36
18^f)^	–	Me	3	6

^a)^ Reaction conditions if not noted otherwise: 1 bar CO_2_, 0.25 mol% catalyst or 0.167 mol% for **4a‐Hpz** and **4‐Hpz** (≡0.5 mol% Mg centers), 1 mol% TBAB at ambient temperature for 24 h in neat epoxide;

^b)^ Determined by comparison of the proton integrals in α‐position of the epoxide and the corresponding cyclic carbonate (expect for 3,3‐dimethyl‐1,2‐butene carbonate where the integral of the *t*Bu moieties was used);

^c)^ ((epoxide/Mg) · conversion)/100;

^d)^ 1 bar CO_2_, 1 mol% catalyst **1**, 1 mol% TBAB, at ambient temperature for 24 h;

^e)^ 1 bar CO_2_, 0.5 mol% catalyst **4a‐Hpz**, 1 mol% TBAB, at ambient temperature for 24 h.

^f)^ only TBAB without metal catalyst; TON refers to concentration TBAB.

Complex [Mg(pz*^t^* ^Bu,^ *^t^* ^Bu^)_2_]_2_ (**1**) gave 56 % conversion of propylene oxide and CO_2_ to the cyclic carbonate, with a TON of 112 (Table 1, entry 1). By using a twofold catalyst load of 1 mol% the conversion increased to 85 %, while the TON decreased to 85 (entry 2). This might result from catalyst aggregation in solution, by exceeding the solubility of **1** in epoxide or due to a solubility gradient shift between epoxide and cyclic carbonate. As expected, donor adduct **1**‐thf showed a slightly lower conversion of 51 % (entry 6). Similarly, for catalysts **3**‐thf, **4a**‐Hpz and **4**‐thf the conversion slightly decreased to 43 %, 45 % and 42 %, respectively (entries 7 to 8 and 13). As mentioned before, the pyrazole donors disassociate upon CO_2_ insertion as carbamic acid which can lead also to solubility shifts or blocking of the metal center. Increasing the amount of **4a**‐Hpz from 0.167 mol% (≡0.5 mol% Mg centers) to 0.5 mol% complex **4a**‐Hpz (≡1.5 mol% Mg centers) led to an enhanced conversion of 77 % but also here the TON was reduced to 51 (entry 9). Accordingly, the effects of reduced catalytic activity due to high catalyst load seem to be intrinsic.

Interestingly, fluorinated complex **6**‐thf shows the highest catalytic activity with a TON of 118 (entry 14), despite a side reaction, causing a highly viscous reaction mixture. Crucially, **6**‐thf exhibits the highest catalytic activity although it does not insert CO_2_ (vide supra). Consequently, catalytic activity and CO_2_ affinity seem to be inversely proportional. As expected, switching to the more sterically demanding epoxides with *t*Bu, *n*Bu and Ph moieties, the conversion is drastically decreased ranging from 4% to 11% (entries 3–5, 10–12 and 15–17). Again, striking is comparatively increased catalytic activity in case of **6**‐thf, giving 18% conversion of epoxyhexane in the absence of any side reaction. Complexes **7** and **8** showed also moderate conversion, however, due to high contents of insoluble oligomeric/polymeric side products, these values were not considered representative.

To determine the state of the catalyst after the catalytic reaction, each 10 mol% of **1** and TBAB were employed and the reaction mixture examined by ^1^H NMR spectroscopy (Figure S70, Supporting Information). Besides the signals for TBAB and propylene carbonate, one signal set could be assigned to complex [Mg(CO_2_·pz*^t^* ^Bu,^ *^t^* ^Bu^)_2_(thf)_2_] (**1**‐CO_2_,thf). Moreover, three additional signals in the region for pyrazolate backbones were detected, assignable neither to the carbamic acid, a mono‐inserted species nor to a hydrolysis product.

The overall catalytic activity of the magnesium pyrazolates is only moderate in comparison to the rare‐earth‐metal congeners.^[^ ^16a^ ^]^ For example, cerium pyrazolate [Ce(Me_2_pz)_4_]_2_ converted up to 98 % of propylene oxide with a TON of 196, under the same conditions. By heating the cerium reaction up to 90 °C, applying a pressure of 10 bar CO_2_ and using a catalytic load of 0.1 mol% the TON could even be increased to 600. This is clearly reflected in the higher CO_2_ release temperatures of magnesium pyrazolates (higher CO_2_ affinity), in agreement with the NMR/TGA studies (vide supra). Also, in comparison to other magnesium catalysts used in this very cycloaddition reaction, our complexes show only moderate catalytic activity.^[^ ^35^, ^41^ ^]^ Although hardly comparable due to much harsher reaction conditions, a magnesium porphyrin complex with an incorporated tetra‐*n*‐butylammonium reported by Ema et al. reaches TONs of up to 138 000 with a catalytic load of 0.0005 mol%, at 120 °C and 17 bar CO_2_.

## 3 Conclusion

The conceptional approach of carbon dioxide insertion into metal pyrazolates is applicable to the light metal magnesium. Exhaustive CO_2_ insertion into the Mg–N(pz^R,R^) bond is proven by the crystal structure of [Mg(CO_2_·pz*^t^* ^Bu,^ *^t^* ^Bu^)_2_(thf)_2_] as well as ^13^C NMR spectroscopy and thermogravimetric analysis of [Mg(CO_2_·pz^R,R^)_2_]*_n_* (R = H, Me, *i*Pr). Carbon dioxide uptake at ambient temperature is instant and reversible with de‐insertion temperatures <250 °C. Remarkably, CO_2_ insertion also occurs quantitatively using the magnesium pyrazolates in solid form (without solvent!). Unsubstituted [Mg(CO_2_·pz)_2_]*_n_* accomplishes a maximum capacity of 35.7 wt% CO_2_ (8.2 mmol g^−1^) leveling that of the most efficient metalorganic frameworks (MOFs). Depending on the pyrazolate substituents R, the homoleptic magnesium complexes are easily obtained via salt metathesis or protonolysis applying MgBr_2_ and Mg(*n*Bu)_2_, respectively. For the protonolysis protocol, stoichiometric control and hence pyrazole coordination are critical since the CO_2_ uptake is affected by additional formation of the respective carbamic acids HOOCpz^R,R^. The salt‐metathesis protocol is prone to ate complex formation as evidenced for [KMg(pz*^t^* ^Bu,Me^)_3_]_2_. The magnesium pyrazolates display only moderate catalytic activity in the reaction of CO_2_ and epoxides to cyclic carbonates. Crucially, catalytic activity and CO_2_ affinity seem to be inversely proportional, as revealed by the fluorinated complex [Mg_2_(pz^CF3,CF3^)_4_(thf)_3_], exhibiting the highest catalytic activity while lacking any traceable CO_2_ insertion, under the applied conditions. Overall, the catalytic activity is lower than that of the cerium congeners attributable to the higher CO_2_ release temperatures of the magnesium complexes. Due to significant hydrolytic instability such magnesium pyrazolate complexes are not meant for any industrial large scale carbon dioxide removal technology but might stimulate new inorganic material design.

## Conflict of Interest

The authors declare no conflict of interest.

## Supporting information

Supporting Information

## Data Availability

The data that support the findings of this study are available in the supplementary material of this article.

## References

[1] a) S. Dhakal , J. C. Minx , F. L. Toth , A. Abdel‐Aziz , M. J. Figueroa Meza , K. Hubacek , I. G. C. Jonckheere , Y.‐G. Kim , G. F. Nemet , S. Pachauri , X. C. Tan , T. Wiedmann , Emissions Trends and Drivers, IPCC, 2022: *Climate Change 2022: Mitigation Of Climate Change. Contribution Of Working Group III to the Sixth Assessment Report of the Intergovernmental Panel on Climate Change* , (Eds: P. R. Shukla , J. Skea , R. Slade , A. Al Khourdajie , R. van Diemen , D. McCollum , M. Pathak , S. Some , P. Vyas , R. Fradera , M. Belkacemi , A. Hasija , G. Lisboa , S. Luz , J. Malley , Cambridge University Press, Cambridge, UK and New York, NY, USA 2022;

[2] P. Falkowski , R. J. Scholes , E. Boyle , J. Canadell , D. Canfield , J. Elser , N. Gruber , K. Hibbard , P. Högberg , S. Linder , F. T. Mackenzie , B. Moore III , T. Pedersen , Y. Rosenthal , S. Seitzinger , V. Smetacek , W. Steffen , Science 2000, 290, 291. 11030643 10.1126/science.290.5490.291

[3] S. Solomon , G.‐K. Plattner , R. Knutti , P. Friedlingstein , Proc. Natl. Acad. Sci. USA 2009, 106, 1704. 19179281 10.1073/pnas.0812721106 PMC2632717

[4] R. S. Haszeldine , Science 2009, 325, 1647. 19779187 10.1126/science.1172246

[5] D. W. Keith , Science 2009, 325, 1654. 19779189 10.1126/science.1175680

[6] a) S. Chu , Science 2009, 325, 1599 ; 19779157 10.1126/science.1181637

[7] a) T. Sakakura , J.‐C. Choi , H. Yasuda , Chem. Rev. 2007, 107, 2365; 17564481 10.1021/cr068357u

[8] a) G. T. Rochelle , Science 2009, 325, 1652 ; 19779188 10.1126/science.1176731

[9] H. Chen , H. Dong , Z. Shi , A. K. SenGupta , Sci. Adv. 2023, 9, eadg1956. 36888712 10.1126/sciadv.adg1956 PMC9995034

[10] a) P. D. Jadhav , R. V. Chatti , R. B. Biniwale , N. K. Labhsetwar , S. Devotta , S. S. Rayalu , Energy Fuels 2007, 21, 3555;

[11] a) J. A. Mason , K. Sumida , Z. R. Herm , R. Krishna , J. R. Long , Energy Environ. Sci. 2011, 4, 3030;

[12] S. R. Caskey , A. G. Wong‐Foy , A. J. Matzger , J. Am. Chem. Soc. 2008, 130, 10870. 18661979 10.1021/ja8036096

[13] a) T. M. McDonald , J. A. Mason , X. Kong , E. D. Bloch , D. Gygi , A. Dani , V. Crocellà , F. Giordanino , S. O. Odoh , W. S. Drisdell , B. Vlaisavljevich , A. L. Dzubak , R. Poloni , S. K. Schnell , N. Planas , K. Lee , T. Pascal , L. F. Wan , D. Prendergast , J. B. Neaton , B. Smit , J. B. Kortright , L. Gagliardi , S. Bordiga , J. A. Reimer , J. R. Long , Nature 2015, 519, 303; 25762144 10.1038/nature14327

[14] K. Kadota , Y. Hong , Y. Nishiyama , E. Sivaniah , D. Packwood , S. Horike , J. Am. Chem. Soc. 2021, 143, 16750. 34605645 10.1021/jacs.1c08227

[15] It is worth mentioning that naturally occurring simple binary carbonates limit themselves to alkali and alkaline‐earth metals (except Be), and the divalent *d*‐metals Mn, Fe, Co, Ni, Zn, Cd; furthermore, NaHCO_3_ is the only proven natural bicarbonate. CO_2_ capture from air with caustic solutions involves a calcination process regenerating CaO from CaCO_3_ at temperatures above 700 °C.

[16] a) U. Bayer , D. Werner , C. Maichle‐Mössmer , R. Anwander , Angew. Chem., Int. Ed. 2020, 59, 5830; 10.1002/anie.201916483 PMC7155069 31916355

[17] C.‐C. Chang , B. Srinivas , W. Mung‐Liang , C. Wen‐Ho , M. Y. Chiang , H. Chung‐Sheng , Organometallics 1995, 14, 5150.

[18] K.‐C. Yang , C.‐C. Chang , C.‐S. Yeh , G.‐H. Lee , S.‐M. Peng , Organometallics 2001, 20, 126.

[19] a) M. T. Caudle , J. W. Kampf , Inorg. Chem. 1999, 38, 5474; 11671272 10.1021/ic990765b

[20] D. Pfeiffer , M. J. Heeg , C. H. Winter , Angew. Chem., Int. Ed. 1998, 37, 2517; 10.1002/(SICI)1521-3773(19981002)37:18<2517::AID-ANIE2517>3.0.CO;2-1 29711341

[21] N. C. Mösch‐Zanetti , M. Ferbinteanu , J. Magull , Eur. J. Inorg. Chem. 2002, 2002, 950.

[22] J. Hitzbleck , G. B. Deacon , K. Ruhlandt‐Senge , Eur. J. Inorg. Chem. 2007, 2007, 592.

[23] a) M. A. Halcrow , Dalton Trans. 2009, 38, 2059; 10.1039/b815577a 19274281

[24] a) R. Ayyappan , I. Abdalghani , R. C. Da Costa , G. R. Owen , Dalton Trans. 2022, 51, 11582; 35839074 10.1039/d2dt01609e

[25] G. Bresciani , L. Biancalana , G. Pampaloni , F. Marchetti , Molecules 2020, 25, 3603. 32784784 10.3390/molecules25163603 PMC7465543

[26] a) G. Magnusson , J.‐A. Nyberg , N.‐O. Bodin , E. Hansson , Experientia 1972, 28, 1198; 5087038 10.1007/BF01946169

[27] Deposition numbers 2342200 (for **2**), 2342196 (for **3‐thf**), 2342195 (for **5**), 2342199 (for **4a‐Hpz**), 2342201 (for **4‐thf**), 2342198 (for **6‐thf**), 2342197 (for **1‐CO_2_,thf**) contain the supplementary crystallographic data for this paper. These data are provided free of charge by the Cambridge Crystallographic Data Center, https://www.ccdc.cam.ac.uk/structures/ (accessed: May 2024).

[28] V. S. Pfennig , R. C. Villella , J. Nikodemus , C. Bolm , Angew. Chem., Int. Ed. 2022, 61, e202116514; 10.1002/anie.202116514 PMC9306648 34942056

[29] a) G. Gunnarsson , H. Wennerström , W. Egan , S. Forsén , Chem. Phys. Lett. 1976, 38, 96;

[30] R. Neufeld , D. Stalke , Chem. Sci. 2015, 6, 3354. 29142693 10.1039/c5sc00670h PMC5656982

[31] a) C. J. Snyder , M. J. Heeg , C. H. Winter , Inorg. Chem. 2011, 50, 9210; 21877698 10.1021/ic201541c

[32] A. V. Afonin , I. A. Ushakov , D. V. Pavlov , O. V. Petrova , L. N. Sobenina , A. I. Mikhaleva , B. A. Trofimov , J. Fluorine Chem. 2013, 145, 51.

[33] N. Masciocchi , S. Galli , E. Alberti , A. Sironi , C. Di Nicola , C. Pettinari , L. Pandolfo , Inorg. Chem. 2006, 45, 9064. 17054367 10.1021/ic061349b

[34] D. Belli DellÁmico , F. Calderazzo , L. Labella , F. Marchetti , G. Pampaloni , Chem. Rev. 2003, 103, 3857. 14531715 10.1021/cr940266m

[35] a) B. Raghavendra , K. Bakthavachalam , B. Ramakrishna , N. D. Reddy , Organometallics 2017, 36, 4005;

[36] K. P. Kepp , Inorg. Chem. 2016, 55, 9461. 27580183 10.1021/acs.inorgchem.6b01702

[37] Y.‐T. Tseng , W.‐M. Ching , W.‐F. Liaw , T.‐T. Lu , Angew. Chem., Int. Ed. 2020, 59, 11819; 10.1002/anie.202002977 32281729

[38] D. Jin , X. Sun , A. Hinz , P. W. Roesky , CCS Chem. 2023, 5, 1277.

[39] M. Aresta , D. Ballivet‐Tkatchenko , D. Belli DellÁmico , M. C. Bonnet , D. Boschi , F. Calderazzo , R. Faure , L. Labella , F. Marchetti , Chem. Commun. 2000, 1099.

[40] A. Cingolani , S. Galli , N. Masciocchi , L. Pandolfo , C. Pettinari , A. Sironi , Dalton Trans. 2006, 35, 2479. 10.1039/b515630k 16705348

[41] a) T. Ema , Y. Miyazaki , S. Koyama , Y. Yano , T. Sakai , Chem. Commun. 2012, 48, 4489; 10.1039/c2cc30591g 22460201

