# Supplementary material for: Organomagnesia: Reversibly High Carbon Dioxide Uptake by Magnesium Pyrazolates
Source: Adv Sci (Weinh). 2024 Jun 21;11(32):2403295. doi: 10.1002/advs.202403295 (PMC11348227; doi:10.1002/advs.202403295)
Supplement: Supplementary file 1 — Supporting Information [file ADVS-11-2403295-s001.pdf]

ADVANCED SCIENCE
Open Access

Supporting Information

for *Adv. Sci.*, DOI 10.1002/advs.202403295

Organomagnesia: Reversibly High Carbon Dioxide Uptake by Magnesium Pyrazolates

*Felix Kracht, Philipp Rolser, Paul Preisenberger, Cäcilia Maichle-Mössmer and Reiner Anwander**

Supporting Information

Table of Contents

Experimental Section	S3
NMR Spectra of Magnesium Pyrazolates and CO_2-Insertion Products	S7
NMR Spectra Catalysis	S36
Proposed Mechanism for the Catalytic Formation of Propylene Carbonate	S46
TGA Diagrams	S47
IR Spectra	S49
SEM	S53
Crystallographic Data	S56
References	S63

Experimental Section.

General Considerations. All manipulations were performed under rigorous exclusion of air and moisture under argon atmosphere (< 0.1 ppm O_2, < 0.1 ppm of H_2O) in a MB200B glovebox (MBraun) or according to standard Schlenk techniques and in oven-dried glassware. Solvents (THF, *n*-pentane, *n*-hexane, Et_2O and toluene) were purified by using SPS Grubbs-type columns (MBraun SPS-800, solvent purification system) and stored inside a glovebox. THF was dried further over molecular sieves. [D_6]benzene, [D_8]toluene and [D_8]THF were obtained from Sigma Aldrich and dried over Na/K alloy and filtered prior to use. 3,5-Bis(trifluoromethyl)pyrazole (99%), 3,5-dimethylpyrazole (99%), di-*n*-butylmagnesium (1 M in Et_2O/*n*-hexane), epoxystyrene (>97%, degassed prior to use) and magnesium bromide (98%) were purchased from Sigma Aldrich and used as received. 3,5-Di-iso-propylpyrazole (98%) was purchased from TCI and used as received. 1,2-Epoxyhexane (97%) was purchased from Thermo Fischer and degassed prior to use. Propylene oxide and pyrazole (98%) were purchased from Acros Organics and the former was degassed prior to use. 2-*tert*-Butyloxirane was purchased from abcr and degassed prior to use. 3-Methyl-5-*tert*-butylpyrazole and 3,5-di-*tert*-butylpyrazole were synthesized according to procedures known in the literature.[1] Argon and CO_2 (99.95%) were supplied by Westfalen AG. ^{1}H and ^{13}C NMR spectra were recorded on a Bruker AVII+400 spectrometer (^{1}H: 400.13 MHz; ^{13}C: 100.61 MHz) at 299 K. The chemical shifts listed in the experimental section are referenced to solvent residual resonances in parts per million in relation to tetramethylsilane. The variable temperature ^{1}H NMR spectra and some other spectra were recorded in a J. Young valve NMR tube on a Bruker AVII+500 spectrometer (^{1}H: 500.13 MHz; ^{13}C: 125.76 MHz). The ^{1}H DOSY NMR spectra were recorded in a J. Young valve NMR tube on a Bruker AVIII HDX 700 spectrometer (^{1}H: 700.29 MHz). IR spectra were recorded on a NICOLET 6700 FTIR spectrometer (Thermo Fisher Scientific). The samples were mixed with KBr powder and measured in a DRIFTS cell with KBr windows. In situ IR spectra were recorded on a Bruker Invenio R spectrometer equipped with a praying mantis unit. Data were converted by using the Kubelka-Munk refinement. Elementary analyses were performed on an Elementar vario MICRO cube. SEM images were obtained on a Hitachi SU8030. Single crystals were grown from saturated solutions of *n*-hexane or THF by standard techniques. Suitable single crystals for X-ray structure analysis were selected in a glovebox and coated with Parabar 10312 (Hampton research). Crystallographic data were measured on a Bruker APEX II DUO instrument equipped with IµS micro focus sealed tube and QUAZAR optics for MoKα radiation (λ = 0.71073 Å) and CuK_α radiation (λ = 1.54184 Å). Compounds **2** and **4a-Hpz** were crystallized at –40 °C from a solution of *n*-hexane, **3-thf** at ambient temperature. Crystals of **4-thf** could be obtained from *n*-pentane at –40 °C, and for **5** a mixture of chilled Et_2O/*n*-hexane (–40 °C) was used. Crystals of **1-CO₂,thf** were harvested from a THF solution at ambient temperature and those of **6-thf** at –40 °C. The crystallization of **$LiMg_4(CO_2Pz^{iPr,iPr})_9$** became feasible due to a slight contamination of the magnesium precursor with lithium, after many unsuccessful crystallization attempts. A few crystals were obtained in a J. Young tube at ambient temperature from a toluene solution. The crystal quality was low and only intensities up to 1 Å could be found. Although the structure could be solved, the calculated data only represent a connectivity structure, ruling out any detailed interpretation of metrical parameters. The data collection strategy was determined using COSMO[2] employing ω- and φ scans. Raw data were processed using APEX[3] and SAINT,[4] corrections for absorption effects were applied using SADABS.[5] The structure was solved by direct methods and refined against all data by full-matrix least-squares methods on F^2 using ShelXTL[6] and ShelXle.[7] Disorder models are calculated using DSR[8], a program for refining disordered structures in SHELXL. All graphics were produced employing Mercury.[9]

[Mg(pz^{tBu,tBu})$_2$(thf)]$_2$ (1-thf). Hpz^{tBu,tBu} (200.0 mg, 1.109 mmol) was dissolved in 10 mL THF and a solution of Mg(*n*Bu)$_2$ (0.56 mL, 0.56 mmol, 1 M in Et_2O/*n*-hexane) was added dropwise. After stirring for 2 h, the solution was concentrated under reduced pressure and stored at ambient temperature. Overnight colorless crystals were obtained suitable for an X-ray structure analysis. Drying the crystals under reduced pressure gave **1-thf** (210.0 mg, 461.6 µmol, 83%). Analytical data are consistent with the literature.[10]

[KMg(pz^{tBu,Me})$_3$]$_2$ (2). Magnesium bromide (121.7 mg, 661 µmol) and Kpz^{tBu,Me} (233.1 mg, 1.322 mmol) were dissolved in toluene and stirred for 16 h. After the solvent was removed under reduced pressure the residue was extracted three times with *n*-hexane. The solution was concentrated and stored at –40 °C. Overnight **2** (71.3 mg, 75 µmol, 23%) could be obtained as colorless crystals. ^{1}H NMR (400.1 MHz, [D_8]THF, 26 °C): δ = 5.87 (s 6H, 4-H(pz)), 2.14 (s, 18H, C*H*$_3$), 1.17 (s, 54H, C(C*H*$_3$)$_3$) ppm. ^{13}C{H} NMR (100.6 MHz, [D_8]THF, 26 °C): δ = 163.3 (3-*C*(pz)), 148.1 (5-*C*(pz)), 100.5 (4-*C*(pz)), 32.3 (*C*(CH$_3$)$_3$), 31.7 (C(*C*H$_3$)$_3$), 13.2 (*C*H$_3$) ppm. IR (Nujol/CsI): $\tilde{\nu}_{max}$ = 2953 (Nujol;

CH), 2923 (Nujol; CH), 2854 (Nujol; CH), 1515 (vs), 1497 (s), 1462 (Nujol), 1410 (vs), 1377 (Nujol), 1363 (vs), 1312 (vs), 1232 (s), 1206 (s), 1092 (m), 1072 (s), 1060 (s), 1043 (w), 1020 (s), 996 (m), 987 (m), 972 (w), 933 (vw), 923 (vw), 893 (vw), 816 (m), 772 (vs), 728 (m), 694 (m), 570 (w), 503 (w), 492 (w), 467 (w), 436 (s) cm^{-1}. Elemental analysis calcd. (%) for $C_{48}H_{78}K_2Mg_2N_{12}$ (950.04 g mol^{-1}): C 60.68, H 8.28, N 17.69; found: C 60.56, H 8.50, N 17.49.

$[Mg(pz^{tBu,Me})_2(thf)]_2$ (3-thf). $Hpz^{tBu,Me}$ (200.0 mg, 1.447 mmol) was dissolved in 10 mL THF and a solution of $Mg(nBu)_2$ (0.58 mL, 0.58 mmol, 1 M in Et_2O/*n*-hexane) was added dropwise. After stirring overnight, the solution was concentrated under reduced pressure and stored at ambient temperature. After 7 days colorless crystals were obtained suitable for an X-ray structure analysis. The supernatant slurry solution was separated and the crystals covered with a sticky oil were washed several times with *n*-pentane. Drying the crystals under reduced pressure gave **3-thf** (180.4 mg, 243 μmol, 67%). ^1H NMR (500.13 MHz, [D8]THF, 26 °C): δ = 5.78 (s, 4H, 4-*H*(pz)), 3.61 (m, 8 H, 1/4-CH_2 (thf)), 2.08 (s, 12 H, CH_3), 1.77 (m, 8 H, 2/3-CH_2 (thf)), 1.15 (s, 36 H, $C(CH_3)_3$) ppm. $^{13}C\{H\}$ NMR (125.76 MHz, [D8]THF, 26 °C): δ = 163.0 (br, 3/5-*C*(pz)), 149.6 (br, 3/5-*C*(pz)), 102.1 (4-*C* (pz)), 68.0 (1/4-*C* (thf)), 32.2 ($C(CH_3)_3$), 31.4 ($C(CH_3)_3$), 26.2 (2/3-*C* (thf)), 13.0 (CH_3) ppm. IR (Nujol/CsI): $\tilde{\nu}_{max}$ = 2950 (Nujol, CH), 2928 (Nujol, CH), 2860 (Nujol, CH), 1524 (vs), 1516 (vs), 1463 (Nujol), 1416 (s), 1377 (Nujol), 1363 (vs), 1313 (s), 1235 (s), 1207 (m), 1078 (w), 1060 (m), 1030 (s), 1020 (s), 1005 (w), 983 (w), 912 (w), 892 (w), 833 (vw), 784 (s), 735 (vw), 726 (w), 697 (w), 676 (vw), 570 (vw), 506 (m), 476 (vw), 412 (vs) cm^{-1}. Elemental analysis calcd. (%) for $C_{40}H_{68}Mg_2N_8O_2$ (741.65 g mol^{-1}): C 64.78, H 9.24, N 15.11; found: C 64.27, H 9.28, N 15.09.

$[Mg_3(pz^{iPr,iPr})_6(Hpz^{iPr,iPr})_2]$ (4a-Hpz). $Hpz^{iPr,iPr}$ (600.0 mg, 3941 μmol) was dissolved in 10 mL THF and a solution of $Mg(nBu)_2$ (1.31 mL, 1.31 mmol, 1 M in Et_2O/*n*-hexane) was added dropwise. After stirring overnight, the solvent was removed under reduced pressure. After several recrystallization steps of dissolving the residue in *n*-hexane and storing it afterwards at –40 °C, **4a-Hpz** (381.0 mg, 296.5 μmol, 68%) could be obtained as colorless crystals suitable for an X-ray structure analysis. DRIFTS: $\tilde{\nu}_{max}$ = 3357 (vw), 2962 (vs), 2928 (s), 2871 (m), 2572 (vw), 1559 (vw), 1523 (m), 1506 (m), 1458 (m), 1421 (w), 1379 (w), 1363 (m), 1304 (w), 1273 (w), 1178 (w), 1141 (w), 1095 (w), 1042 (m), 1021 (w), 923 (vw), 878 (vw), 786 (m), 726 (w), 591 (w), 528 (w), 503 (w), 472 (w), 419 (w) cm^{-1}. Elemental analysis calcd. (%) for $C_{72}H_{122}Mg_3N_{16}$ (1284.80 g mol^{-1}): C 67.31, H 9.57, N 17.44; found: C 67.10, H 9.52, N 17.54.

$[Mg_3(pz^{iPr,iPr})_6(thf)_2]$ (4-thf). $Hpz^{iPr,iPr}$ (200.0 mg, 1313 μmol) was dissolved in 10 mL THF and a solution of $Mg(nBu)_2$ (0.53 mL, 0.53 mmol, 1 M in Et_2O/*n*-hexane) was added dropwise. After stirring overnight, the solvent was removed under reduced pressure. After several recrystallization steps of dissolving the residue in *n*-pentane and storing it at –40 °C, **4-thf** (101.4 mg, 90.1 μmol, 51%) could be obtained as colorless crystals suitable for an X-ray structure analysis. DRIFTS: $\tilde{\nu}_{max}$ = 3115 (vw), 2961 (vs), 2930 (vs), 2875 (s), 1526 (vs), 1508 (s), 1481 (m), 1459 (s), 1421 (w), 1380 (s), 1363 (s), 1300 (s), 1177 (m), 1141 (m), 1104 (w), 1066 (m), 1040 (vs), 1015 (s), 996 (w), 921 (w), 886 (w), 786 (s), 726 (w), 701 (vw), 591 (vw), 526 (m), 475 (m) cm^{-1}. Elemental analysis calcd. (%) for $C_{62}H_{106}Mg_3N_{12}O_2$ (1124.53 g mol^{-1}): C 66.22, H 9.50, N 14.95; found: C 66.13, H 9.38, N 15.45.

$[Mg_3(pz^{iPr,iPr})_4(nBu)_2(thf)_2]$ (5). $Hpz^{iPr,iPr}$ (200.0 mg, 1313.7 μmol) was dissolved in 10 mL THF and a solution of $Mg(nBu)_2$ (0.66 mL, 0.66 mmol, 1 M in Et_2O/*n*-hexane) was added dropwise. After stirring overnight, the solvent was reduced and stored at –40 °C. Overnight, single crystals were grown suitable for an X-ray structure analysis. After separation of the supernatant solution and drying of the crystals under reduced pressure, **5** could be obtained as colorless powder (86.5 mg, 92.3 μmol, 42%). ^1H NMR (500.13 MHz, [D8]THF, 26 °C): δ = 5.82 (s, 4 H, 4-*H*(pz)), 3.61 (m, 1/4-CH_2 (thf)), 3.15 (broad s, 4H, $CH(CH_3)_2$), 2.71 (broad s, 4H, $CH(CH_3)_2$), 1.77 (m, 2/3-CH_2 (thf)), 1.51 (m, 4 H, $MgCH_2CH_2$), 1.39–1.06 (broad s, $CH(CH_3)_2$), 1.20 (m, CH_2CH_3), 0.99–0.62 (broad s, $CH(CH_3)_2$), 0.81 (t, CH_2CH_3), –0.39 (m, 4H, $MgCH_2$) ppm. $^{13}C\{H\}$ NMR (125.76 MHz, [D8]THF, 26 °C): δ = 163.5 (3/5-*C*(pz)), 95.6 (4-*C*(pz)), 67.7 (1/4-*C*(thf)), 33.6 ($MgCH_2CH_2$), 32.5 (CH_2CH_3), 28.8 ($CH(CH_3)_2$), 25.6 (2/3-*C*(thf)), 24.5 ($CH(CH_3)_2$), 14.2 (CH_2CH_3), 9.1 ($MgCH_2$) ppm. IR (Nujol): $\tilde{\nu}_{max}$ = 2959 (Nujol; CH), 2923 (Nujol; CH), 2856 (Nujol; CH), 1524 (m), 1504 (m), 1481 (vs), 1460 (Nujol), 1424 (m), 1377 (Nujol), 1366 (vs), 1300 (m), 1269 (vw), 1177 (w), 1141 (vw), 1105 (s), 1061 (w), 1041 (s), 1017 (m), 990 (vw), 951 (vw), 919 (w), 886 (m), 831 (vw), 784 (vs), 725 (m), 702 (vw), 681 (vw), 590 (vw), 544 (w), 527 (m), 511 (m), 477 (m) cm^{-1}. Elemental analysis calcd. (%) for $C_{52}H_{94}Mg_3N_8O_2$ (936.29 g mol^{-1}): C 66.91, H 9.87, N 12.34; found: C 66.64, H 9.96, N 12.18.

$[Mg_2(pz^{CF_3,CF_3})_4(thf)_3]$ (6-thf). Route A: Hpz^{CF_3,CF_3} (200.0 mg, 980 μmol) was dissolved in 10 mL THF and a solution of $Mg(nBu)_2$ (0.49 mL, 0.49 mmol, 1 M in Et_2O/*n*-hexane) was added dropwise. After stirring for 3 d, the solvent

was reduced and stored at –40 °C. Overnight, single crystals were grown suitable for an X-ray structure analysis. After separation of the supernatant solution and drying of the crystals under reduced pressure, **6-thf** could be obtained as dark red powder (250.0 mg, 464 μmol, 95%). Route B: Kpz^{CF_3,CF_3} (179.4 mg, 980 μmol) and $MgBr_2$ (68.2 mg, 370 μmol) were dissolved in 10 mL THF and stirred for 7 d. After the solvent was removed under reduced pressure the residue was extracted three times with *n*-hexane. The solution was concentrated and stored at –40 °C. Overnight **6-thf** (55.9 mg, 104 μmol, 28%) could be obtained as dark red crystals. ^{1}H NMR (400.1 MHz, [D$_8$]THF, 26 °C): 6.84 (s, 4 H, 4-*H*(pz)), 3.62 (m, 12 H, 1/4-CH$_2$(thf)), 1.78 (m, 12 H, 2/3-CH$_2$(thf)) ppm. ^{13}C{H} NMR (100.6 MHz, [D$_8$]THF, 26 °C): 142.7 (q, *C*(CF$_3$), $^{2}J_{C,F}$=36.58 Hz), 123.0 (q, *C*F$_3$, $^{1}J_{C,F}$=267.77 Hz), 103.3 (4-*C*(pz)), 68.0 (1,4-*C*(thf)), 26.2 (2,3-*C*(thf)) ppm. ^{19}F NMR (282.4 MHz, [D$_8$]THF, 26 °C): –60.9 (C*F*$_3$) ppm. IR (Nujol): $\tilde{\nu}_{max}$ = 3154 (vw), 2956 (Nujol, CH), 2924 (Nujol, CH), 2854 (Nujol, CH), 1651 (vw), 1637 (vw), 1540 (m), 1501 (w), 1462 (Nujol), 1456 (s), 1375 (Nujol), 1346 (s), 1261 (vs), 1223 (s), 1138 (vs), 1124 (vs), 1084 (s), 1022 (s), 999 (s), 963 (w), 919 (w), 876 (s), 844 (w), 819 (m), 755 (m), 737 (w), 718 (w), 684 (vw), 654 (vw), 600 (vw), 576 (w), 545 (vw), 468 (w), 425 (m) cm^{-1}. Elemental analysis calcd. (%) for $C_{32}H_{28}F_{24}Mg_2N_8O_3$ (1077.20 g mol^{-1}): C 35.68, H 2.62, N 10.40; found: C 35.89, H 2.55, N 10.64.

[Mg(pz^{CF_3,CF_3})$_2$]$_n$ (6). Kpz^{CF_3,CF_3} (220.0 mg, 909 μmol) and $MgBr_2$ (83.6 mg, 454 μmol) were dissolved in 10 mL toluene and stirred for 5 d. After the solvent was removed under reduced pressure the residue was extracted three times with *n*-hexane. The solvent was removed under reduced pressure affording **6** as dark red powder (101.6 mg, 236 μmol, 28%). ^{1}H NMR (300.1 MHz, [D$_8$]THF, 26 °C): 6.50 (s, 4 H, 4-*H*(pz)) ppm. ^{19}F NMR (282.4 MHz, [D$_8$]THF, 26 °C): –59.8 (C*F*$_3$) ppm. IR (Nujol): $\tilde{\nu}_{max}$ = 2953 (Nujol, CH), 2923 (Nujol, CH), 2854 (Nujol CH), 1547 (w), 1510 (vw), 1461 (Nujol), 1377 (Nujol), 1369 (vs), 1269 (m), 1228 (w), 1144 (s), 1113 (s), 1020 (vs), 989 (m), 886 (w), 822 (m), 759 (m), 735 (m), 722 (m), 608 (vw), 580 (w), 544 (w), 500 (w), 476 (w), 450 (m) cm^{-1}.

[Mg(pz^{Me,Me})$_2$]$_n$ (7). Hpz^{Me,Me} (500.0 mg, 5.201 mmol) was dissolved in 10 mL of toluene and a solution of Mg(*n*Bu)$_2$ (2.60 mL, 2.60 mmol, 1 M in Et$_2$O/*n*-hexane) was added dropwise. A white precipitate formed and the reaction mixture was stirred for 3 h. After the solvent was removed under reduced pressure, the white powder was washed three times with *n*-pentane. The white powder was then dryed further under high vacuum for 6 h to remove excess solvent, which gave **7** (536.6 mg, 2.505 mmol, 96%) as a white powder. ^{13}C CP/MAS spectrum (75.47 MHz, MAS at 8 kHz): 152.1 (3/5-*C*(pz)), 107.1 (4-*C*(pz)), 12.0 (*C*H$_3$), 10.9 (*C*H$_3$) ppm. IR (Nujol): $\tilde{\nu}_{max}$ = 2954 (Nujol, CH), 2924 (Nujol, CH), 2854 (Nujol, CH), 1567 (vw), 1524 (vs), 1461 (Nujol), 1425 (vs), 1377 (Nujol), 1352 (s), 1320 (s), 1153 (vw), 1105 (w), 1073 (m), 1036 (s), 975 (w), 946 (vw), 891 (vw), 869 (vw), 837 (vw), 779 (vs), 752 (s), 724 (m), 687 (vw), 592 (vw), 537 (vw), 519 (vw), 432 (s), 413 (vs) cm^{-1}. Elemental analysis calcd. (%) for $C_{10}H_{14}MgN_4$ (214.56 g mol^{-1}): C 55.98, H 6.58, N 26.11; found: C 56.87, H 6.83, N 23.59.

[Mg(pz)$_2$]$_n$ (8). Pyrazole (200.0 mg, 2.938 mmol) was dissolved in 10 mL of toluene and a solution of Mg(*n*Bu)$_2$ (1.47 mL, 1.47 mmol, 1 M in Et$_2$O/*n*-hexane) was added dropwise. A white precipitate formed and the reaction mixture was stirred for 3 h. After the solvent was removed under reduced pressure, the white powder was washed three times with *n*-pentane. The white powder was then dryed further under high vacuum for 6 h to remove excess solvent, which gave **8** (204.1 mg, 1.288 mmol, 88%) as a white powder. ^{13}C CP/MAS spectrum (75.47 MHz, MAS at 8 kHz): 141.6 (3/5-*C*(pz)), 103.1 (4-*C*(pz)) ppm. IR (Nujol): $\tilde{\nu}_{max}$ = 2952 (Nujol CH), 2923 (Nujol CH), 2854 (Nujol CH), 1501 (s), 1460 (Nujol) 1411 (s), 1376 (Nujol) 1306 (vw), 1265 (s), 1248 (m), 1169 (m), 1156 (m), 1072 (m), 1053 (vs), 968 (w), 920 (vw), 885 (m), 778 (vs), 761 (vs), 724 (w), 627 (vs), 408 (vs) cm^{-1}. Elemental analysis calcd. (%) for $C_6H_6MgN_4$ (158.45 g mol^{-1}): C 45.48, H 3.82, N 35.36; found: C 46.65, H 4.24, N 33.25.

Mg(CO$_2$·pz^{tBu,tBu})$_2$(thf)$_2$ (1-CO$_2$,thf). A solution of [Mg(pz^{tBu,tBu})$_2$]$_2$ (154 mg, 402 μmol) in THF was stirred under 1 bar CO_2 for 30 min and stored at ambient temperature. Overnight, colorless crystals were obtained suitable for an X-ray structure analysis. The supernatant solution was separated, the residue was dried for 30 min under glovebox atmosphere and **1-CO$_2$,thf** was obtained as colorless crystals (235 mg, 381 μmol, 95%). ^{1}H NMR (400.1 MHz, [D$_8$]THF, 26 °C): δ = 6.08 (s, 2H, 4-*H* (pz)), 1.48 (s, 9H; 3-C(C*H*$_3$)$_3$), 1.24 (s, 9H; 5-C(C*H*$_3$)$_3$) ppm. ^{13}C{H} NMR (100.6 MHz, [D$_8$]THF, 26 °C): δ = 161.5 (3-*C* (pz)), 155.8 (5-*C* (pz)), 149.0 (*C*O$_2$), 104.9 (4-*C* (pz)), 33.9 (*C*(CH$_3$)$_3$), 32.5 (*C*(CH$_3$)$_3$), 30.1 (C(*C*H$_3$)$_3$), 29.7 (C(*C*H$_3$)$_3$) ppm. DRIFTS: $\tilde{\nu}_{max}$ = 3016 (w), 2960 (vs), 2924 (s), 2871 (s), 1745 (vs), 1699 (m), 1579 (vw), 1543 (s), 1516 (w), 1481 (m), 1465 (s), 1456 (s), 1436 (m), 1425 (m), 1393 (m), 1362 (s), 1328 (vs), 1294 (vs), 1250 (vs), 1229 (w), 1208 (m), 1170 (vs), 1143 (s), 1064 (m), 1041 (s), 1021 (m), 1009 (s), 965 (vw), 925 (m), 888 (vs), 850 (s), 815 (vs), 790 (s), 734 (vw), 699 (w), 680 (w), 566 (vw), 522 (w), 484

(w), 461 (m), 308 (w) cm^{-1}. Elemental analysis calcd. (%) for $C_{32}H_{54}MgN_4O_6$ (615.11 g mol^{-1}): C 62.48, H 8.85, N 9.11; found: C 62.14, H 8.89, N 9.08.

Mg(CO$_2$·pz^{iPr,iPr})$_2$(thf)$_2$ (4-CO$_2$,thf). [Mg$_3$(pz^{iPr,iPr})$_6$(thf)$_2$] (**4-thf**, 20 mg, 27 μmol) was dissolved in 0.5 mL of [D$_8$]THF and the atmosphere was exchanged to 1 bar CO_2. The reaction progress was checked by ^{1}H NMR spectroscopy. ^{1}H NMR (400.1 MHz, [D$_8$]THF, 26 °C): δ = 5.99 (s, 1H, 4-*H*(pz)), 3.95 (broad sept., 1H; C*H*(CH$_3$)$_2$), 3.62 (m; 1/4-*H*(thf)), 3.10 (broad sept., 1H; C*H*(CH$_3$)$_2$), 1.77 (m; 2/3-*H*(thf)), 1.20 (broad d, 6H; CH(C*H*$_3$)$_2$), 0.96 (broad d, $^3J_{H,H}$=6.08 Hz, 6H; CH(C*H*$_3$)$_2$) ppm. ^{13}C{H} NMR (100.6 MHz, [D$_8$]THF, 26 °C): δ = 158.7 (5-*C*(pz)), 154.5 (3-*C*(pz)), 149.6 (*C*O$_2$) 100.9 (4-*C*(pz)), 68.0 (1/4-*C*(thf))27.6 (*C*H(CH$_3$)$_2$), 26.8 (*C*H(CH$_3$)$_2$), 26.2 (2/3-*C*(thf)), 23.0 (CH(*C*H$_3$)$_2$), 22.8 (CH(*C*H$_3$)$_2$) ppm.

Mg(CO$_2$·pz^{tBu,tBu})$_2$(thf)$_2$ (1-CO$_2$,thf) + HOOCpz^{tBu,tBu}. [Mg(pz^{tBu,tBu})$_2$(Hpz^{tBu,tBu})$_2$] (**1-Hpz**, 20 mg, 16 μmol) was dissolved in 0.5 mL of [D$_8$]THF and the atmosphere was exchanged to 1 bar CO_2. The reaction progress was checked by ^{1}H NMR spectroscopy. ^{1}H NMR (400.1 MHz, [D$_8$]THF, 26 °C): HOOCpz^{tBu,tBu}: δ = 11.34 (s, 1H, COO*H*), 5.82 (s, 1H, 4-*H*(pz)), 1.28 (s, 9 H, C(C*H*$_3$)$_3$), 1,24 (s, 9 H, C(C*H*$_3$)$_3$) ppm. ^{13}C{H} NMR (100.6 MHz, [D$_8$]THF, 26 °C): HOOCpz^{tBu,tBu}: δ = 161.2 (3,5-*C*(pz)), 152.6 (3,5-*C*(pz)), 96.8 (4-*C*(pz)), 31.2 (*C*(CH$_3$), 31.0 (C(*C*H$_3$), 30.6 (C(*C*H$_3$) ppm.

Mg(CO$_2$·pz^{iPr,iPr})$_2$(thf)$_2$ (4-CO$_2$,thf) + HOOCpz^{iPr,iPr}. [Mg$_3$(pz^{iPr,iPr})$_6$(Hpz^{iPr,iPr})$_2$] (**4-Hpz**, 20 mg, 27 μmol) was dissolved in 0.5 mL of [D$_8$]THF and the atmosphere was changed to 1 bar CO_2. The reaction progress was checked by ^{1}H NMR spectroscopy. ^{1}H NMR (400.1 MHz, [D$_8$]THF, 26 °C): HOOCpz^{iPr,iPr}: δ = 11.22 (s, 1H; *H*OOC), 5.76 (s, 2H; 4-*H*(pz)), 2.92 (sept., $^3J_{H,H}$=6.84 Hz, 2H; C*H*(CH$_3$)$_2$), 2.85 (sept., $^3J_{H,H}$=6.84 Hz, 2H; C*H*(CH$_3$)$_2$), 1.23 (d, $^3J_{H,H}$=6.84 Hz, 12H; CH(C*H*$_3$)$_2$), 1.18 (d, $^3J_{H,H}$=6.84 Hz; CH(C*H*$_3$)$_2$) ppm. ^{13}C{H} NMR (100.6 MHz, [D$_8$]THF, 26 °C): HOOCpz^{iPr,}*i*Pr: δ = 158.7 (5-*C*(pz)), 154.5 (3-*C*(pz)), 97.7 (4-*C*(pz)), 28.7 (*C*H(CH$_3$)$_2$). 26.6 (*C*H(CH$_3$)$_2$), 23.1 (CH(*C*H$_3$)$_2$), 22.7 (CH(*C*H$_3$)$_2$) ppm.

[Mg(CO$_2$·pz^{Me,Me})$_2$]$_n$ (7-CO$_2$). A Schlenk tube was loaded with solid [Mg(pz^{Me,Me})$_2$]$_n$ (95.44 mg, 440.8 μmmol) and the atmosphere changed to 1 bar CO_2. After 3 h **7-CO$_2$** could be collected as a white powder in quantitative yield. ^{13}C CP/MAS spectrum (75.47 MHz, MAS at 8 kHz): 148.6 (5-*C*(pz)), 142.7 (3-*C*(pz)), 108.8 (4-*C*(pz)), 12.3 (*C*H$_3$) ppm. 2954 (Nujol CH), 2924 (Nujol CH), 2854 (Nujol CH), 1744 (vs), 1716 (vs), 1597 (w), 1570 (m), 1523 (vw), 1459 (Nujol), 1420 (s), 1377 (Nujol), 1350 (vs), 1292 (s), 1212 (m), 1157 (w), 1133 (s), 1077 (vw), 1044 (s), 981 (s), 840 (s), 797 (vs), 765 (vs), 724 (s), 632 (w), 585 (vw), 507 (w), 420 (m) cm^{-1}. $C_{12}H_{14}MgN_4O_4$ (302.57 g mol^{-1}): C 47.64, H 4.66, N 18.52; found: C 48.41, H 5.13, N 17.36.

[Mg(CO$_2$·pz)$_2$]$_n$ (8-CO$_2$). A Schlenk tube was loaded with solid [Mg(pz)$_2$]$_n$ (78.57 mg, 495.9 μmmol) and the atmosphere was changed to 1 bar CO_2. After 3 h **8-CO$_2$** could be collected as a white powder in quantitative yield. ^{13}C CP/MAS spectrum (75.47 MHz, MAS at 8 kHz): 150.2 (O*C*O), 139.1 (5-*C*(pz)), 128.6 (3-*C*(pz)), 107.2 (4-*C*(pz)) ppm. IR (Nujol): $\tilde{\nu}_{max}$ = 3131 (w), 2954 (Nujol CH), 2924 (Nujol CH), 2854 (Nujol CH), 1718 (vs), 1709 (vs), 1590 (vw), 1520 (w), 1460 (Nujol), 1398 (vs), 1376 (Nujol), 1366 (vs), 1350 (vs), 1317 (vs), 1287 (vs), 1238 (m), 1198 (s), 1169 (vw), 1074 (w), 1047 (vs), 977 (m), 922 (w), 820 (vs), 793 (vs), 767 (vs), 724 (m), 654 (m), 631 (vw), 603 (w), 496 (w) cm^{-1}. $C_8H_6MgN_4O_4$ (246.47 g mol^{-1}): C 38.99, H 2.45, N 22.73; found: C 40.08, H 2.85, N 22.44.

General procedure of the catalytic conversion of epoxides to cyclic carbonates. Representative example: A small Schlenk tube was charged with [Mg(pz^{tBu,tBu})$_2$]$_2$ (10.0 mg, 26.1 μmol) and TBAB (16.8 mg, 52.2 μmol) and dissolved in the corresponding epoxide (5.224 mmol). The atmosphere was exchanged with 1 bar CO_2 and the reaction mixture was stirred for 24 h. The progress of the reaction was determined via ^{1}H NMR spectroscopy by dissolving the reaction mixture in $CDCl_3$.

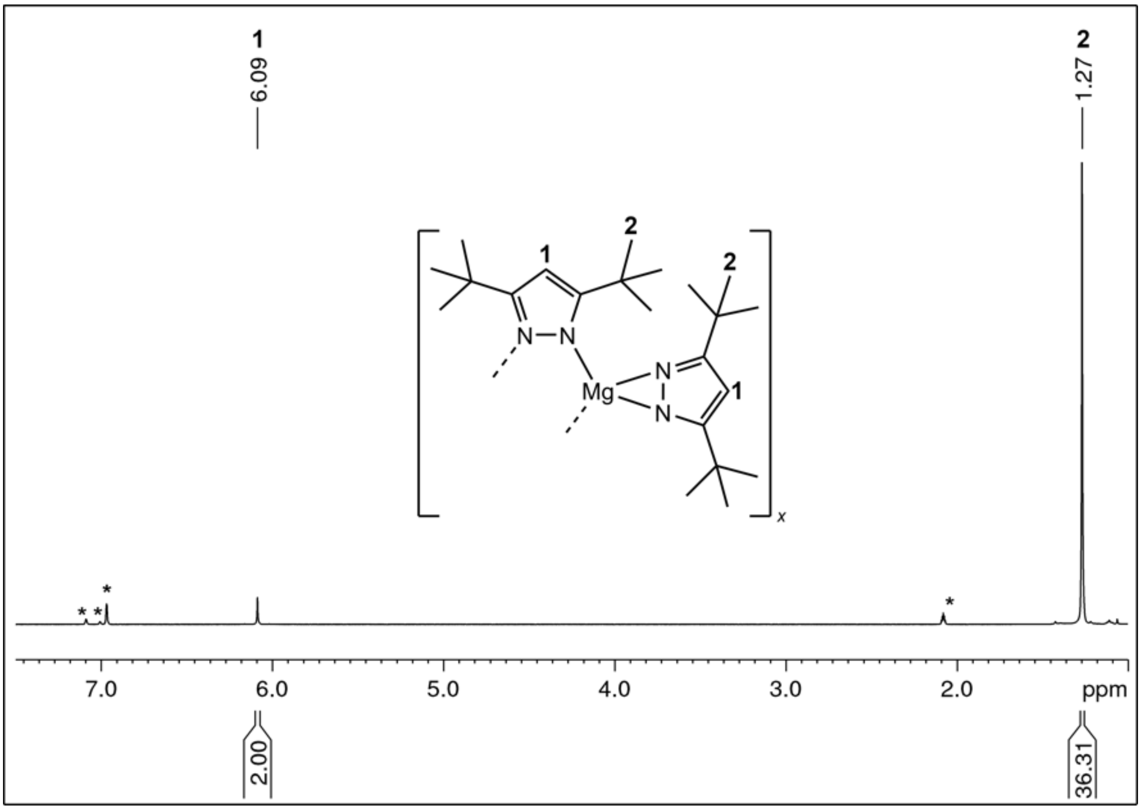

Figure S1. ^{1}H NMR spectrum (26 °C, 400.11 MHz, [D$_8$]toluene) of [Mg(pz^{tBu,tBu})$_2$]$_2$ (**1**).

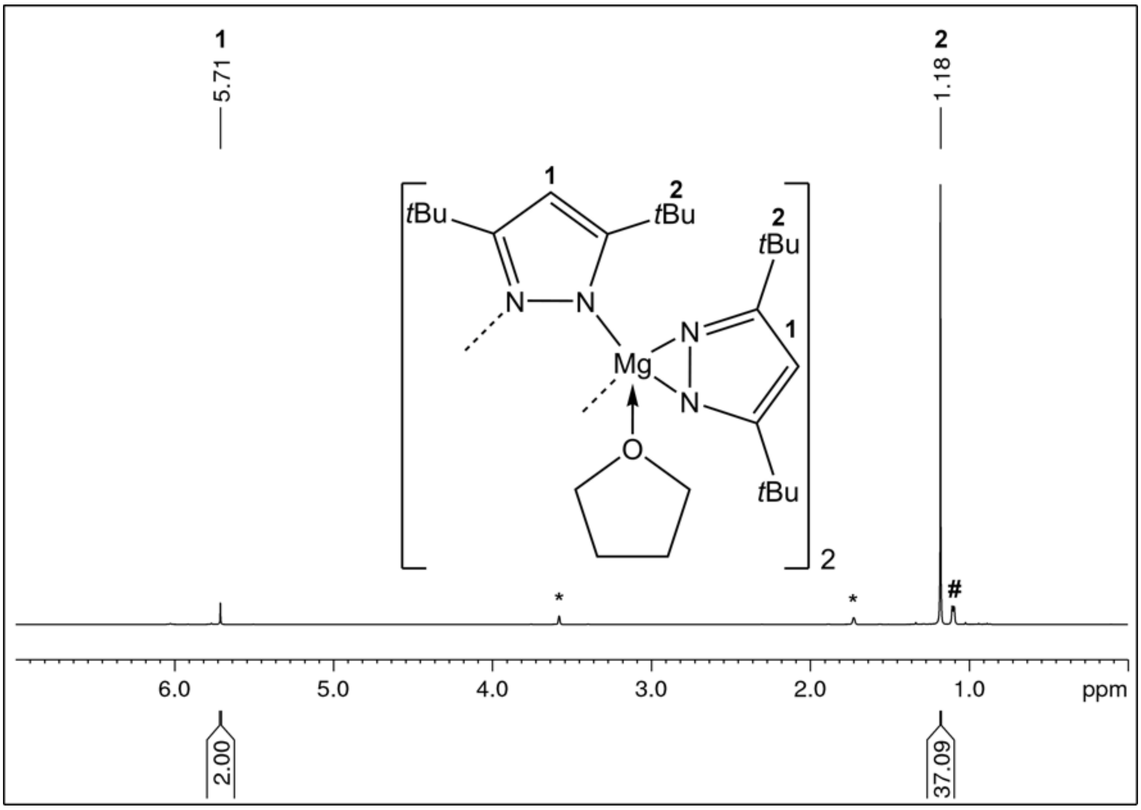

Figure S2. ^{1}H NMR spectrum (26 °C, 400.11 MHz, [D$_8$]THF) of [Mg(pz^{tBu,tBu})$_2$(thf)]$_2$ (**1-thf**).

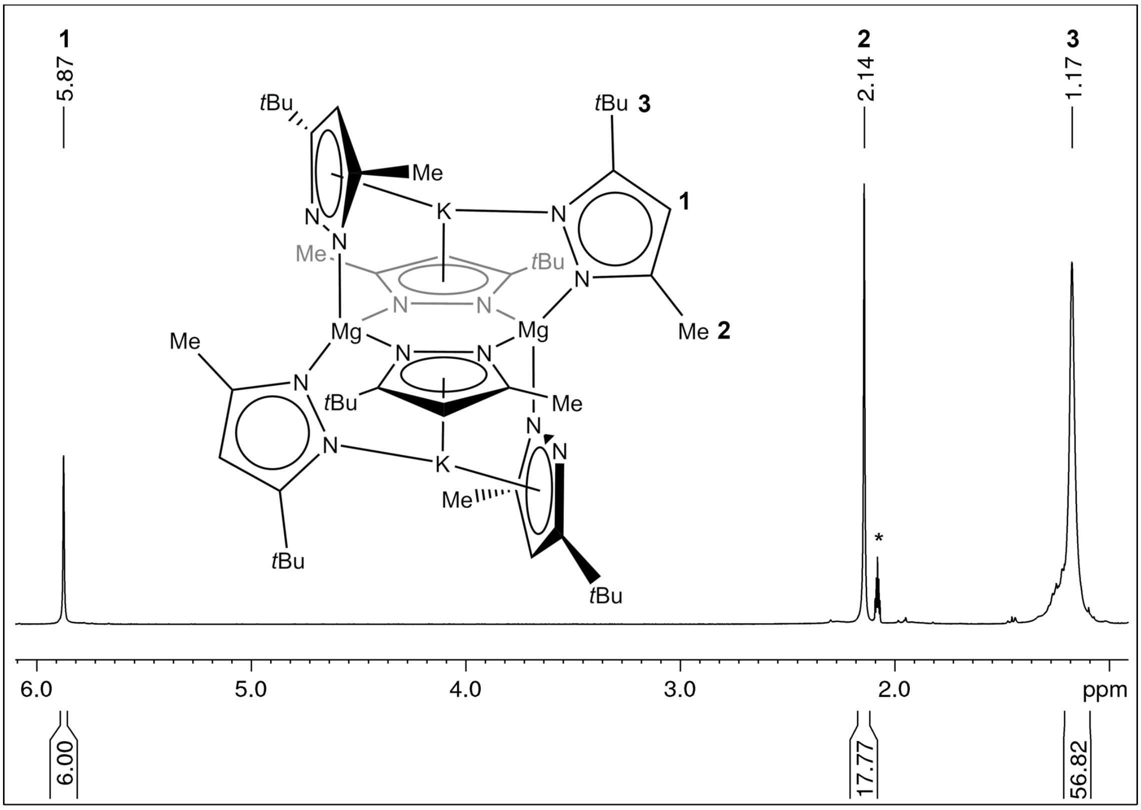

Figure S3. ^{1}H NMR spectrum (26 °C, 400.11 MHz, [D$_8$]toluene) of [KMg(pz^{tBu,Me})$_3$]$_2$ (**2**).

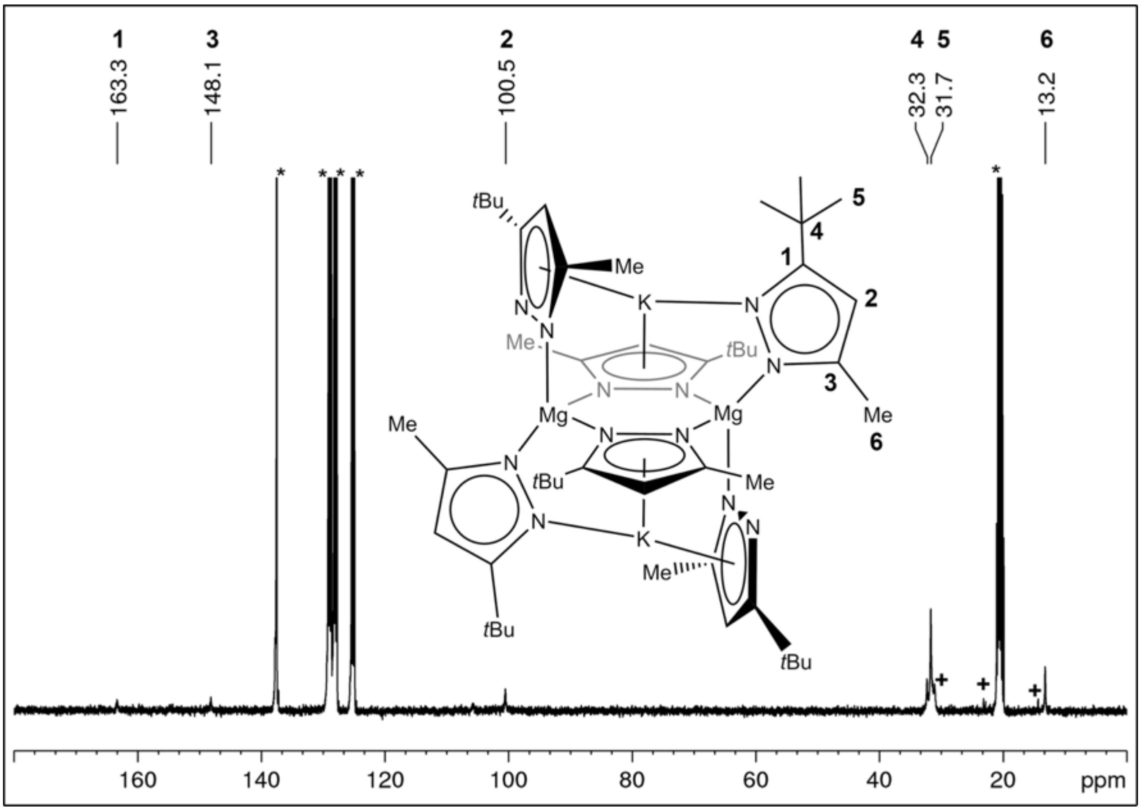

Figure S4. $^{13}C\{^{1}H\}$ NMR spectrum (26 °C, 100.61 MHz, [D$_8$]toluene) of [KMg(pz^{tBu,Me})$_3$]$_2$ (**2**) (**+** *n*-hexane).

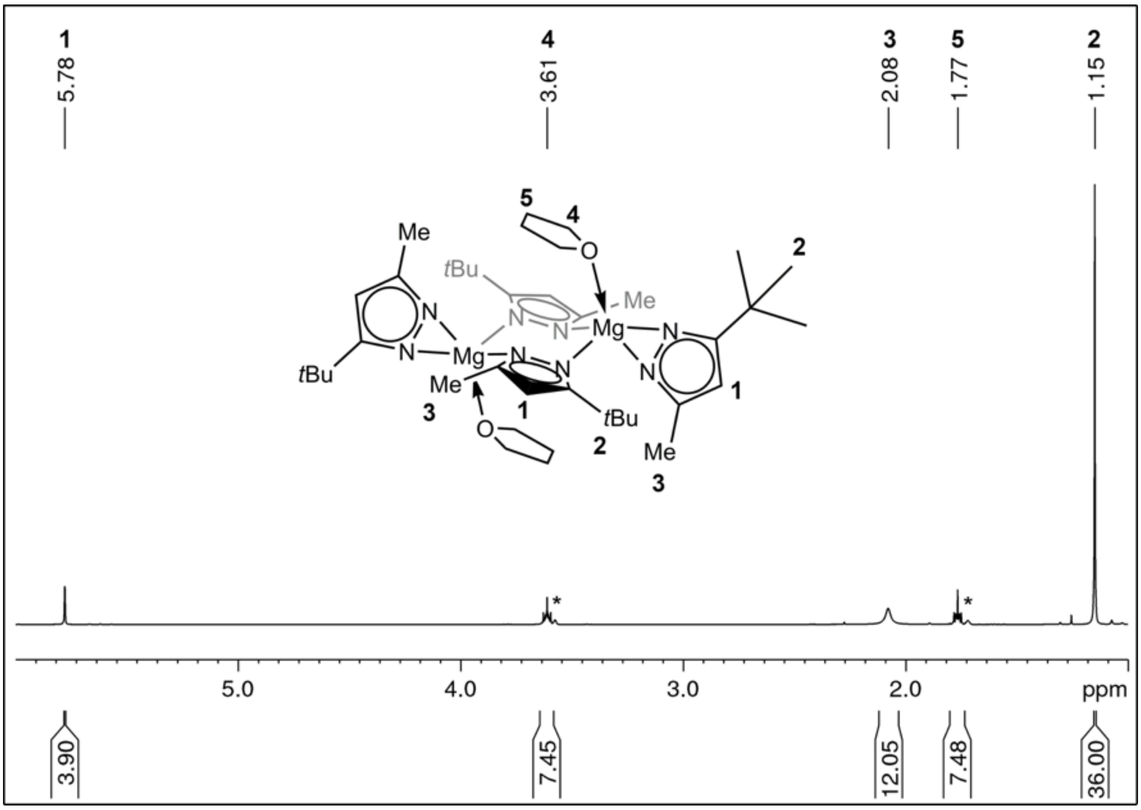

Figure S5. ^{1}H NMR spectrum (26 °C, 400.11 MHz, [D$_8$]THF) of [Mg(pz^{tBu,Me})$_2$(thf)]$_2$ (**3-thf**).

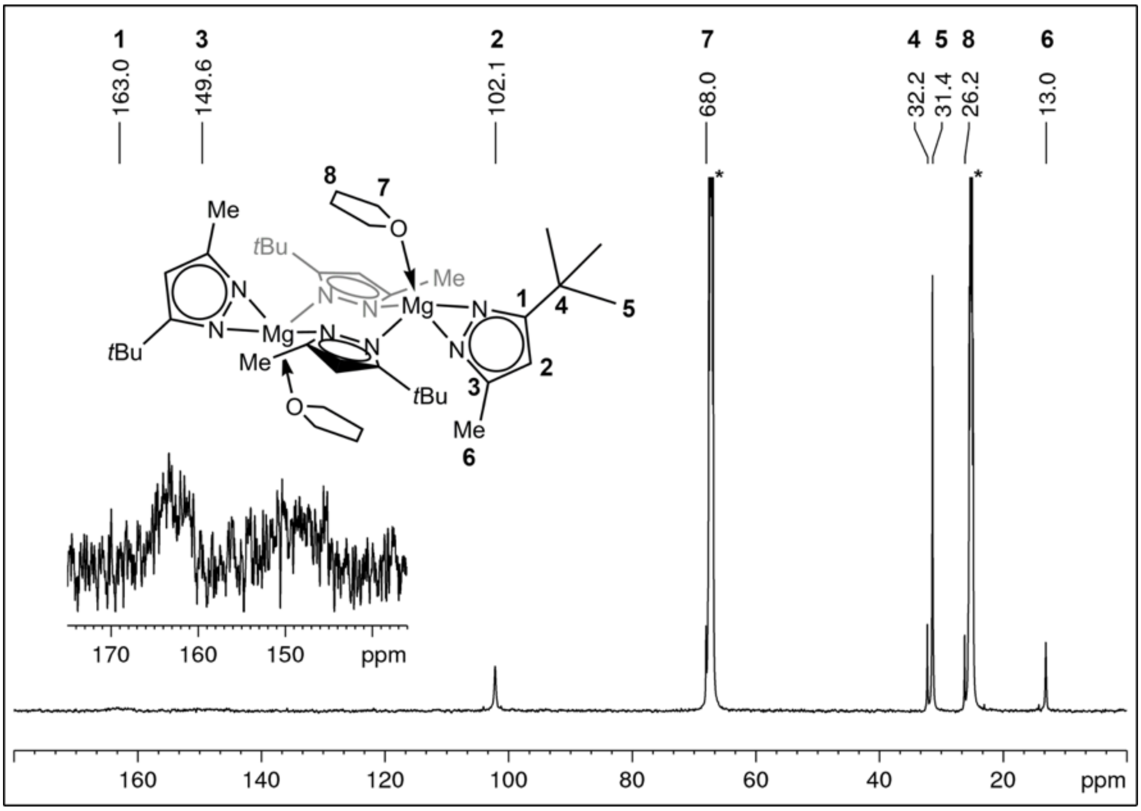

Figure S6. ^{13}C{^{1}H} NMR spectrum (26 °C, 125.76 MHz, [D$_8$]THF) of [Mg(pz^{tBu,Me})$_2$(thf)]$_2$ (**3-thf**).

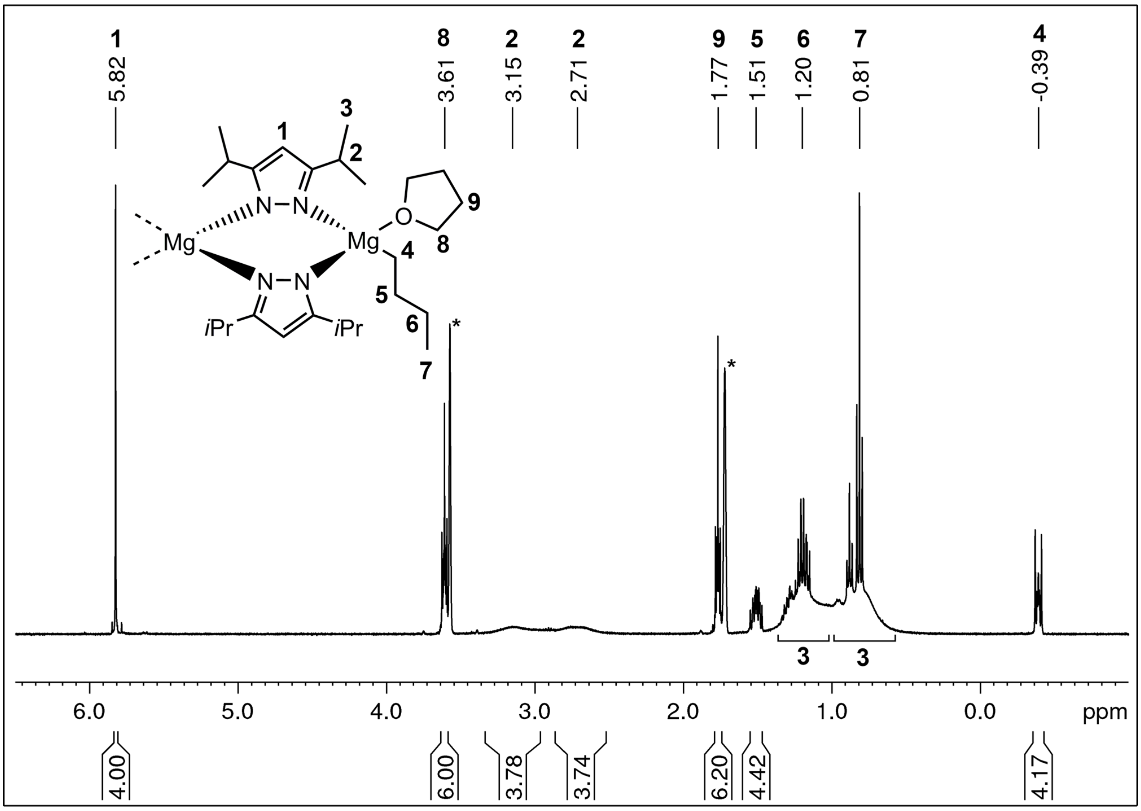

Figure S7. ^{1}H NMR spectrum (26 °C, 400.11 MHz, [D$_8$]THF) of [Mg$_3$(pz^{iPr,iPr})$_4$(nBu)$_2$(thf)$_2$] (**5**).

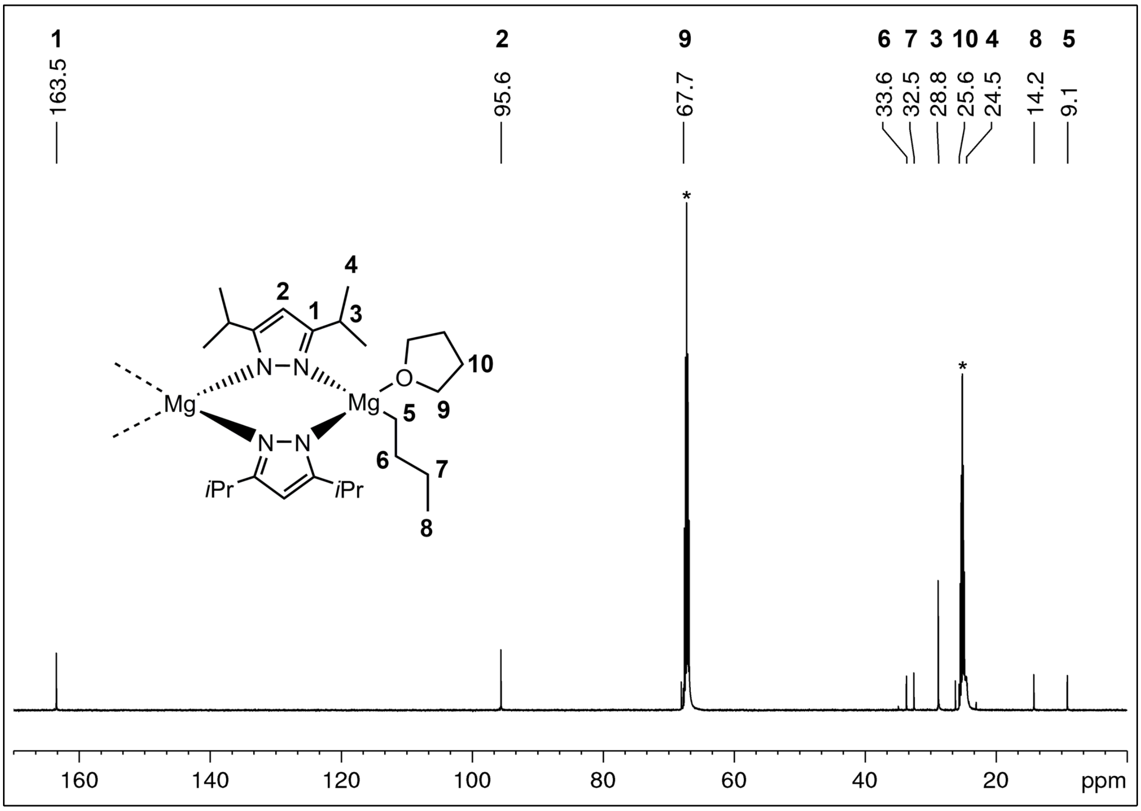

Figure S8. ^{13}C{^{1}H} NMR spectrum (26 °C, 125.76 MHz, [D$_8$]THF) of [Mg$_3$(pz^{iPr,iPr})$_4$(nBu)$_2$(thf)$_2$] (**5**).

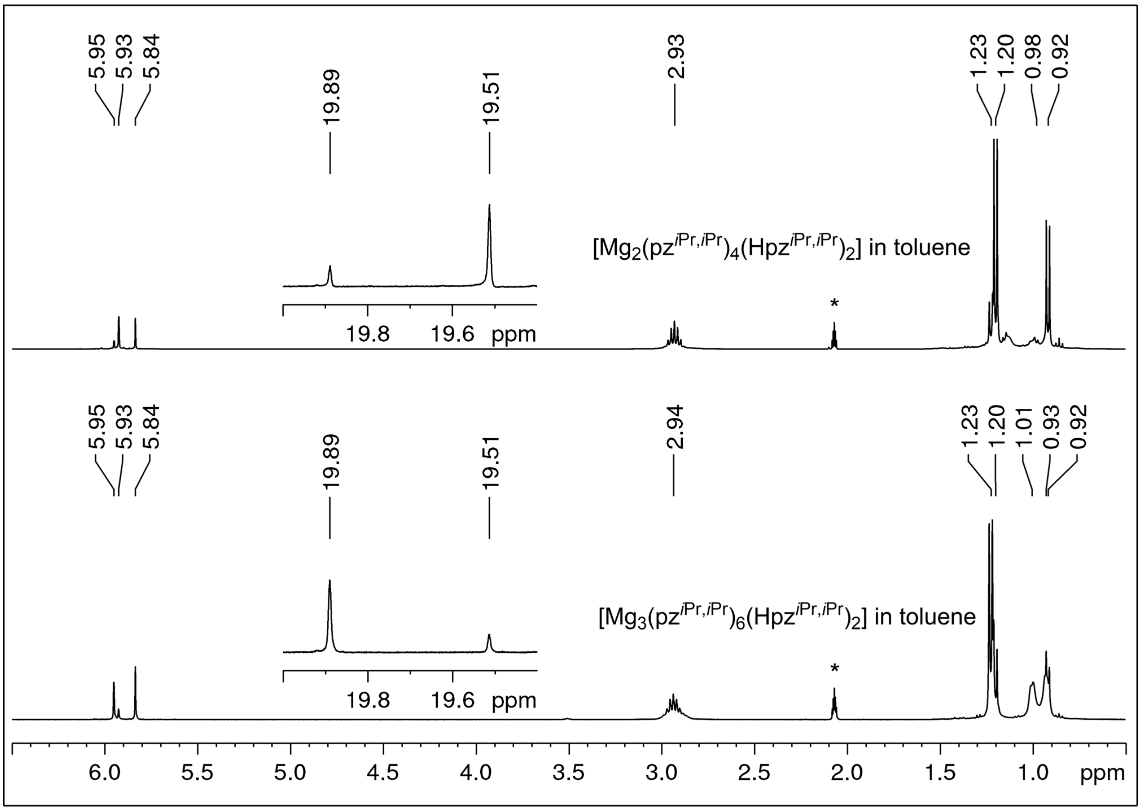

Figure S9. Comparison of ^{1}H NMR spectra (26 °C, 400.11 MHz, [D$_8$]toluene) of [Mg$_3$(pz^{iPr,iPr})$_6$(Hpz^{iPr,iPr})$_2$] (**4a-Hpz**, bottom) and [Mg$_2$(pz^{iPr,iPr})$_4$(Hpz^{iPr,iPr})$_2$] (**4-Hpz**, top).

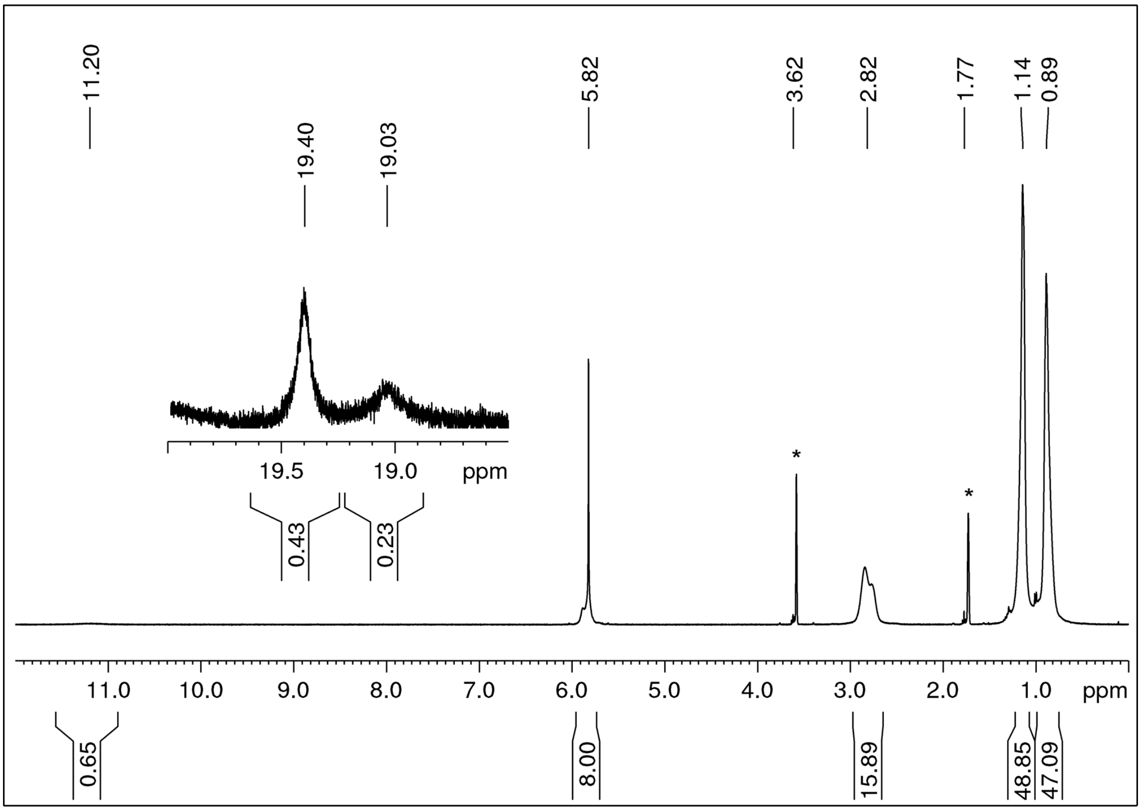

Figure S10. ^{1}H NMR spectrum (26 °C, 400.11 MHz, [D$_8$]THF) of [Mg$_3$(pz^{iPr,iPr})$_6$(Hpz^{iPr,iPr})$_2$] (**4a-Hpz**).

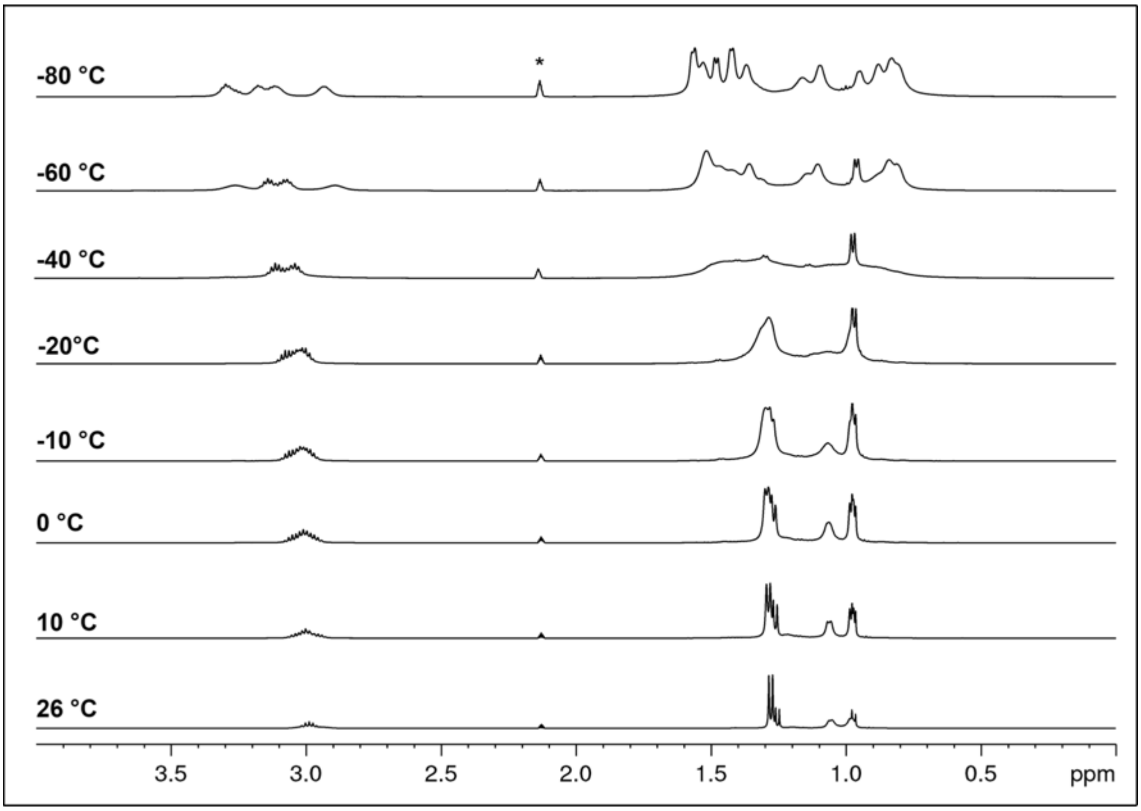

Figure S11. VT ^{1}H NMR spectra (500.13 MHz, [D$_8$]THF) of [Mg$_3$(pz^{iPr,iPr})$_6$(Hpz^{iPr,iPr})$_2$] (**4a-Hpz**) in the range of 26 °C to –80 °C.

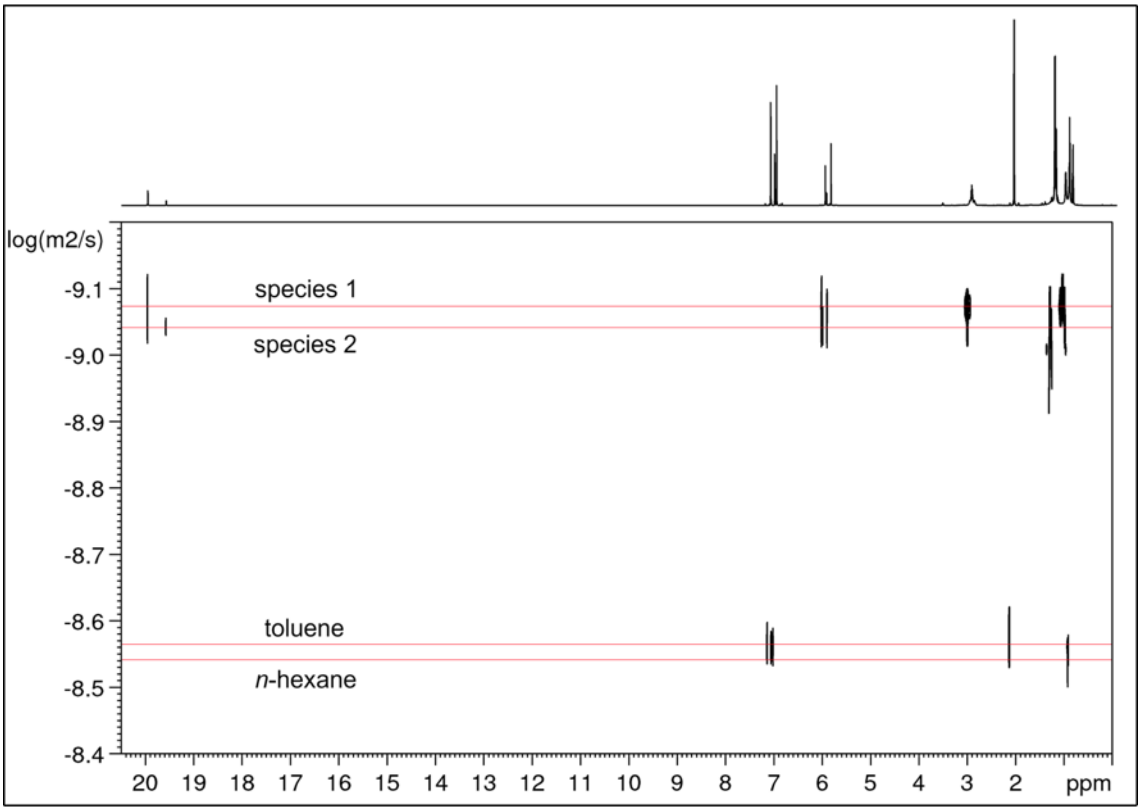

Figure S12. ^{1}H DOSY NMR spectrum (26 °C, 700.29 MHz, [D$_8$]toluene) of [Mg$_3$(pz^{iPr,iPr})$_6$(Hpz^{iPr,iPr})$_2$] (**4a-Hpz**). **4a-Hpz** disassociates into two different species in solution. Molecular weight calculation see below.

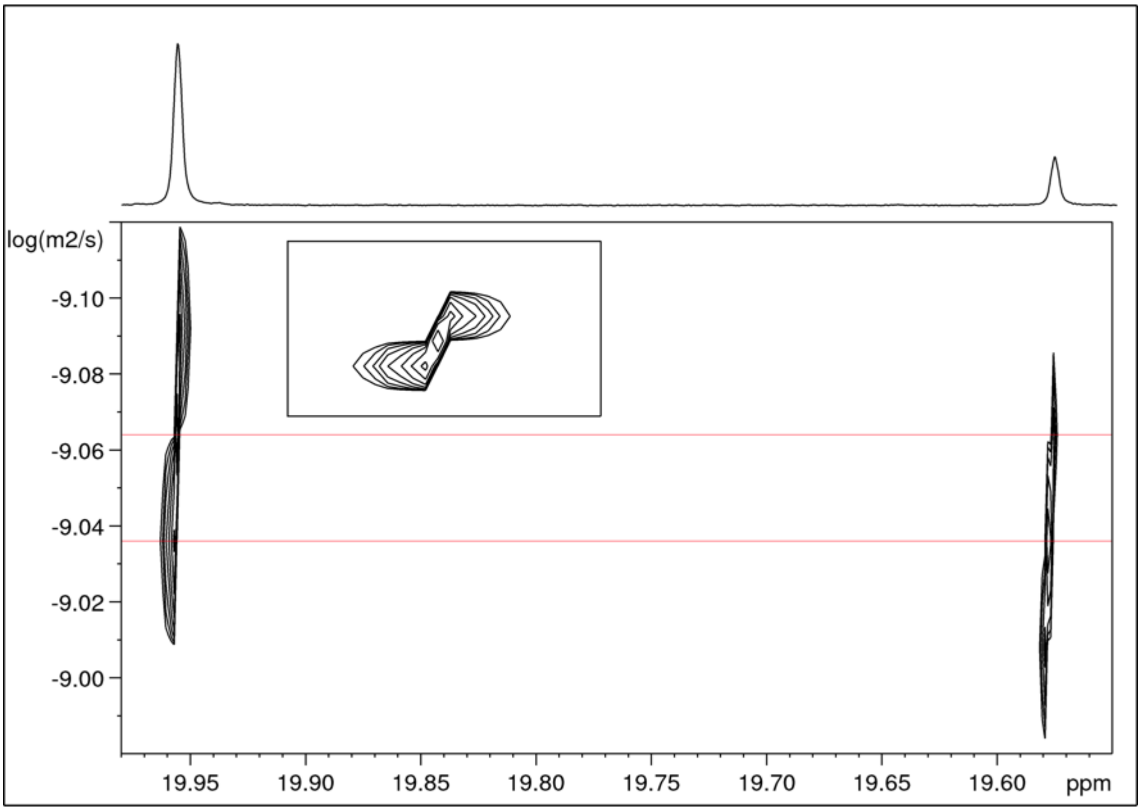

Figure S13. ^{1}H DOSY NMR spectrum (26 °C, 700.29 MHz, [D$_8$]toluene) of [Mg$_3$(pz^{iPr,iPr})$_6$(Hpz^{iPr,iPr})$_2$] (**4a-Hpz**) in the region of strongly low field shifted H–N signals.

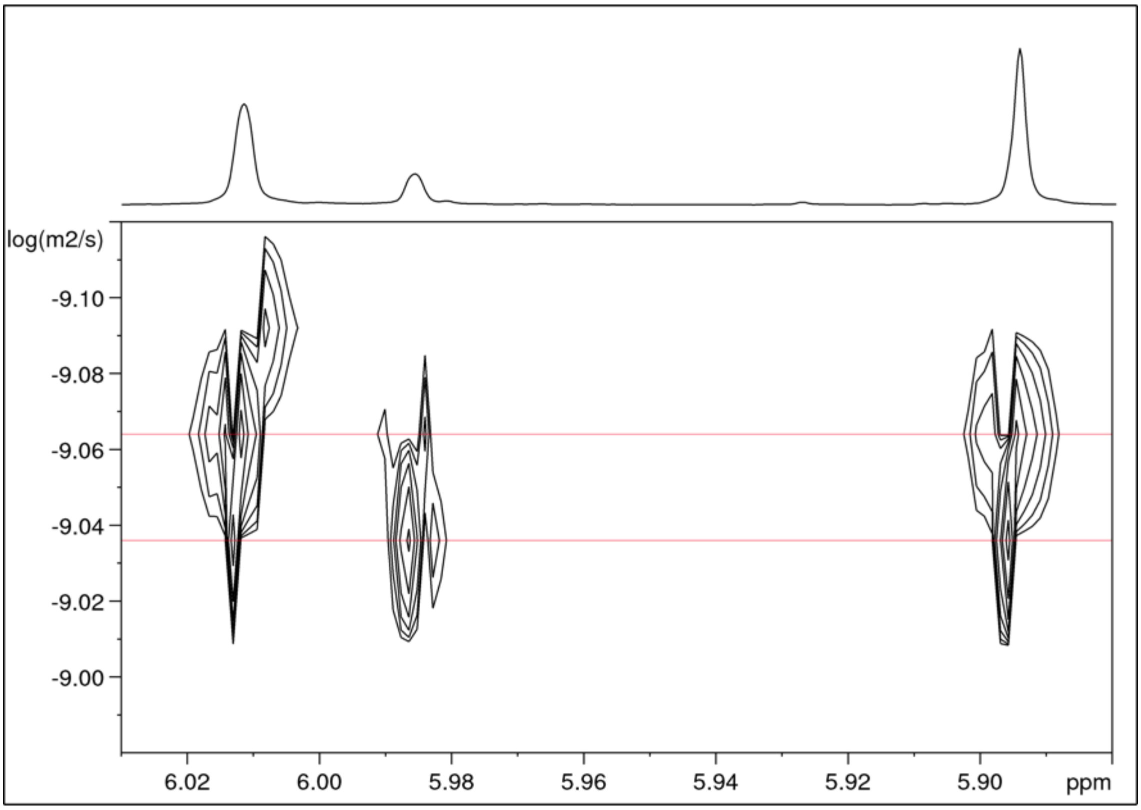

Figure S14. ^{1}H DOSY NMR spectrum (26 °C, 700.29 MHz, [D$_8$]toluene) of [Mg$_3$(pz^{iPr,iPr})$_6$(Hpz^{iPr,iPr})$_2$] (**4a-Hpz**) in the aromatic region.

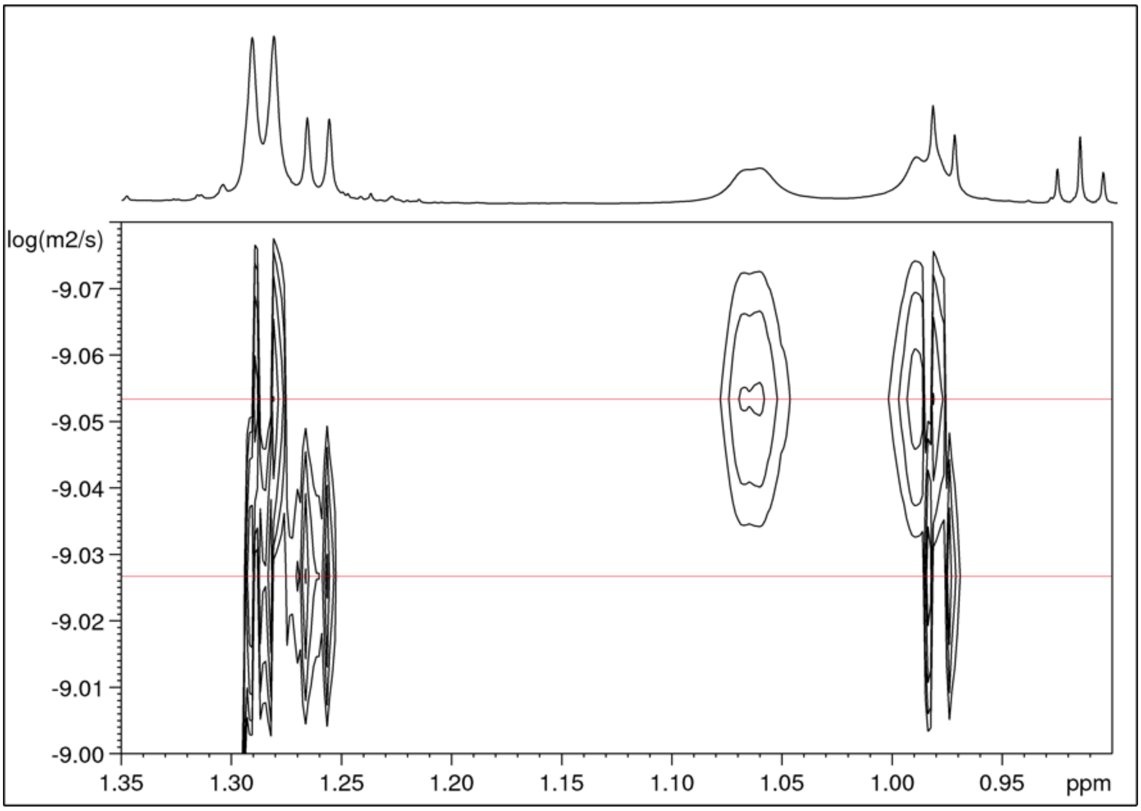

Figure S15. ^{1}H DOSY NMR spectrum (26 °C, 700.29 MHz, [D$_8$]toluene) of [Mg$_3$(pz^{iPr,iPr})$_6$(Hpz^{iPr,iPr})$_2$] (**4a-Hpz**) in the region of *i*Pr moities.

DOSY Experiment Data of $[Mg_3(pz^{iPr,iPr})_6(Hpz^{iPr,iPr})_2]$ (4a-Hpz)

The ^{1}H NMR spectrum of **4a-Hpz** revealed that dissolving **4a-Hpz** in [D$_8$]toluene leads to fragmentation into two distinct species. The same fragmentation species were observed when **4-Hpz** was dissolved in [D$_8$]toluene but the integrals of the two species were reversed (see Figure S7). To examine this fragmentation behavior further a ^{1}H DOSY NMR experiment was carried out for **4-Hpz** and **4a-Hpz**. This experiment confirmed two fragmentation species with two distinct diffusion coefficients.

Table S1. Diffusion coefficients of the fragmentation species of **4-Hpz** and **4a-Hpz** in [D$_6$]toluene determined via ^{1}H DOSY NMR experiment.

4a-Hpz				4-Hpz			
Substance A	D (average)	Variance	pD (average)	Substance A	D (average)	Variance	pD (average)
T1T2	7.284E-10	0.595%	-9.1377	T1T2	8.262E-10	0.894%	-9.0829
Contin	7.445E-10	1.491%	-9.1281	Contin	7.843E-10	3.067%	-9.1055
Expt	7.843E-10	2.020%	-9.1055	Expt	8.607E-10	1.476%	-9.0651
Substance B	D (average)	Variance	pD (average)	Substance B	D (average)	Variance	pD (average)
T1T2	7.845E-10	1.31%	-9.1054	T1T2	9.050E-10	1.030%	-9.0434
Contin	8.160E-10	0.48%	-9.0883	Contin	8.979E-10	3.577%	-9.0468
Expt	8.359E-10	0.10%	-9.0778	Expt	9.649E-10	0.073%	-9.0155

The diffusion coefficients (D) were determined via three different evaluation methods provided by Topspin. The T1/T2 module uses a numeric calculation for D, whereas the contin/exponential evaluation uses manual signal picking. D was calculated from the measured pD values. The D values listed in Table S1 are averaged over all signals assigned to one species that are not overlapping with signals from another species. As expected, the DOSY revealed two species with distinct diffusion coefficient of logD = –9.13 log($m^2 \cdot s^{-1}$) (**A**) and –9.09 log($m^2 \cdot s^{-1}$) (**B**)). The signal at 5.84 ppm is an overlapping signal from both species hence the unchanged intensity between the spectra of the reaction of **4-Hpz** and **4a-Hpz**.

Table S2. Molecular weight calculation via the measured diffusion coefficients of the fragmentation species of **6** and **4a-Hpz** with an approximation of a highly compact sphere.

	4a-Hpz			4-Hpz		
Substance A	M (calc.)	M(found)	Deviation	M (calc.)	M(found)	Deviation
T1T2	958.02	934	3%	805.78	794	1%
Contin	958.02	960	0%	805.78	902	-12%
Expt	958.02	888	7%	805.78	802	0%
Substance B	M (calc.)	M(found)	Deviation	M (calc.)	M(found)	Deviation
T1T2	805.78	804	0%	631.25	662	-5%
Contin	805.78	800	1%	631.25	688	-9%
Expt	805.78	781	3%	631.25	639	-1%

For the molecular weight calculation the method described by Stalke was used.[11] There are nine different fragments possible for **4a-Hpz** and six for **4-Hpz** (see Scheme S1). Because there must be at least one hydrogen bond, three fragments could be eliminated for **4a-Hpz** and two for **4-Hpz**. Using an approximation of a highly compact sphere all three evaluation methods led to the same two fragments 4 (M = 958.02 g/mol) and 5 (M = 805.78 g/mol) for complex **4a-Hpz** (see Table S2). In all six cases the deviation lies within the methods error of 9%. However, calculating the molecular weight of the fragmentation species of **4-Hpz** led to the fragments 5 (M = 805.78 g/mol) and 7 (M = 631.25 g/mol). This indicates that either **4a-Hpz** and **4-Hpz** fragment into different species with a comparable chemical shift or the method varies with the used substrate.

Table S3. Molecular weight calculation via the measured diffusion coefficients of the fragmentation species of **4-Hpz** and **4a-Hpz** with an approximation of an ellipsoid.

	4a-Hpz			4-Hpz		
Substance A	M (calc.)	M(found)	Deviation	M (calc.)	M(found)	Deviation
T1T2	631.25	603	4%	631.25	599	5%
Contin	631.25	668	-6%	631.25	665	-5%
Expt	631.25	636	-1%	631.25	604	4%
Substance B	M (calc.)	M(found)	Deviation	M (calc.)	M(found)	Deviation
T1T2	479.01	554	-16%	479.01	516	-8%
Contin	479.01	588	-23%	479.01	533	-11%
Expt	479.01	565	-18%	479.01	501	-5%

Using an approximation of an ellipsoid leads to higher deviations for species 2. For complex **4a-Hpz**, the deviation is too high to assign it to one of the six possible fragments. For complex **4-Hpz**, the T1/T2 and exponential evaluation of species 2 could be assigned to fragment 8 (M = 479.01 g/mol). In the case of species 1 both complexes **4a-Hpz** and **4-Hpz** led to fragment 7 (M = 631.25 g/mol).

Depending on the approximation method for the molecular weight calculation the fragments are either fragment 4, 5 and 7 for a highly compact sphere or 7 and 8 for an ellipsoid.

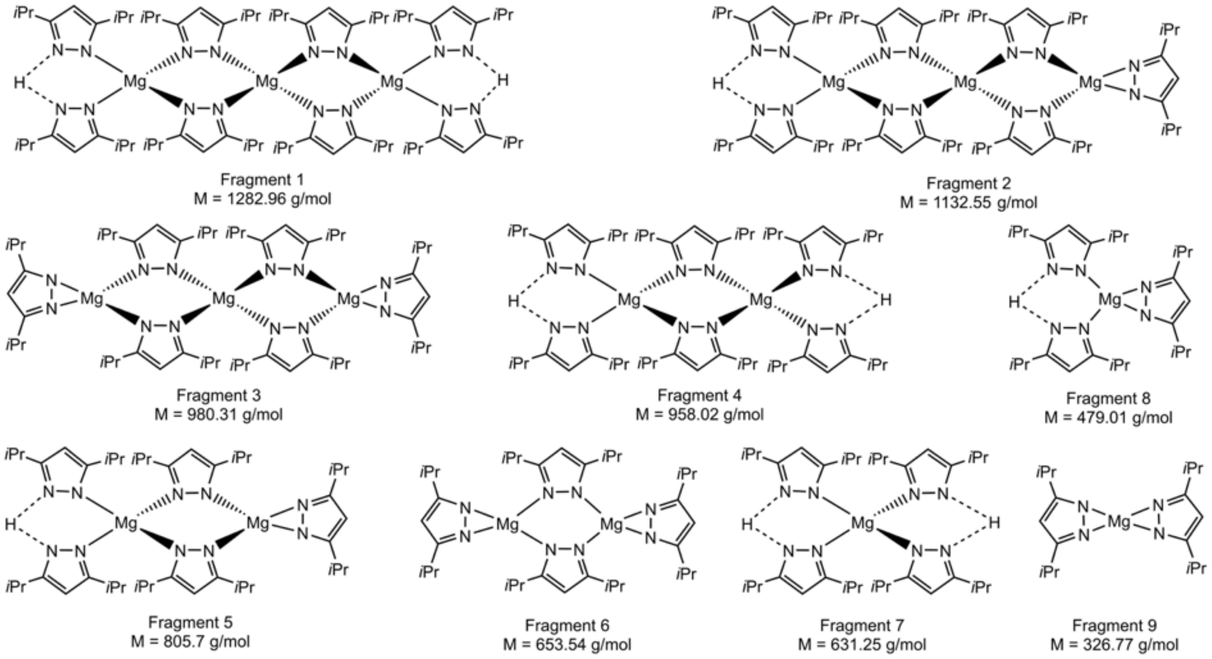

Scheme S1. Possible fragmentation species for **4a-Hpz** dissolved in toluene.

Since a crystal structure of any fragmentation product of **4-Hpz** or **4a-Hpz** could not be obtained, a comparison of the calculated and measured hydrodynamic radii was not possible.

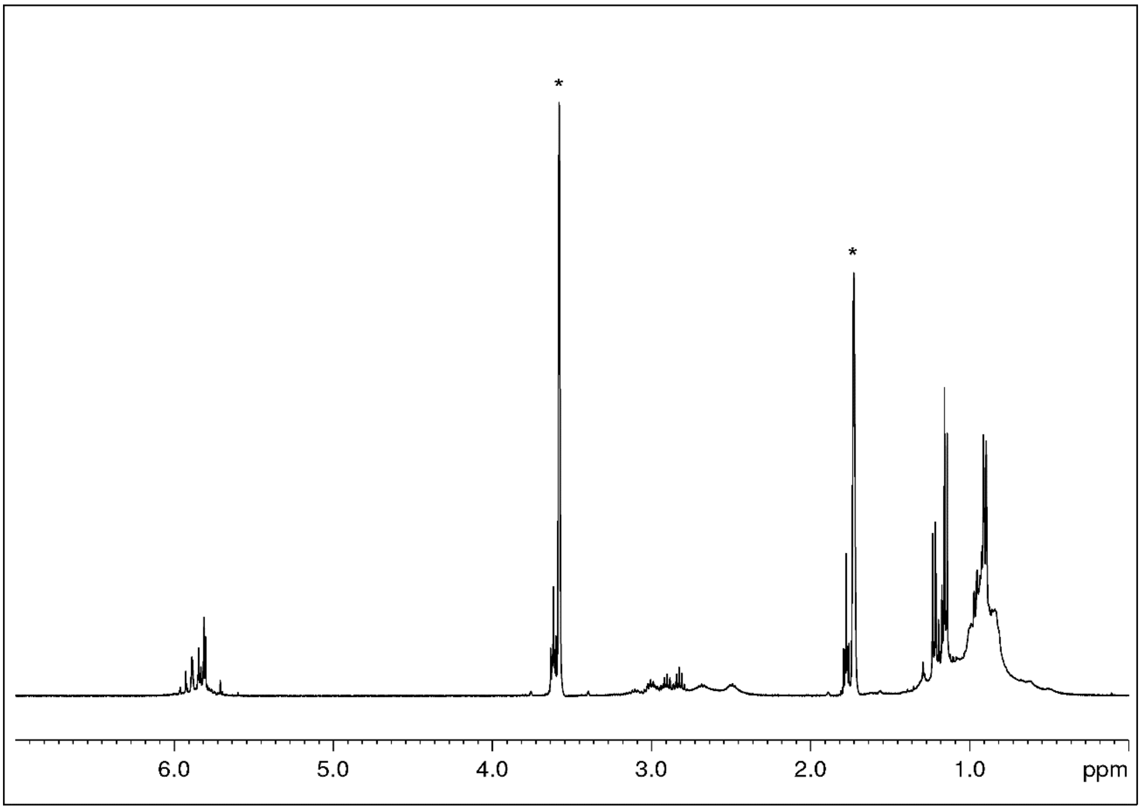

Figure S16. ^{1}H NMR spectrum (26 °C, 400.12 MHz, [D$_8$]THF) of [Mg$_3$(pz^{iPr,iPr})$_6$(thf)$_2$] (**4-thf**).

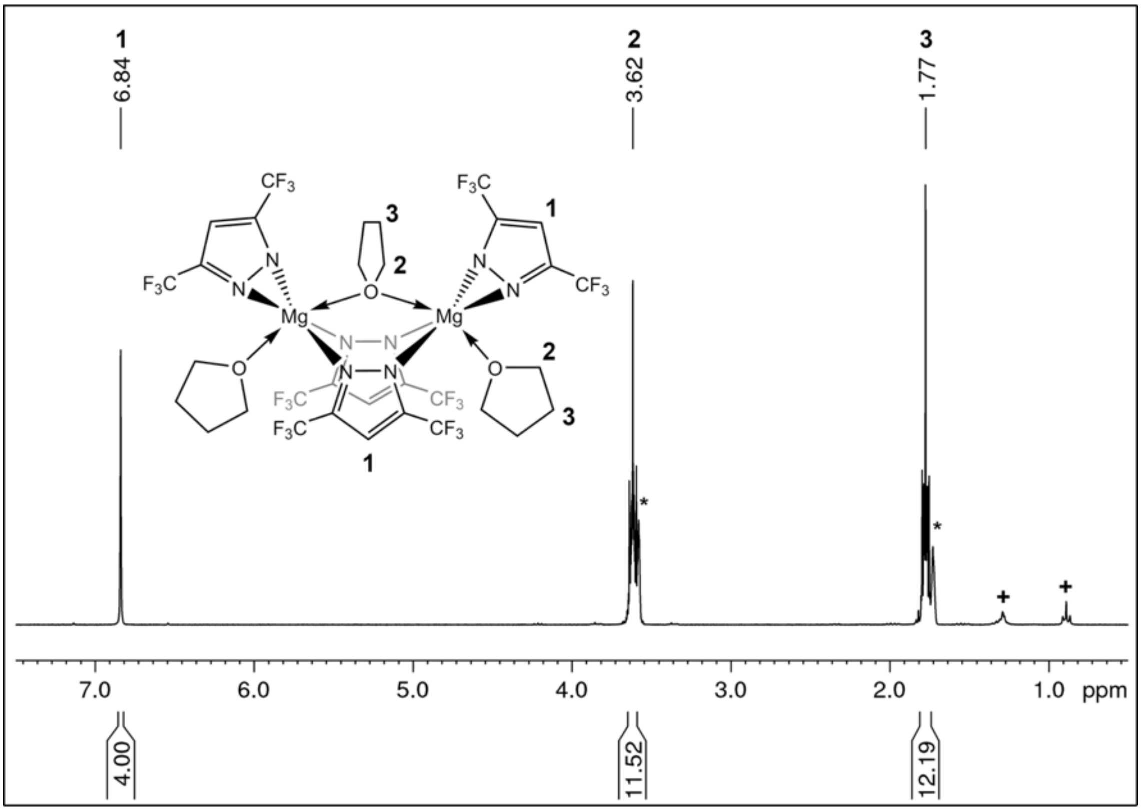

Figure S17. ^{1}H NMR spectrum (26 °C, 300.13 MHz, [D$_8$]THF) of [Mg$_2$(pz^{CF_3,CF_3})$_4$(thf)$_3$] (**6-thf**) (+ *n*-hexane).

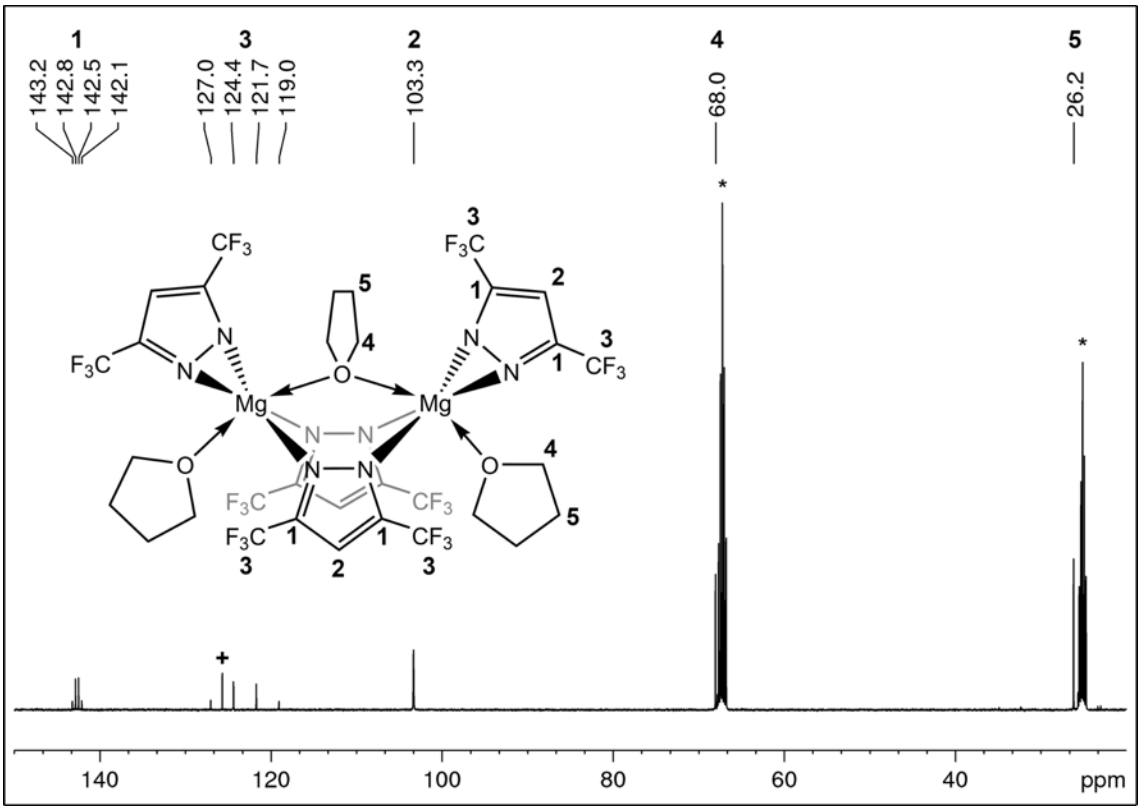

Figure S18. $^{13}C\{^{1}H\}$ NMR spectrum (26 °C, 100.61 MHz, $[D_8]THF$) of $[Mg_2(pz^{CF_3,CF_3})_4(thf)_3]$ (**6-thf**) (+ solv. CO_2).

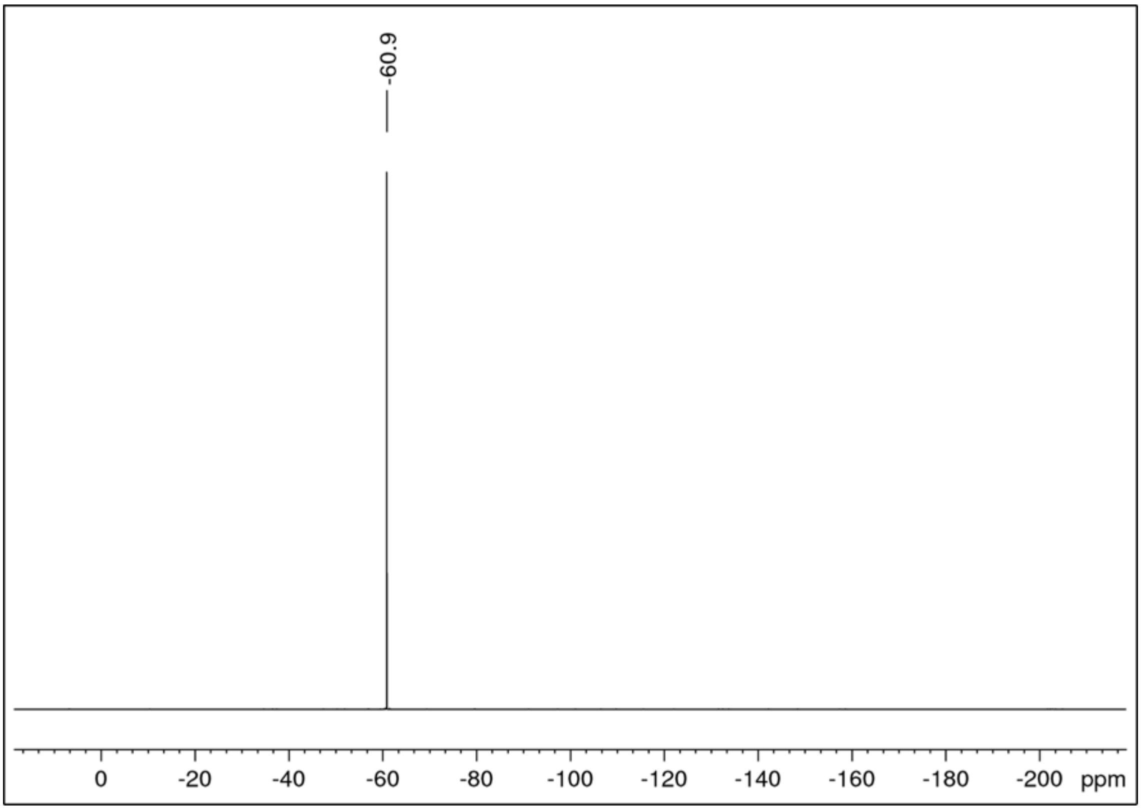

Figure S19. $^{19}F\{^{1}H\}$ NMR spectrum (26 °C, 282.40 MHz, $[D_8]THF$) of $[Mg_2(pz^{CF_3,CF_3})_4(thf)_3]$ (**6-thf**).

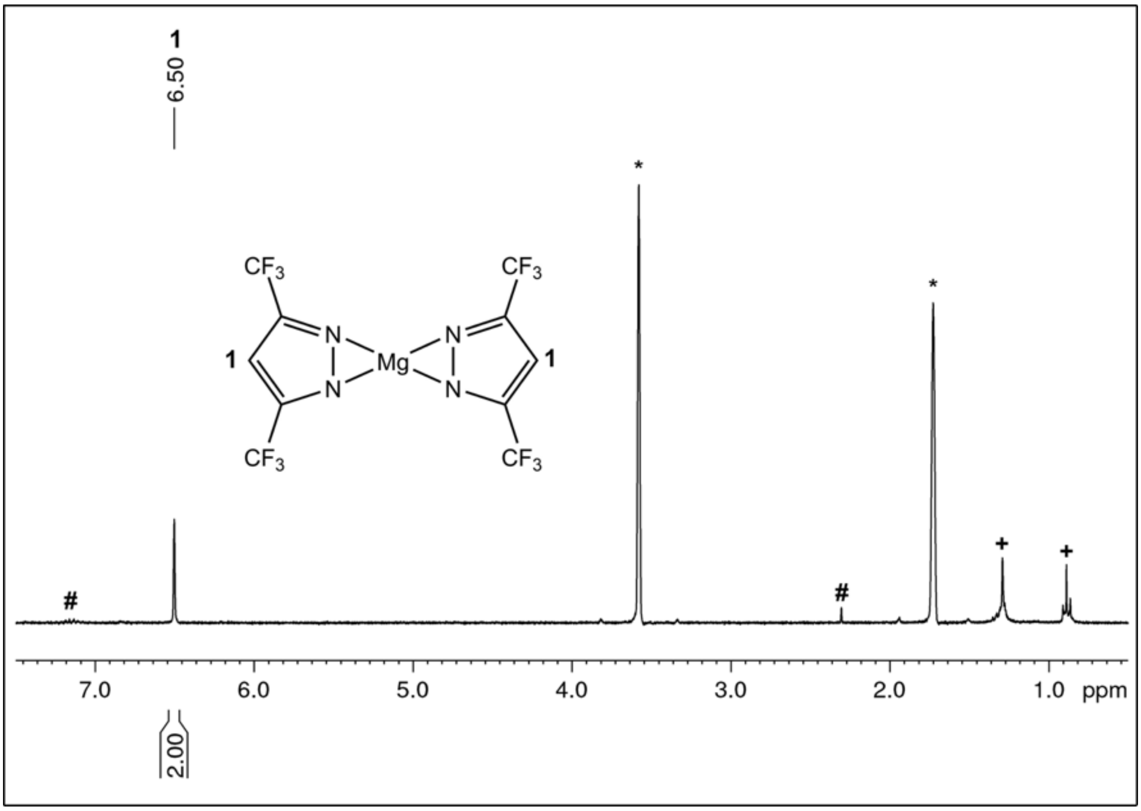

Figure S20. ^{1}H NMR spectrum (26 °C, 300.13 MHz, $[D_8]$THF) of $[Mg(pz^{CF_3,CF_3})_2]_n$ (**6**) (+ *n*-hexane/# impurities).

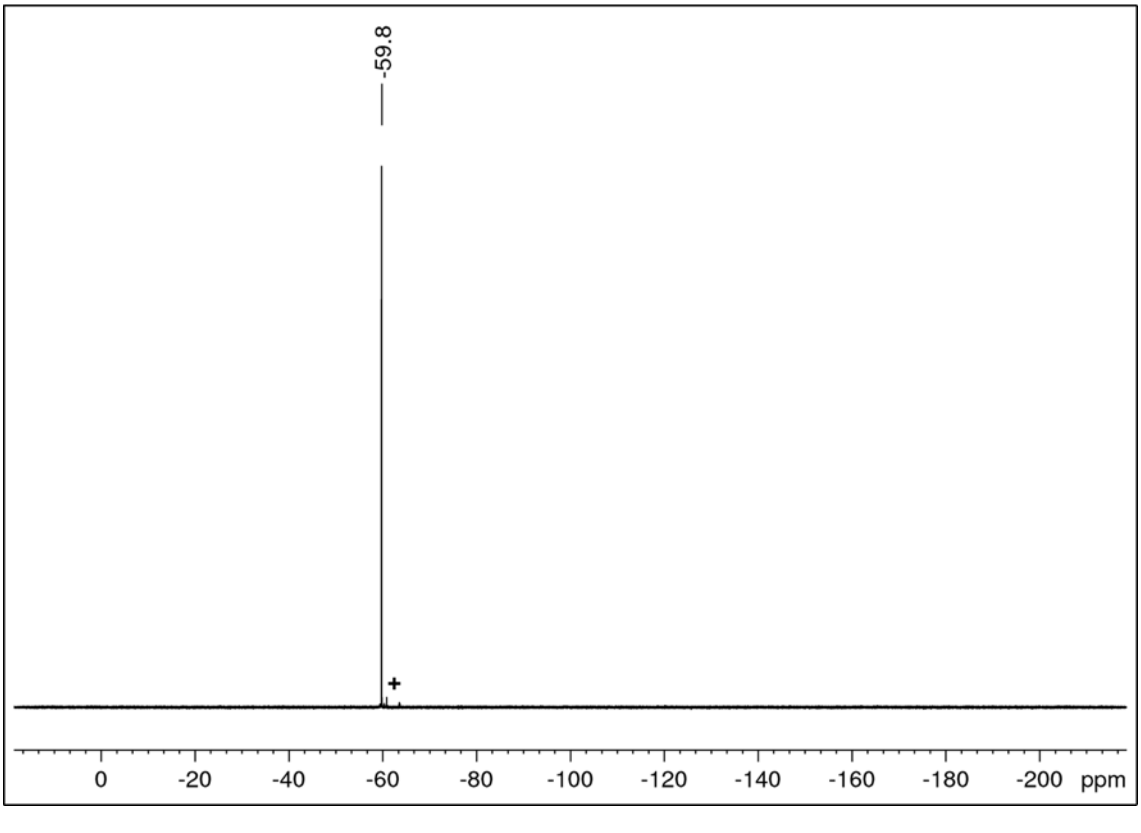

Figure S21. ^{19}F NMR spectrum (26 °C, 282.40 MHz, $[D_8]$THF) of $[Mg(pz^{CF_3,CF_3})_2]_n$ (**6**) (+ impurities).

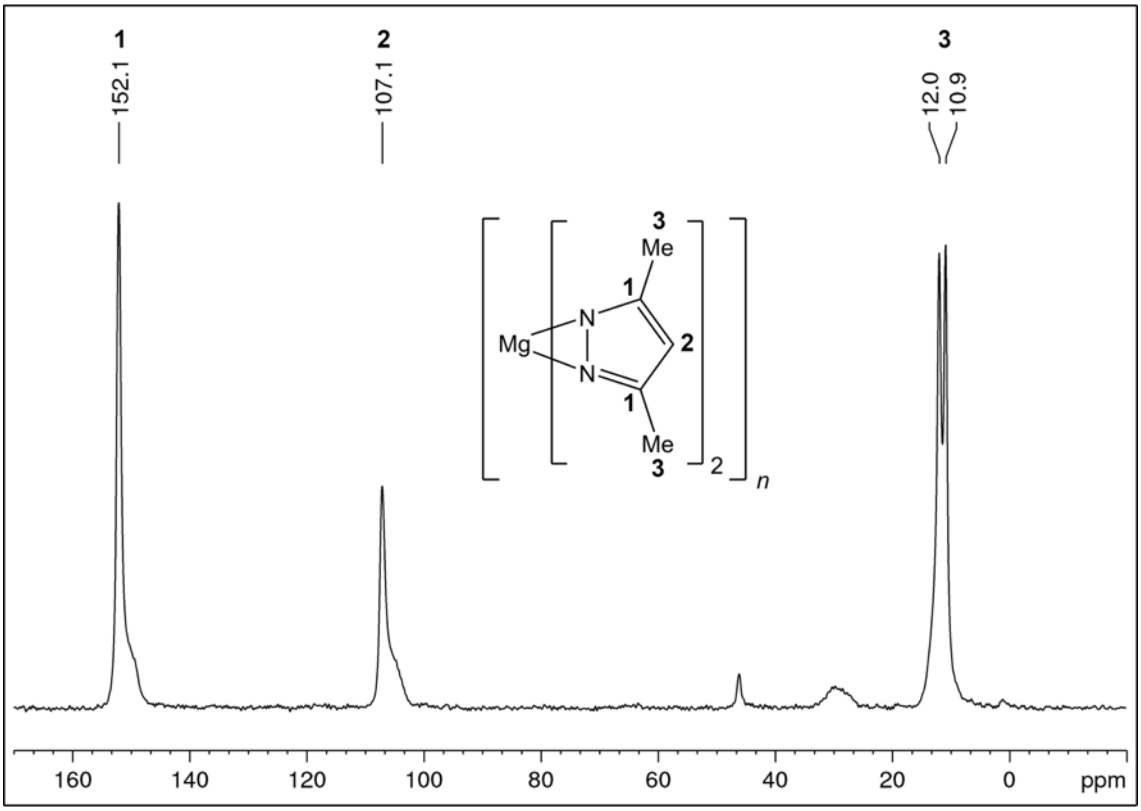

Figure S22. ^{13}C CP/MAS NMR spectrum (75.47 MHz, MAS at 8 kHz) of $[Mg(pz^{Me,Me})_2]_n$ (**7**).

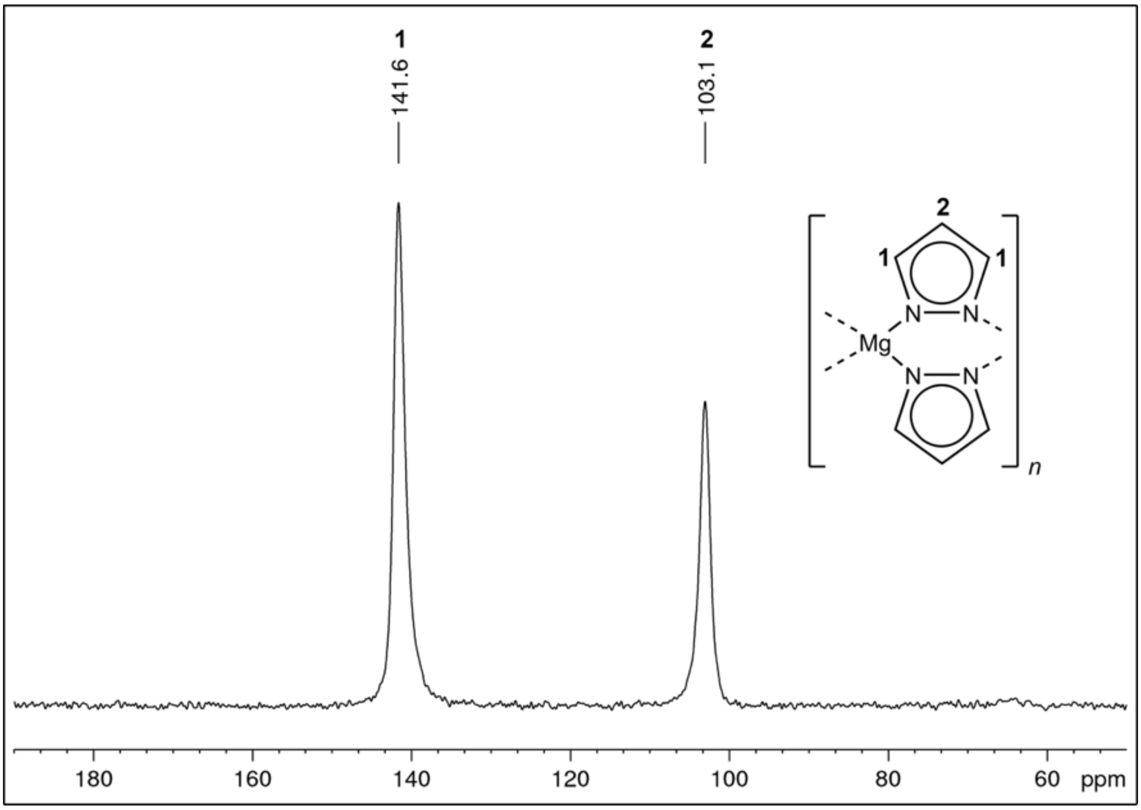

Figure S23. ^{13}C CP/MAS NMR spectrum (75.47 MHz, MAS at 8 kHz) of $[Mg(pz)_2]_n$ (**8**).

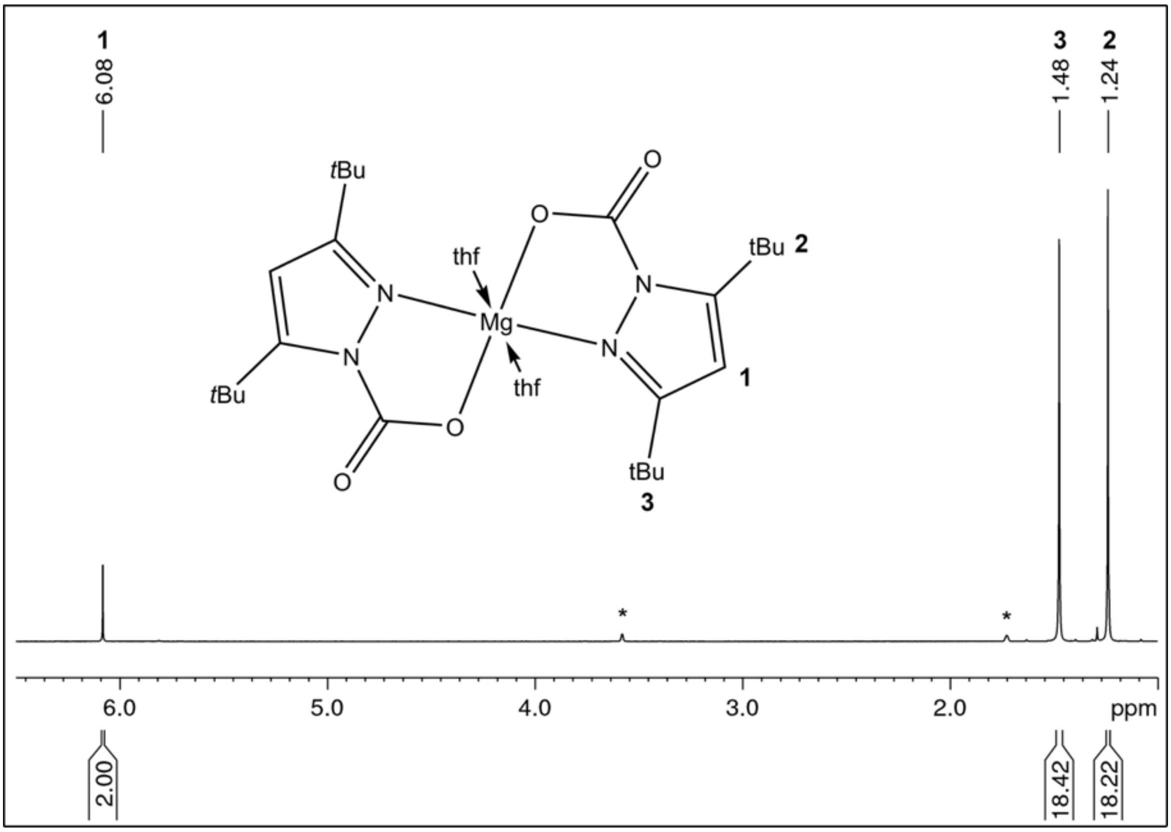

Figure S24. ^{1}H NMR spectrum (26 °C, 400.12 MHz, [D_8]THF) of [Mg(CO_2·pz^{tBu,tBu})$_2$(thf)$_2$] (**1-CO₂,thf**).

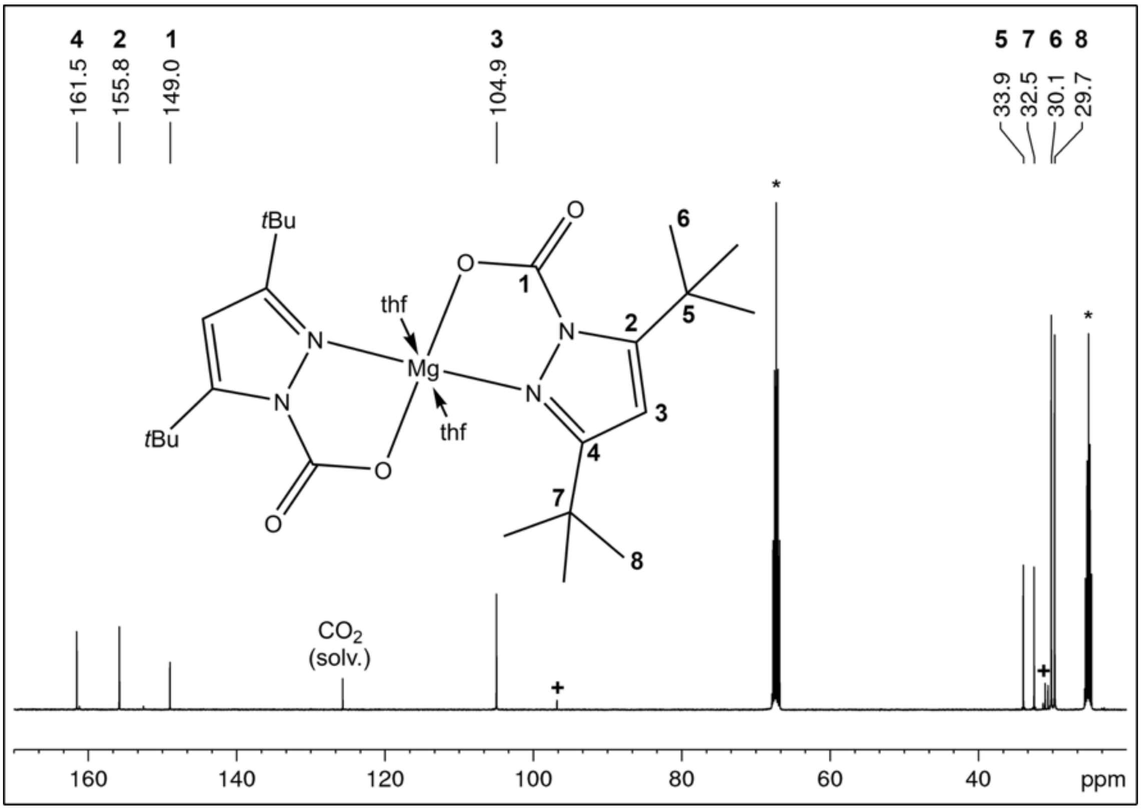

Figure S25. $^{13}C\{^{1}H\}$ NMR spectrum (26 °C, 100.60 MHz, [D_8]THF) of [Mg(CO_2·pz^{tBu,tBu})$_2$(thf)$_2$] (**1-CO₂,thf**) (+ impurities).

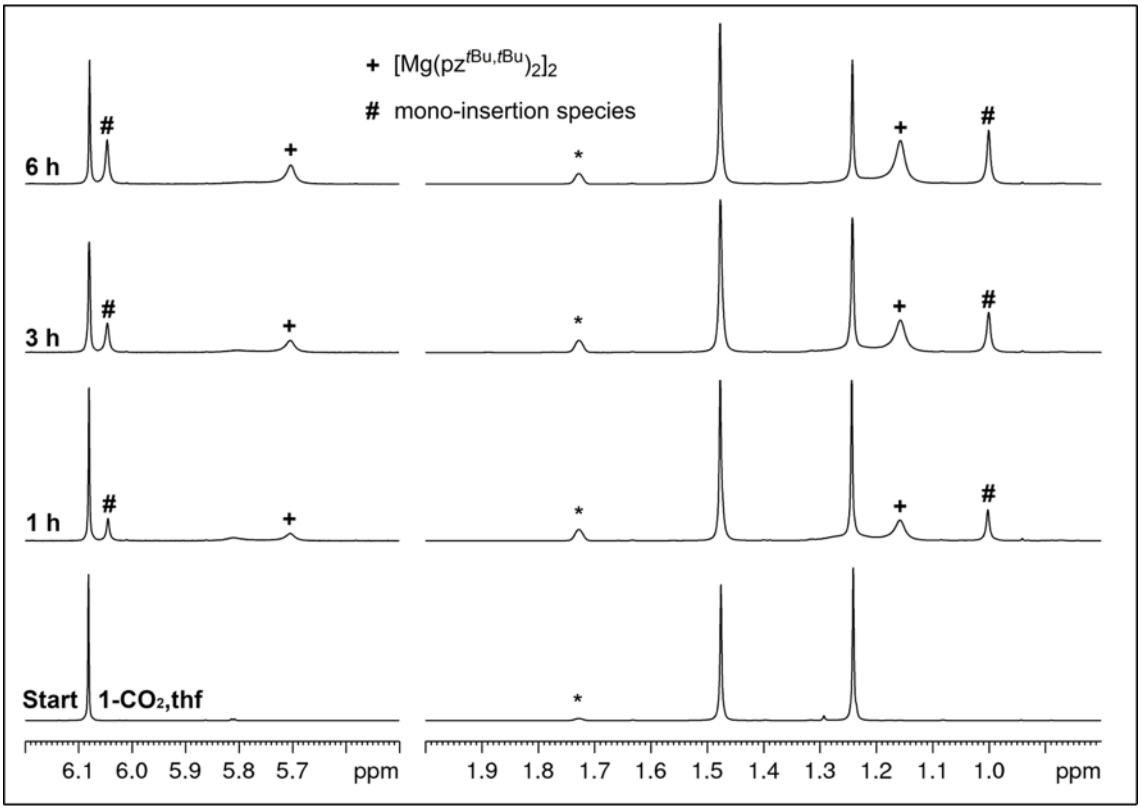

Figure S26. Comparison of ^{1}H NMR spectra (26 C, 400 MHz, [D$_8$]THF) of [Mg(CO$_2$·pz^{tBu,tBu})$_2$(thf)$_2$] (**1-CO$_2$,thf**) after exposure to reduced pressure over different time periods showing the formation of [Mg(pz^{tBu,tBu})$_2$(thf)]$_2$ (**1-thf**, +) and proposed [Mg(CO$_2$·pz^{tBu,tBu})(pz^{tBu,tBu})(thf)$_x$] (#).

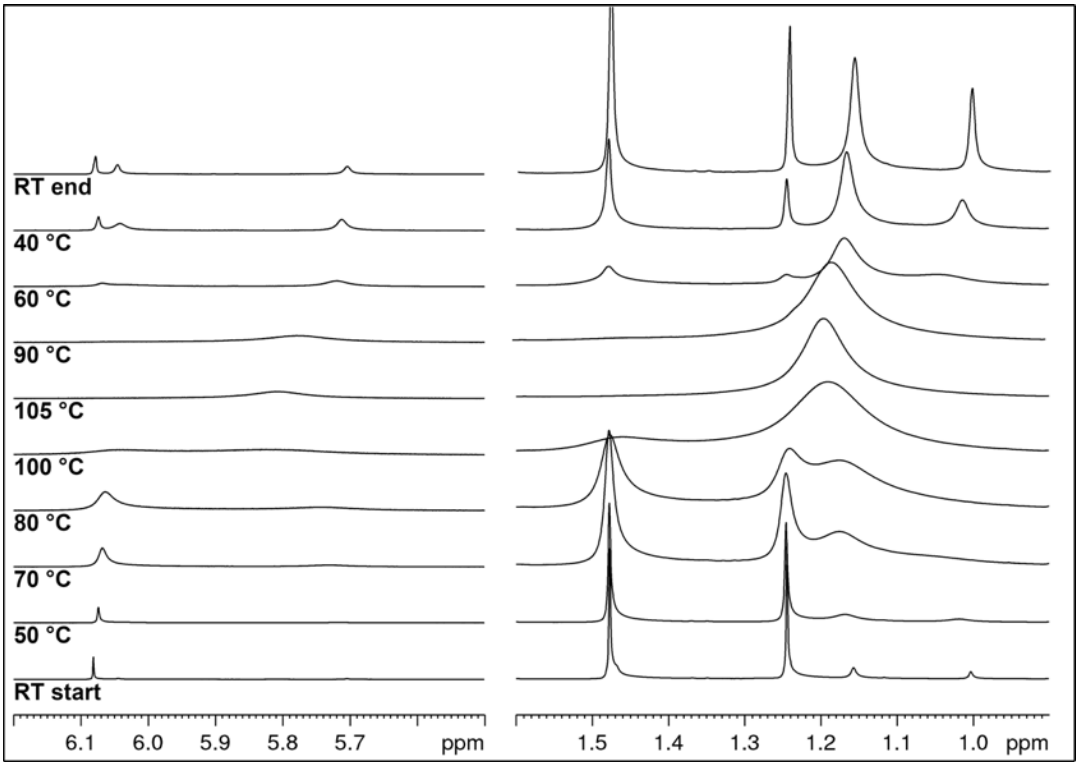

Figure S27. VT ^{1}H NMR spectra (500.13 MHz, [D$_8$]THF) of [Mg(CO$_2$·pz^{tBu,tBu})$_2$(thf)$_2$] (**1-CO$_2$,thf**) in the range of 26 °C to 105 °C showing the formation of [Mg(pz^{tBu,tBu})$_2$(thf)]$_2$ (**1-thf**) and proposed [Mg(CO$_2$·pz^{tBu,tBu})(pz^{tBu,tBu})(thf)$_x$].

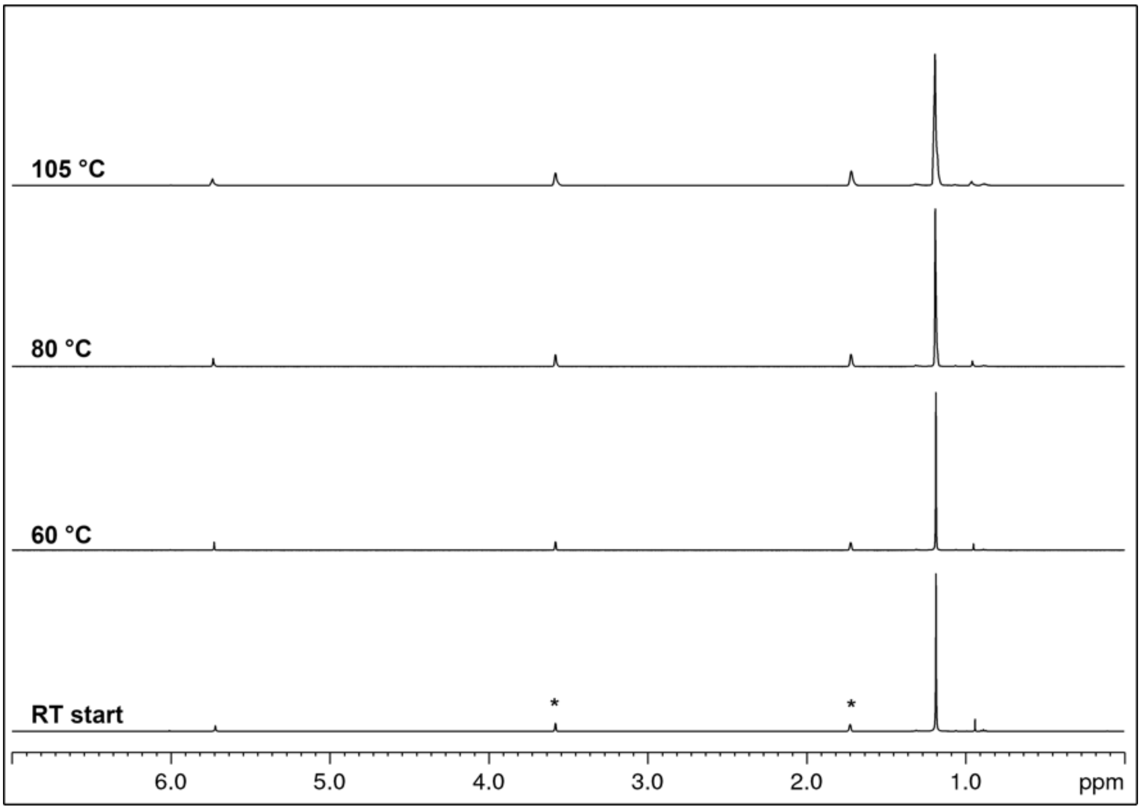

Figure S28. VT ^{1}H NMR spectra (500.13 MHz, [D_8]THF) of [Mg(pz^{tBu,tBu})$_2$(thf)$_2$] (**1-thf**) in the range of 26 °C to 105 °C.

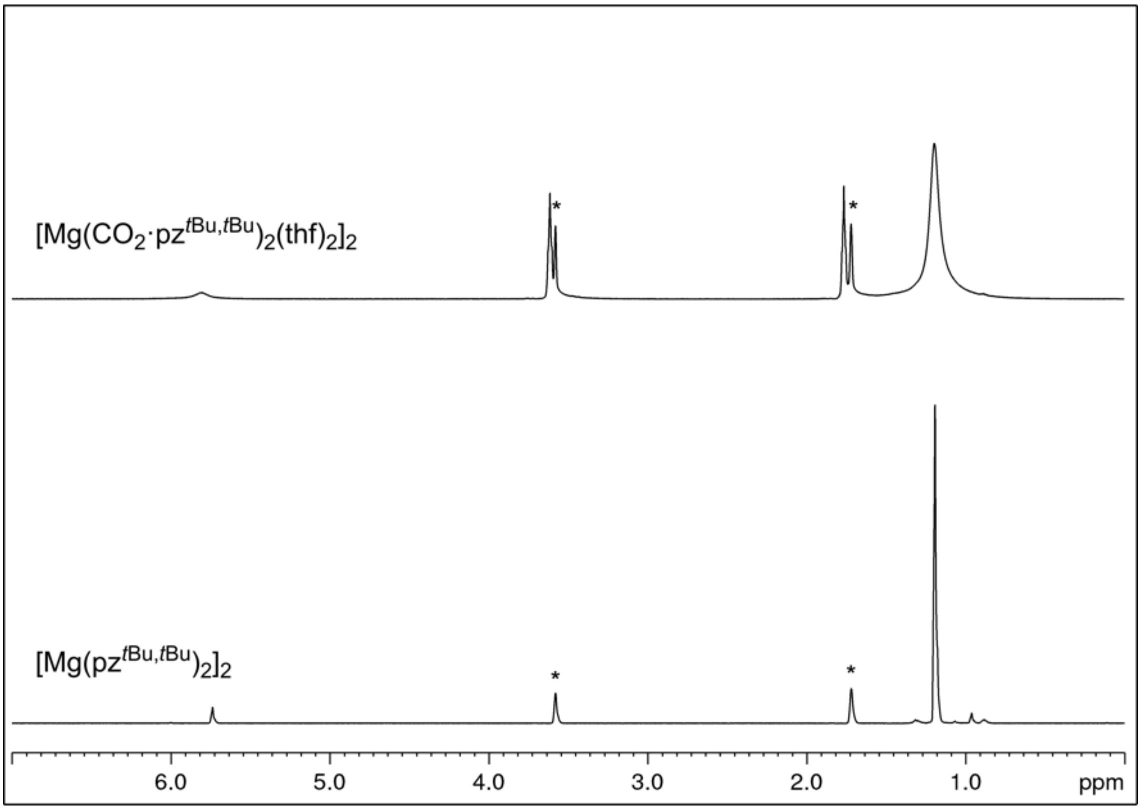

Figure S29. Comparison of ^{1}H NMR spectra (500.13 MHz, [D_8]THF) of [Mg(pz^{tBu,tBu})$_2$(thf)]$_2$ (**1-thf**) and [Mg(CO_2·pz^{tBu,tBu})$_2$(thf)$_2$] (**1-CO$_2$,thf**) at 105 °C.

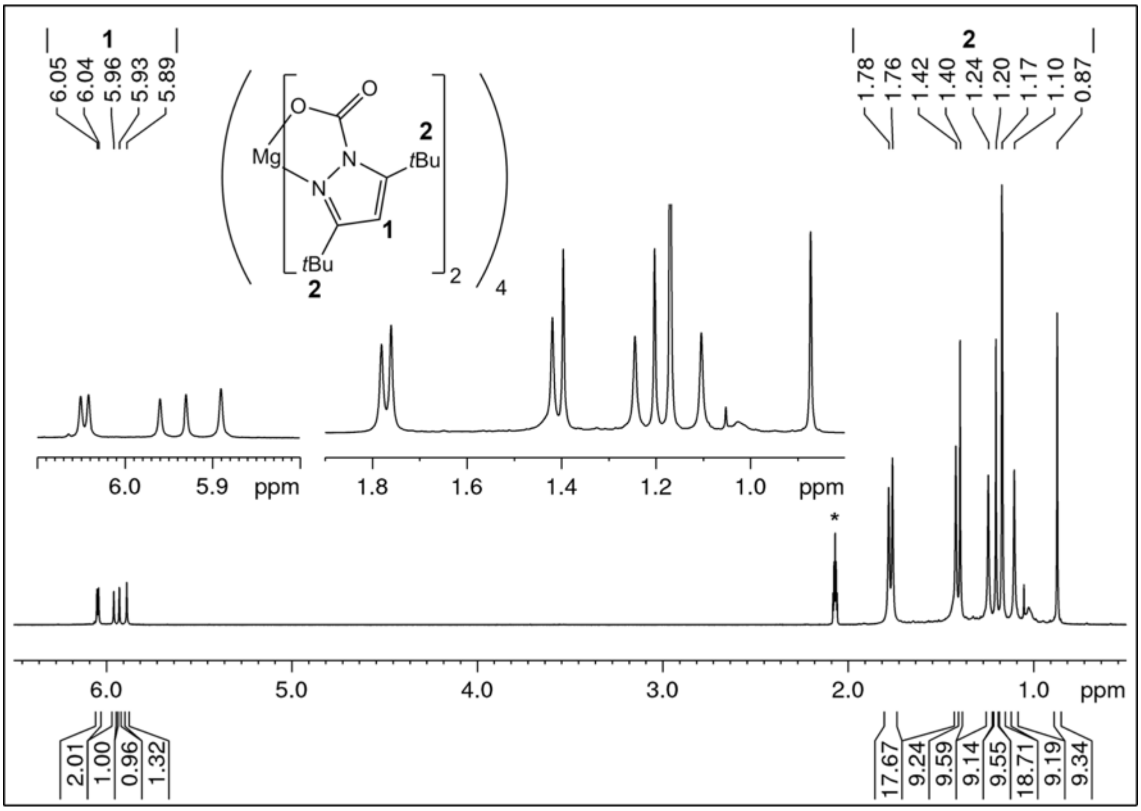

Figure S30. ^{1}H NMR spectrum (26 °C, 400.11 MHz, [D$_8$]toluene) of [Mg(CO_2·pz^{tBu,tBu})$_2$]$_4$ (**1-CO$_2$**).

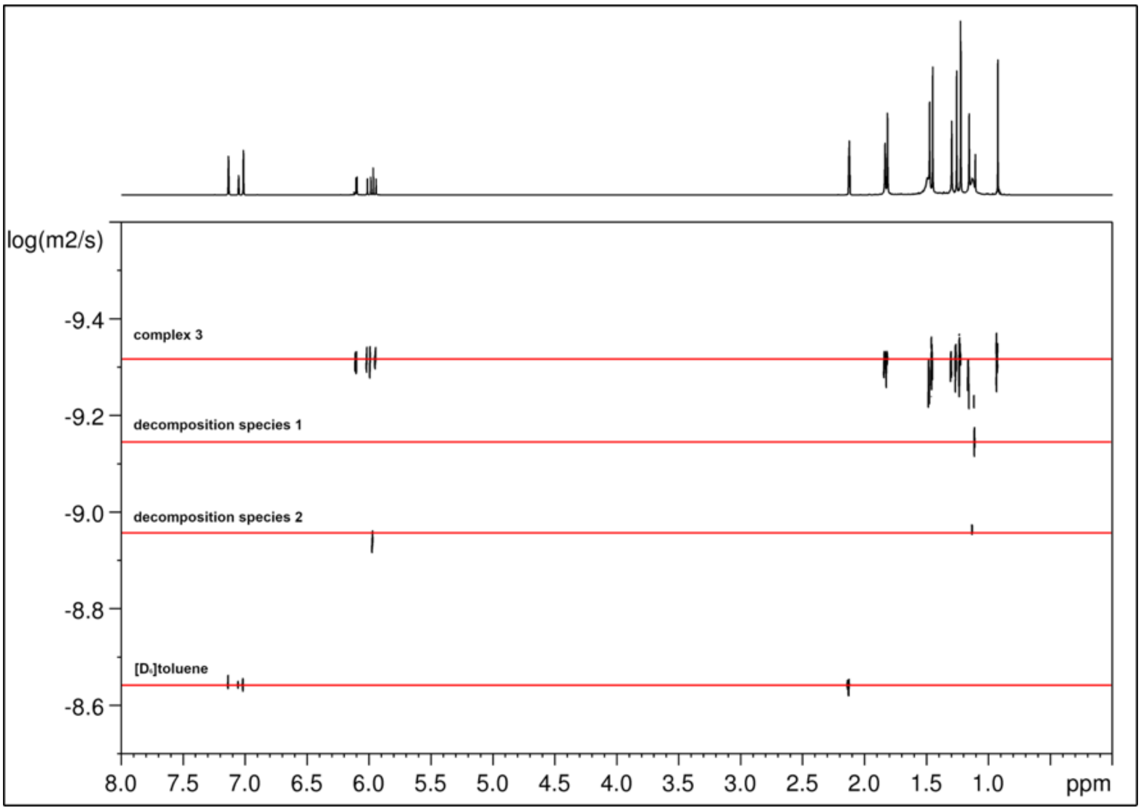

Figure S31. ^{1}H DOSY NMR spectrum (26 °C, 700.29 MHz, [D$_8$]toluene) of [Mg(CO_2·pz^{tBu,tBu})$_2$]$_4$ (**1-CO$_2$**). Decomposition species 2 is most likely HO_2Cpz^{tBu,tBu} but a molecular weight calculation was not possible due to overlapping of the signals with **1-CO$_2$**. Decomposition species 1 could not be assigned to a specific product.

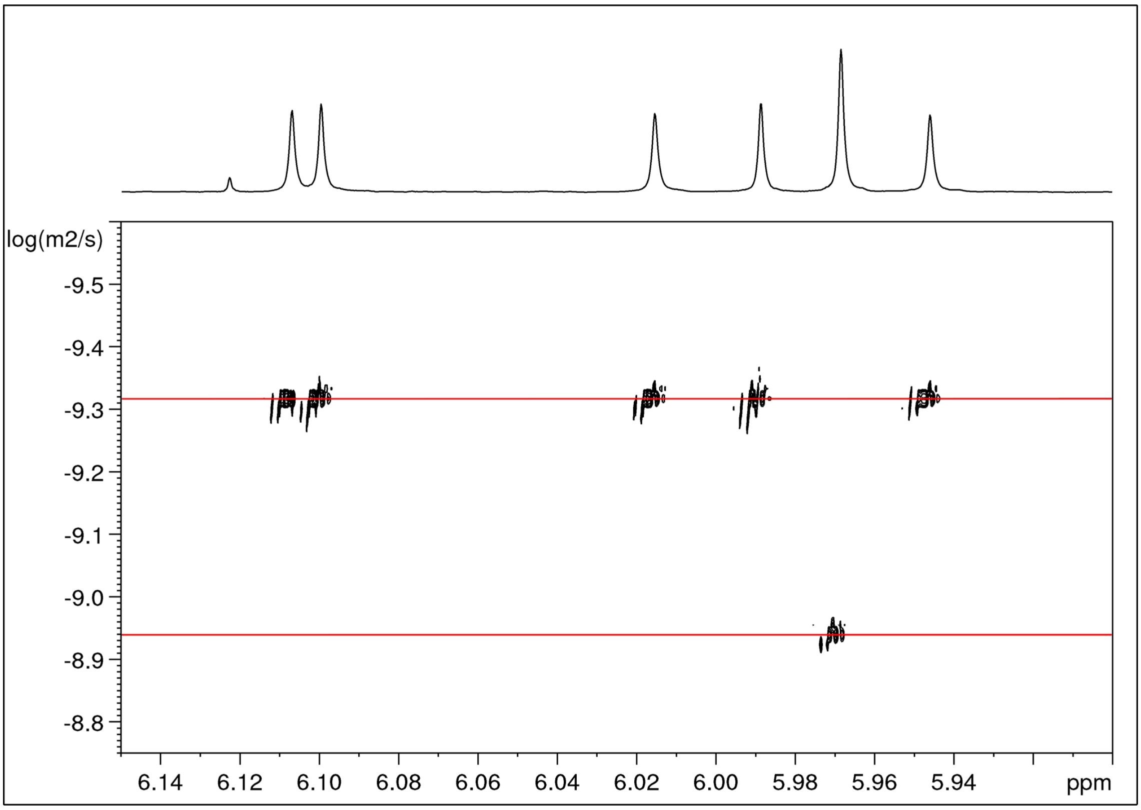

Figure S32. ^{1}H DOSY NMR spectrum (26 °C, 700.29 MHz, [D$_8$]toluene) of [Mg(CO$_2$·pz^{tBu,tBu})$_2$]$_4$ (**1-CO$_2$**, top red line) and decomposition product 2 (bottom red line) in the aromatic region.

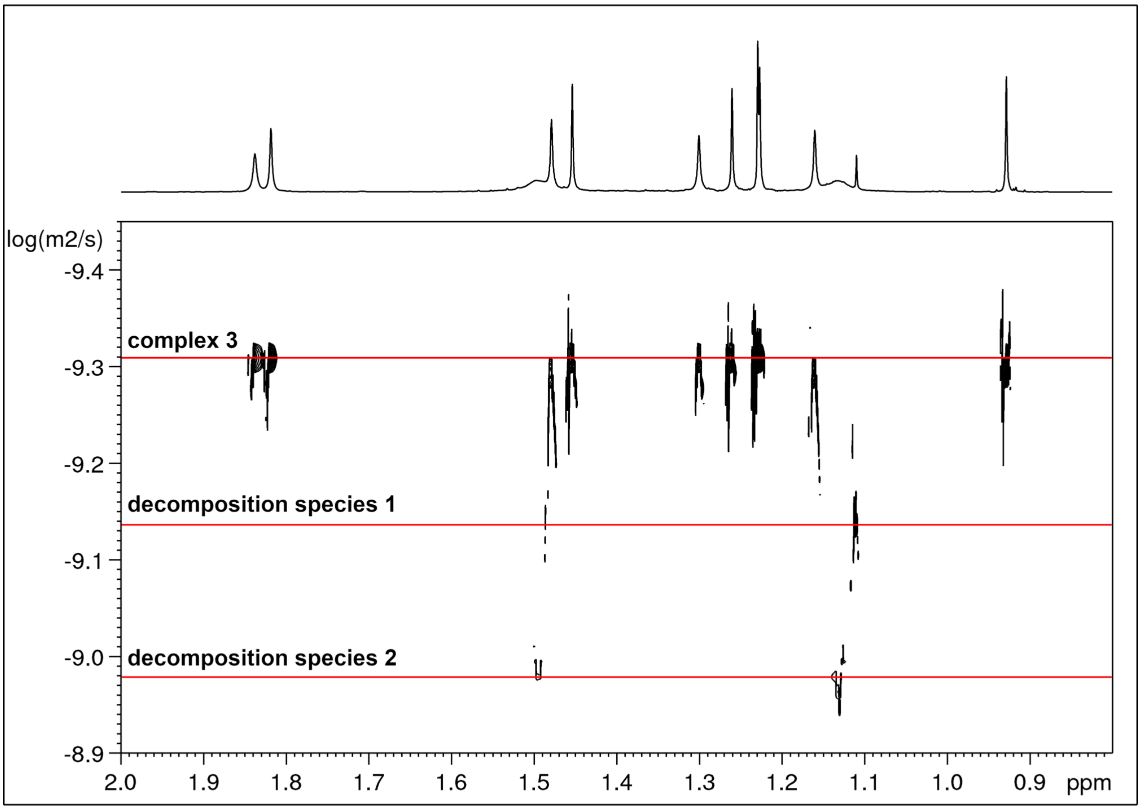

Figure S33. ^{1}H DOSY NMR spectrum (26 °C, 700.29 MHz, [D$_8$]toluene) of [Mg(CO$_2$·pz^{tBu,tBu})$_2$]$_4$ (**1-CO$_2$**) in the region of *t*Bu moities. Abbrivations of the signals of **1-CO$_2$** at 1.48 and 1.16 ppm of the diffusion coefficient of **1-CO$_2$** are due to overlapping with signals of decomposition product 2.

^{1}H DOSY NMR Experiment Data of [Mg(CO_2·pz^{tBu,tBu})$_2$]$_4$ (1-CO_2)

The ^{1}H DOSY NMR measurement was performed to determine whether **1-CO₂** is an oligomeric species as indicated by the ^{1}H NMR spectrum. Indeed, the DOSY experiment showed only one species with one diffusion coefficient for all 15 signals of **1-CO_2**. In the period between recording the initial ^{1}H NMR spectrum and performing the DOSY experiment two decomposition products formed which are marked in Figures S27-S29. Each of these decomposition products has a specific diffusion coefficient. According to its chemical shift, decomposition product 2 is most likely the carbamic acid HO_2Cpz^{tBu,tBu}. However, a molecular weight calculation was not possible, due to overlapping of the signals with other species.

Table S4. Diffusion coefficients in [D$_6$]toluene determined via ^{1}H DOSY NMR experiment

Evaluation	D (average)	Variance	Variance%	pD (average)
T1T2	1.0323E-09	9.393E-11	9.099E-02	-898.620%
Contin	1.171E-09	1.335E-12	1.140E-03	-893.145%
Expt	1.0955E-09	6.816E-11	6.222E-02	-896.039%

The diffusion coefficients (D) where determined via three different evaluation methods provided by Topspin. The T1/T2 module uses a numeric calculation for D, whereas the contin/exponential evaluation uses manual signal picking. D was calculated from the measured pD values. The D values listed in Table S4 are averaged overall signals assigned to one species that are not overlapping with signals from another species.

Table S5. Molecular weight calculation via the measured diffusion coefficients

	Shpere			Ellipsoid			Merged		
Evaluation	M(calc.)	M(found)	Deviation	M(calc.)	M(found)	Deviation	M (calc.)	M(found)	Deviation
T1T2	1883.59	1721	9%	1280.66	1133	12%	1280.66	1104	14%
Contin	1883.59	1911	-1%	1280.66	1234	4%	1280.66	1200	6%
Expt	1883.59	1875	0%	1280.66	1215	5%	1280.66	1182	8%

For the molecular weight calculation the method described by Stalke was used.[11] The approximation of a highly compacted sphere is probably the best way to describe such a oligomer due to the molecular rotation and vibration. Using this approximation, molecular weights between 1721 and 1911 g/mol were found applying the three evaluation methods. This fits good to the tetrameric species [Mg(CO_2·pz^{tBu,tBu})$_2$]$_4$ with a molecular weight of 1883.59 g/mol. All three evaluation methods are within the methods error of 9 % with the exponential evaluation being the closet with 1875 g/mol. Changing the approximation from a sphere to an ellipsoid or a less accurately merged one, the deviation from an oligomer of the form [Mg(CO_2·pz^{tBu,tBu})$_2$]$_n$ is too big for an accordance. The found molecular weights are between 1104 and 1234 g/mol, which does not fit for any oligomeric species with complete CO_2 insertion. However, given that a highly compact sphere is the most likely state in solution over the NMR timescale and the solid-state experiments speaks for a complete CO_2 insertion, **1-CO_2** is most likely the tetrameric species [Mg(CO_2·pz^{tBu,tBu})$_2$]$_4$.

Since a crystal structure of **1-CO_2** could not be obtained, a comparison of the calculated and measured hydrodynamic radii was not possible.

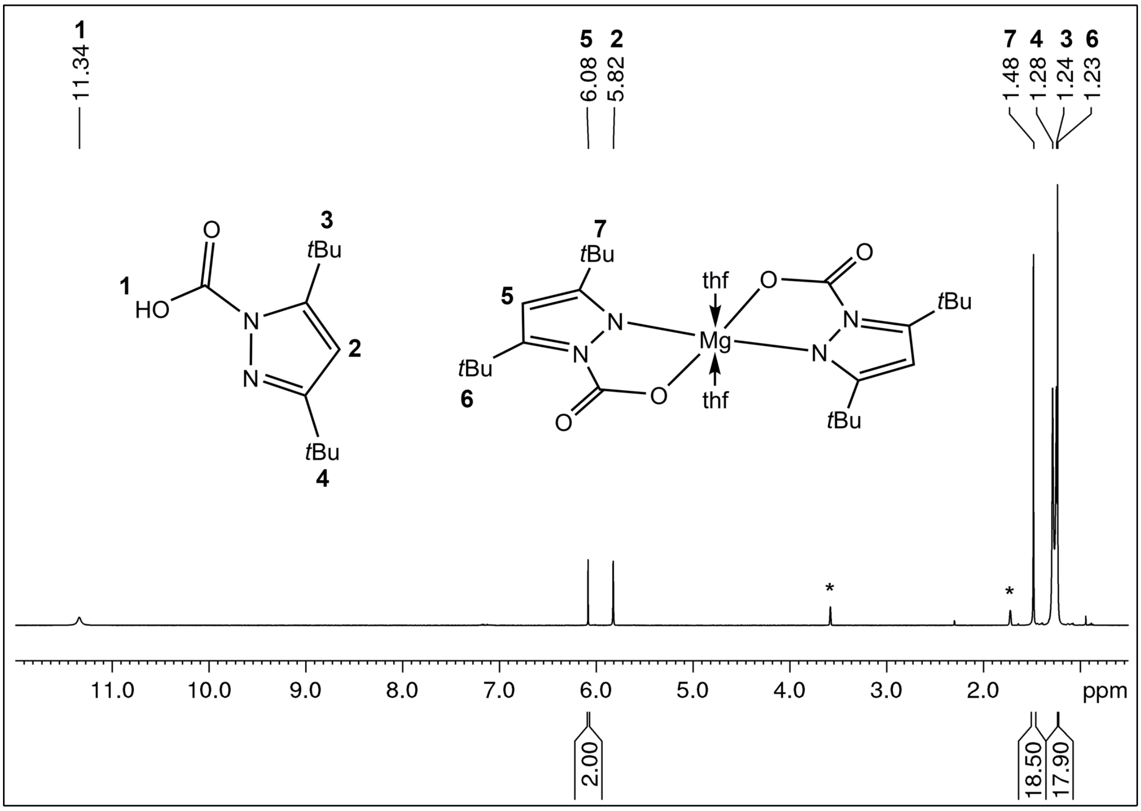

Figure S34. ^{1}H NMR spectrum (26 °C, 500.13 MHz, [D$_8$]THF) of the reaction of [Mg(pz^{tBu,tBu})$_2$(Hpz^{tBu,tBu})$_2$] (**1-Hpz**) with CO_2 and the formation of [Mg(CO_2·pz^{tBu,tBu})$_2$(thf)$_2$] (**1-CO$_2$,thf**) and HO$_2$Cpz^{tBu,tBu}.

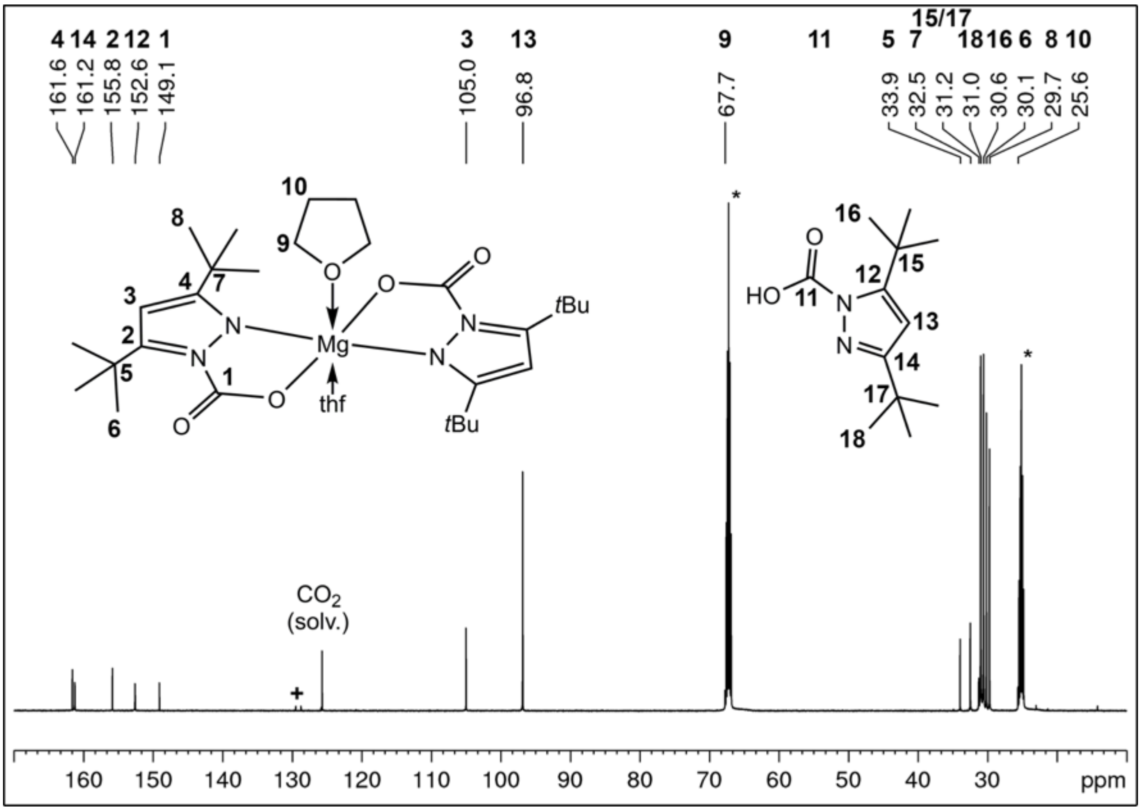

Figure S35. $^{13}C\{^{1}H\}$ NMR spectrum (26 °C, 125.76 MHz, [D$_8$]THF) of the reaction of [Mg(pz^{tBu,tBu})$_2$(Hpz^{tBu,tBu})$_2$] (**1-Hpz**) with CO_2 and the formation of [Mg(CO_2·pz^{tBu,tBu})$_2$(thf)$_2$] (**1-CO$_2$,thf**) and HO$_2$Cpz^{tBu,tBu} (**+** impurities).

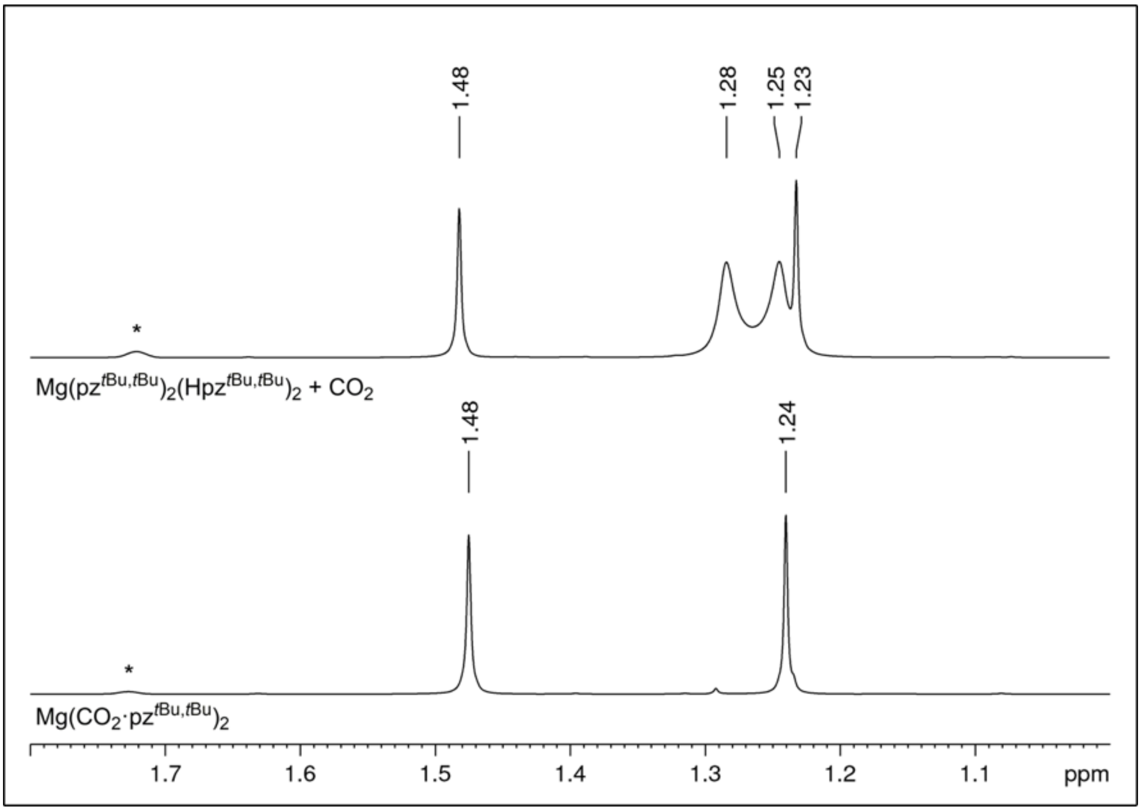

Figure S36. Comparison of the ^{1}H NMR spectra (26 °C, 400.11 MHz, [D$_8$]THF) of the reaction of [Mg(pz^{tBu,tBu})$_2$]$_2$ (**1**, bottom) and [Mg(pz^{tBu,tBu})$_2$(Hpz^{tBu,tBu})$_2$] (**1-Hpz**, top) with CO_2 in [D$_8$]THF.

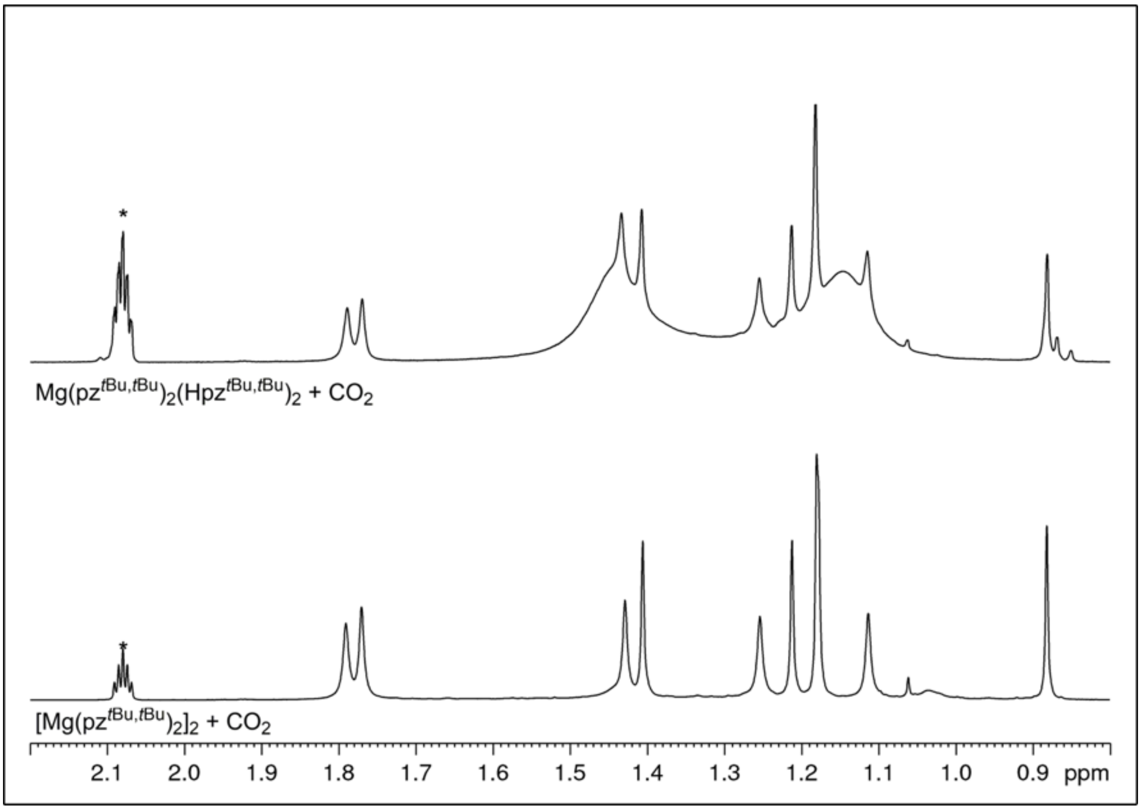

Figure S37. Comparison of the ^{1}H NMR spectra (26 °C, 400.11 MHz, [D$_8$]toluene) of the reaction of [Mg(pz^{tBu,tBu})$_2$]$_2$ (**1**, bottom) and [Mg(pz^{tBu,tBu})$_2$(Hpz^{tBu,tBu})$_2$] (**1-Hpz**, top) with CO_2 in [D$_8$]toluene.

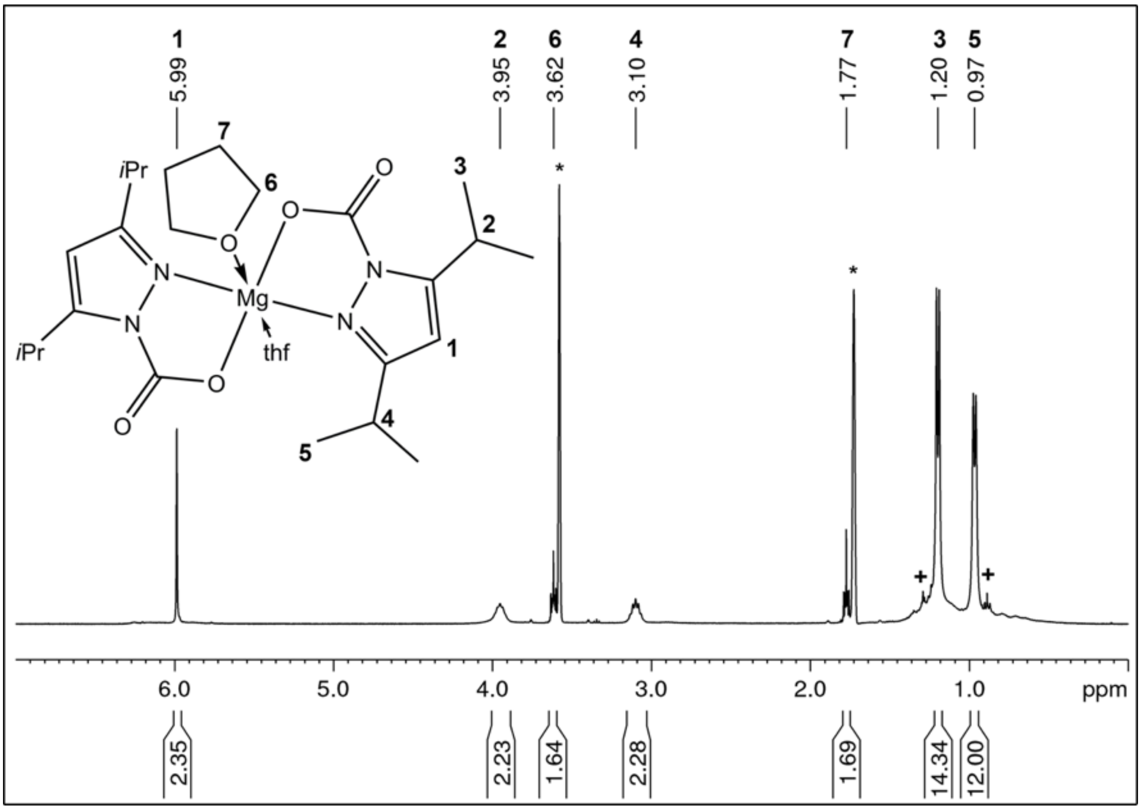

Figure S38. ^{1}H NMR spectrum (26 °C, 400.11 MHz, [D$_8$]THF) of [Mg(CO$_2$·pz^{iPr,iPr})$_2$(thf)$_2$] (**4-CO$_2$,thf**) (**+** *n*-hexane).

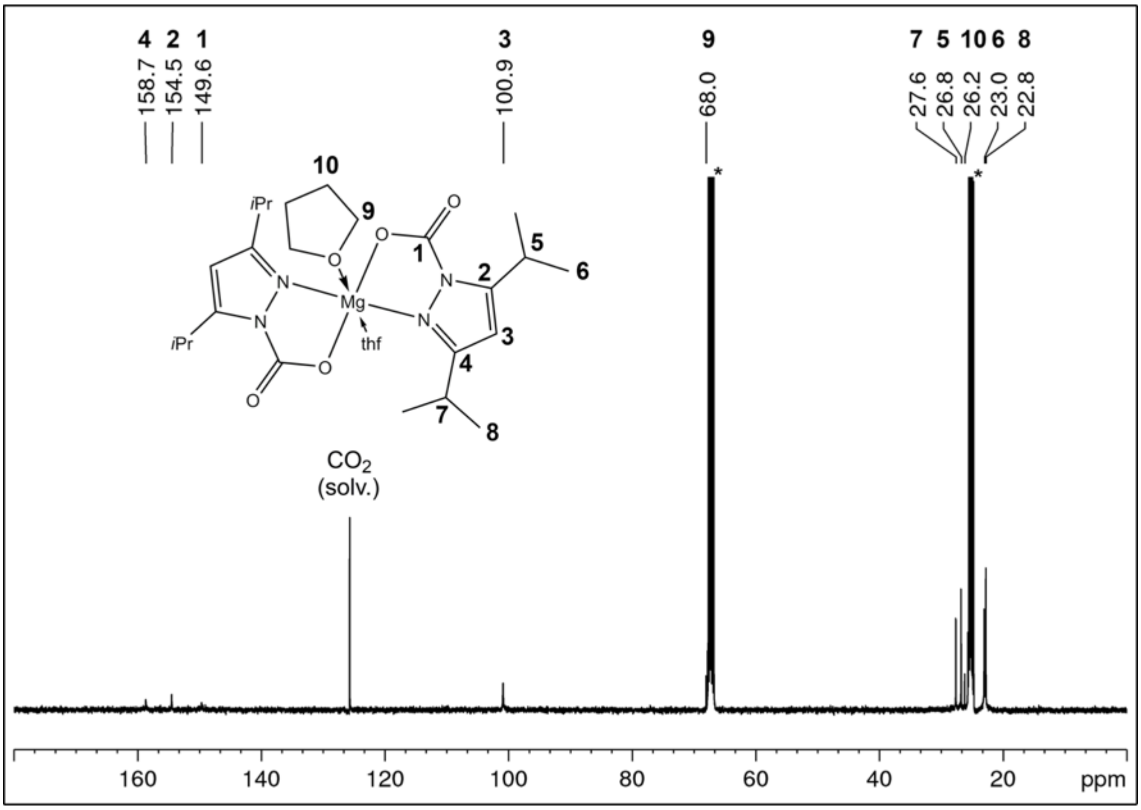

Figure S39. ^{13}C{^{1}H} NMR spectrum (26 °C, 100.61 MHz, [D$_8$]THF) of [Mg(CO$_2$·pz^{iPr,iPr})$_2$(thf)$_2$] (**4-CO$_2$,thf**).

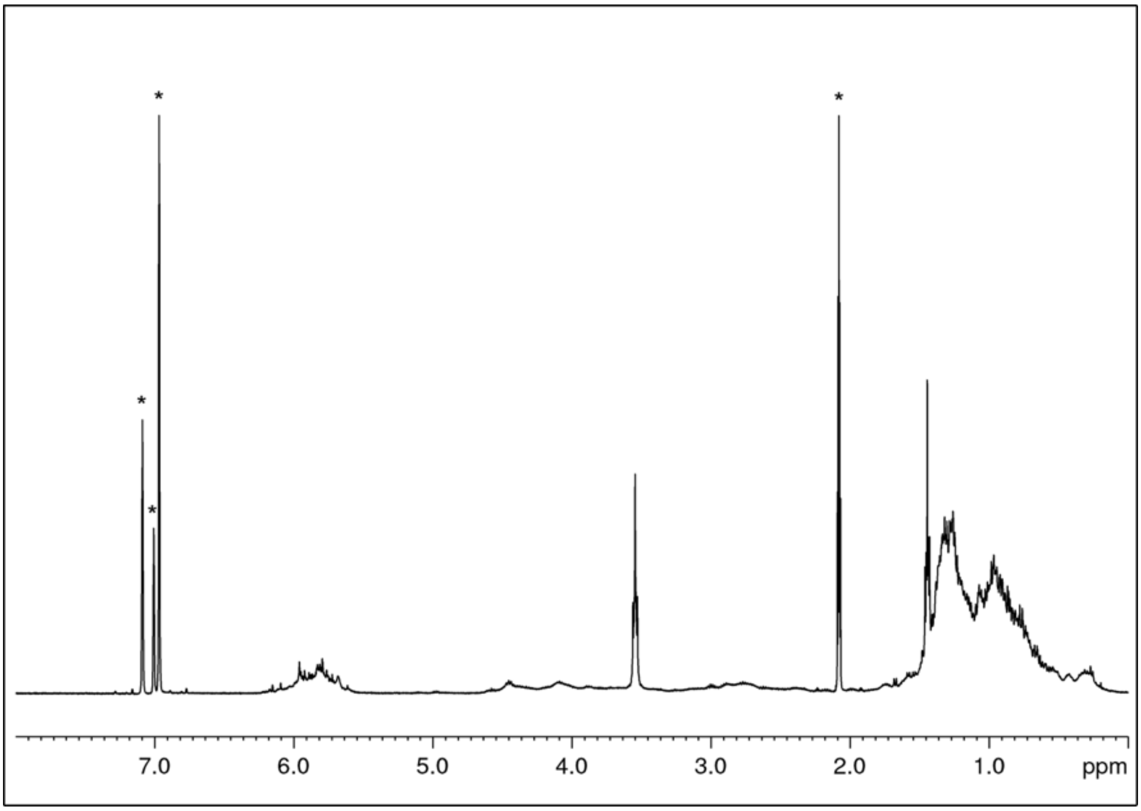

Figure S40. ^{1}H NMR spectrum (26 °C, 400.11 MHz, [D$_8$]toluene) of [Mg(CO_2·pz^{iPr,iPr})$_2$]$_4$ (**4-CO₂**).

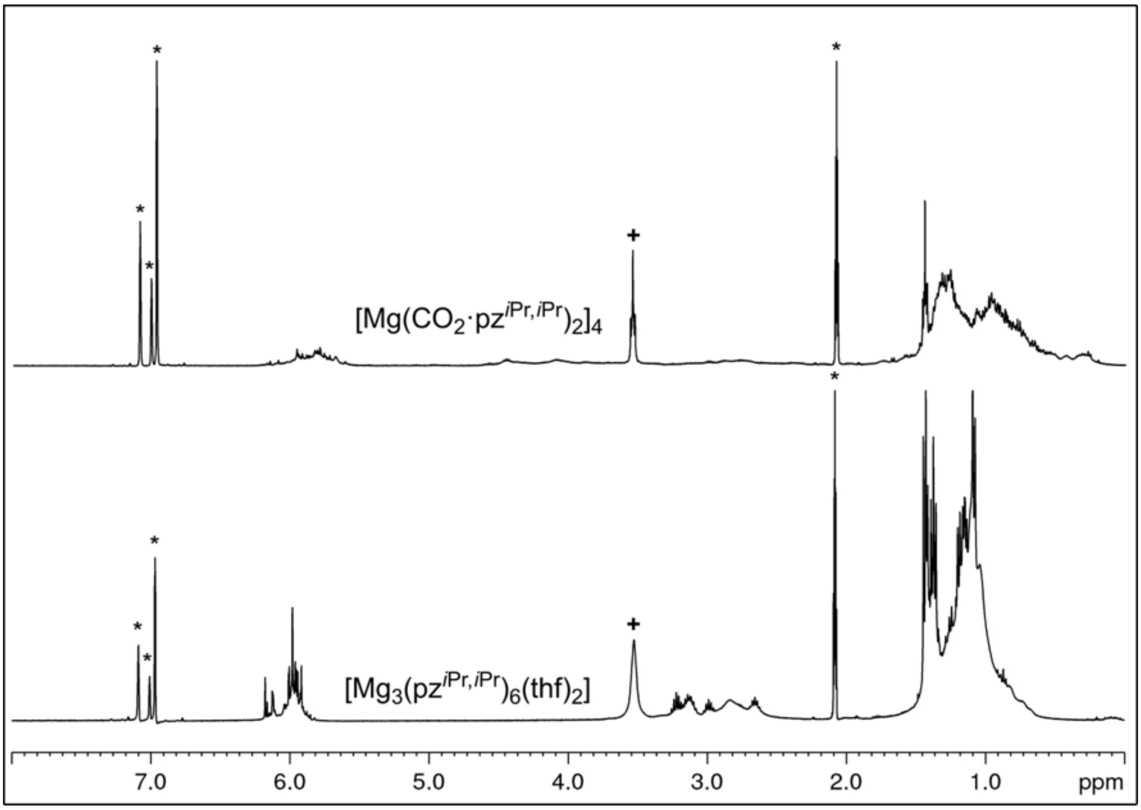

Figure S41. Comparison of the ^{1}H NMR spectra (26 °C, 100.61 MHz, [D$_8$]toluene) of [Mg$_3$(pz^{iPr,iPr})$_6$(thf)$_2$] (**4-thf**, bottom) and [Mg(CO_2·pz^{iPr,iPr})$_2$]$_4$ (**4-CO$_2$**, top) (**+** residual THF).

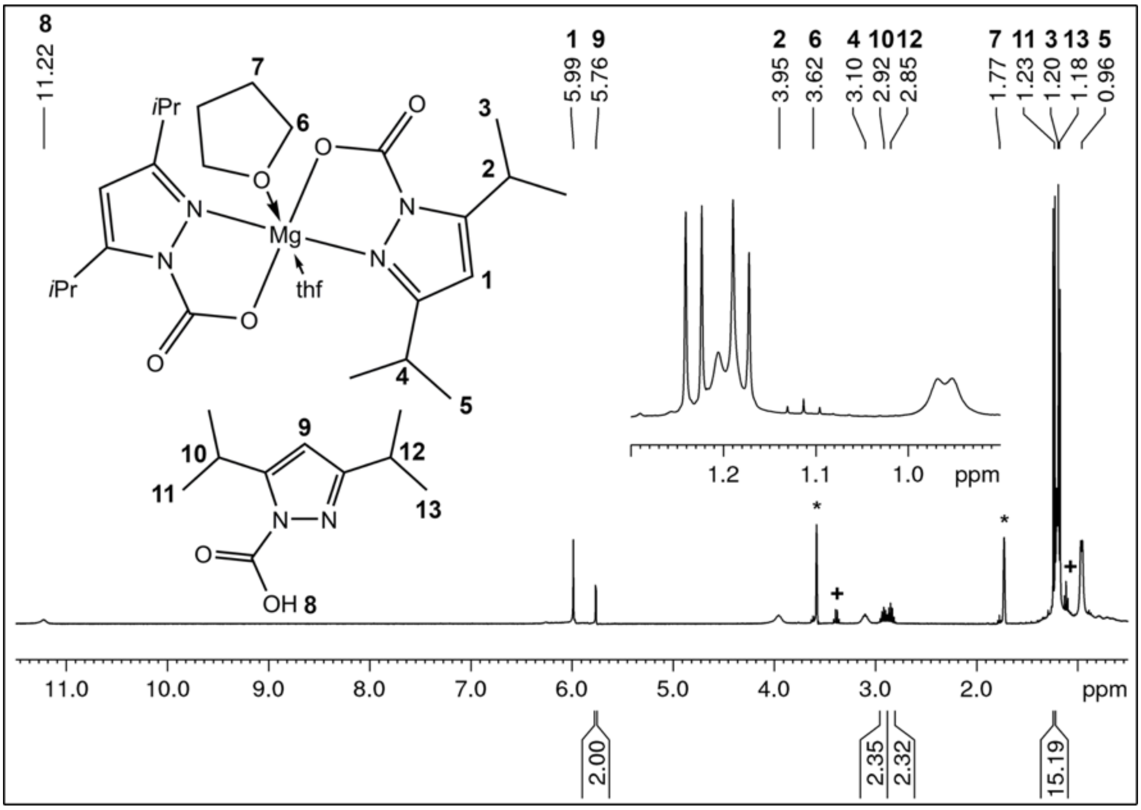

Figure S42. ^{1}H NMR spectrum (26 °C, 400.11 MHz, [D$_8$]THF) of the reaction of [Mg$_3$(pz^{iPr,iPr})$_6$(Hpz^{iPr,iPr})$_2$] (**4a-Hpz**) with CO_2 and the formation of [Mg(CO_2·pz^{iPr,iPr})$_2$(thf)$_2$] (**4-CO$_2$**) and HO$_2$Cpz^{iPr,iPr} (**+** Et$_2$O).

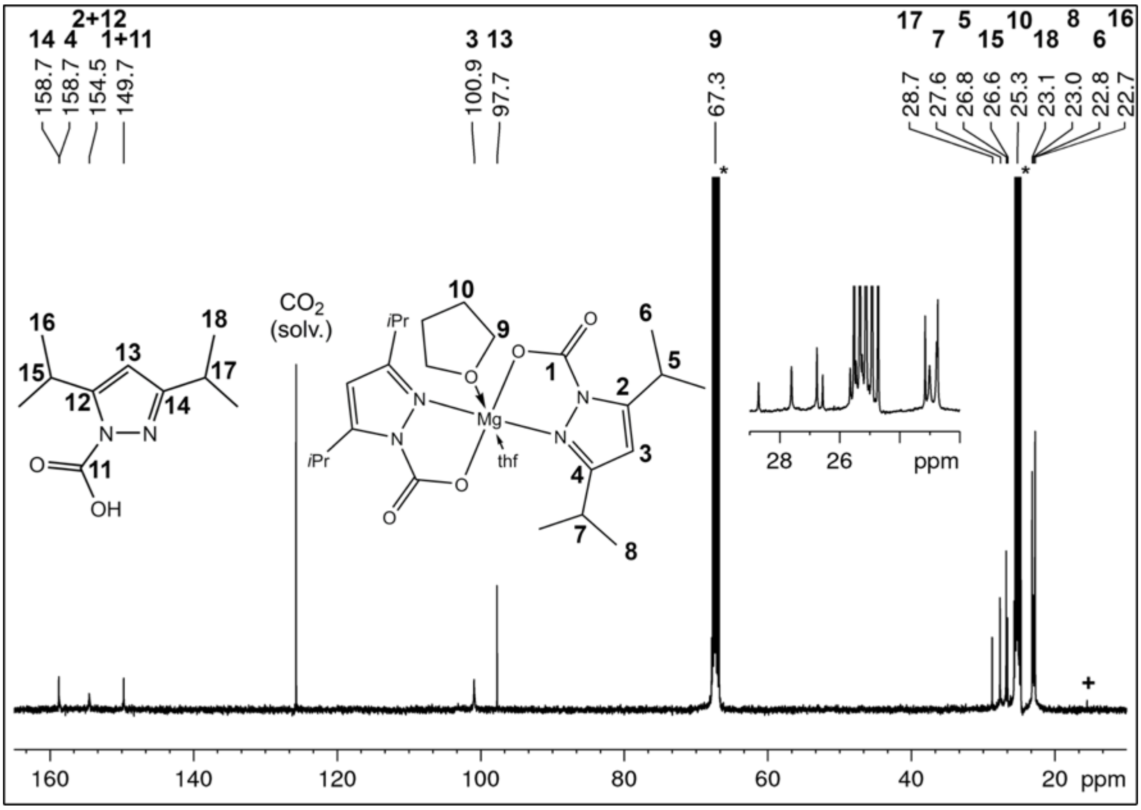

Figure S43. ^{13}C{^{1}H} NMR spectrum (26 °C, 100.61 MHz, [D$_8$]THF) of the reaction of [Mg$_3$(pz^{iPr,iPr})$_6$(Hpz^{iPr,iPr})$_2$] (**4a-Hpz**) with CO_2 and the formation of [Mg(CO_2·pz^{iPr,iPr})$_2$(thf)$_2$] (**4-CO$_2$**) and HO$_2$Cpz^{iPr,iPr} (**+** Et$_2$O).

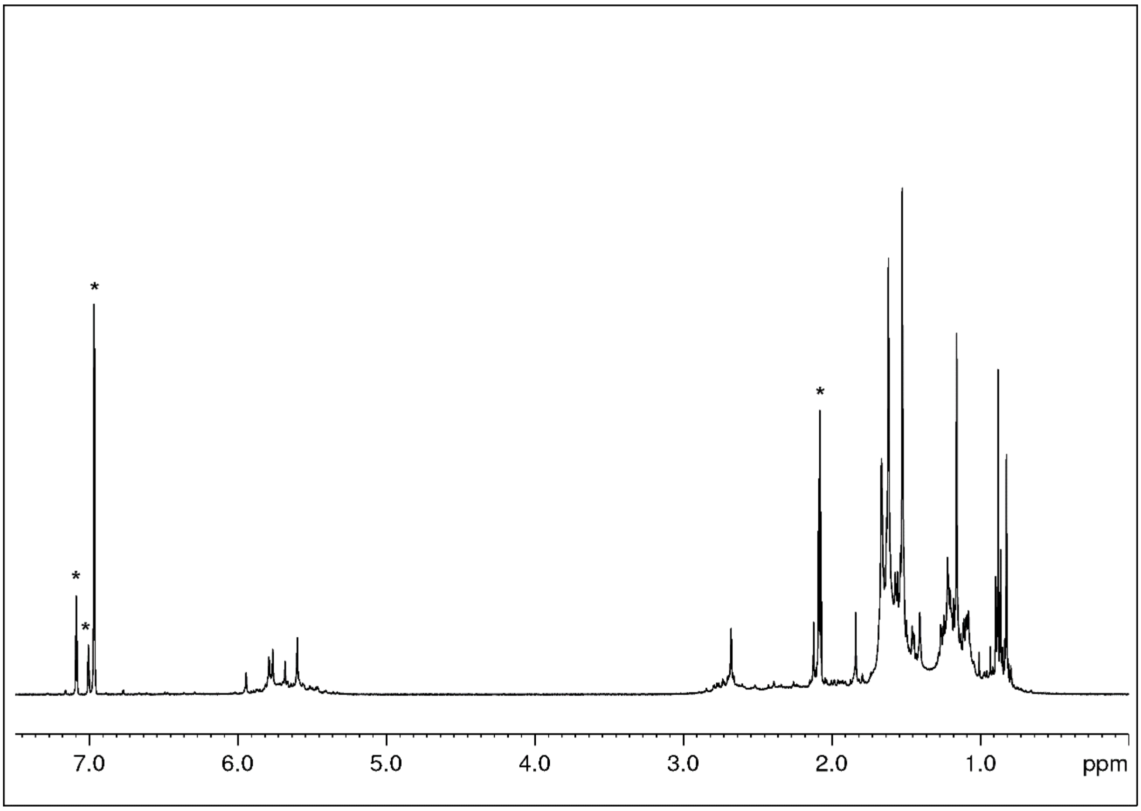

Figure S44. ^{1}H NMR spectrum (26 °C, 400.11 MHz, [D$_8$]toluene) of the reaction of [KMg(pz^{tBu,Me})$_3$]$_2$ (**2**) with CO_2.

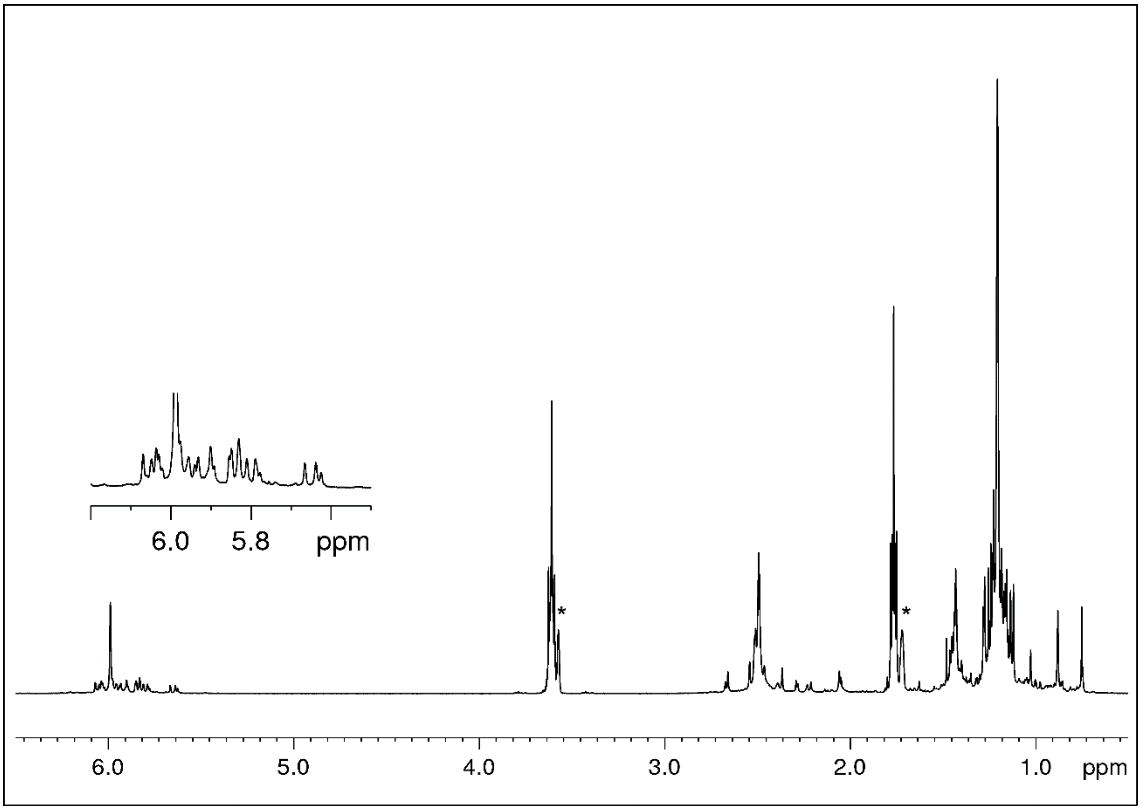

Figure S45. ^{1}H NMR spectrum (26 °C, 400.11 MHz, [D$_8$]THF) of the reaction of [Mg(pz^{tBu,Me})$_2$(thf)]$_2$ (**3**) with CO_2.

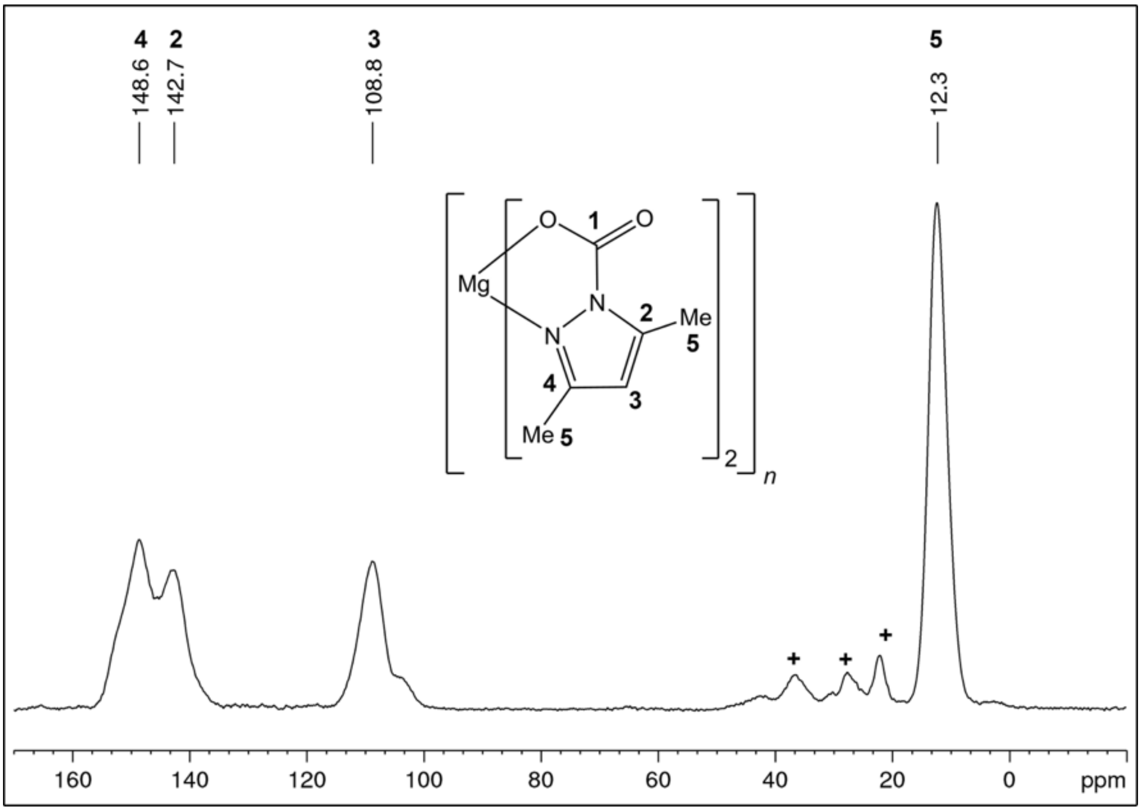

Figure S46. ^{13}C CP/MAS NMR spectrum (75.47 MHz, MAS at 8 kHz) of $[Mg(CO_2{\cdot}pz^{Me,Me})_2]_n$ (**7-CO₂**) (**+** *n*-hexane).

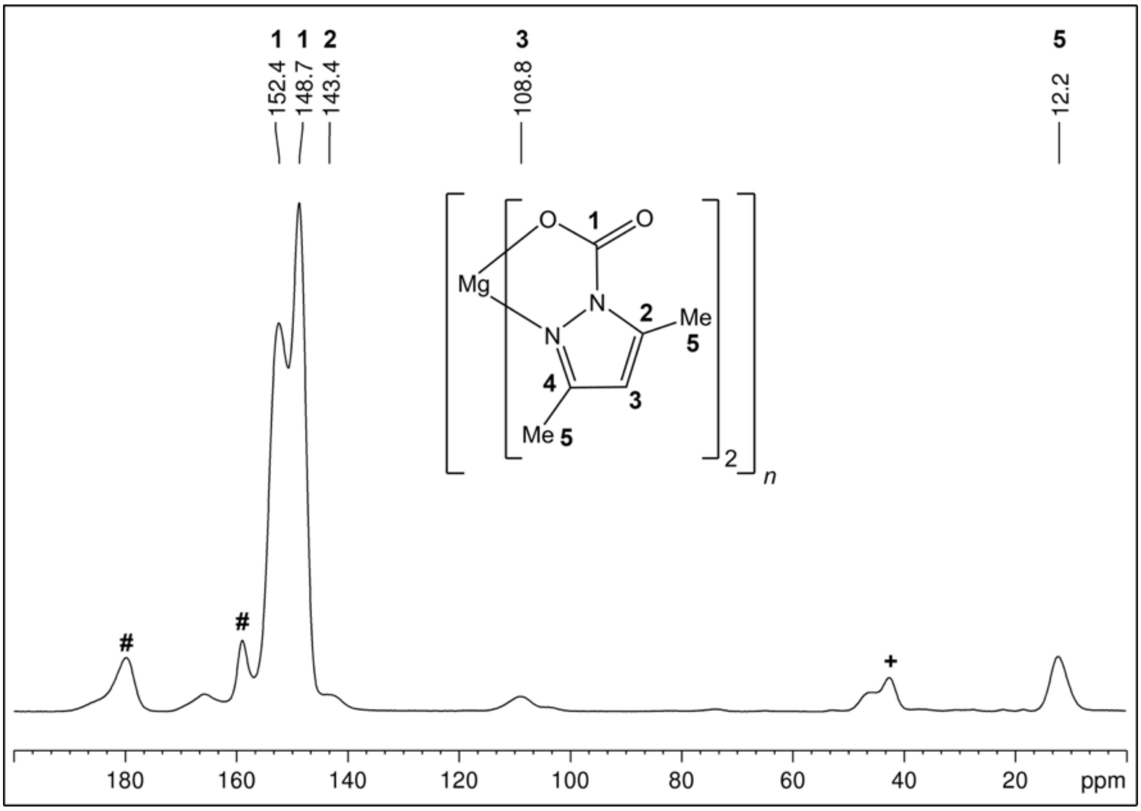

Figure S47. ^{13}C CP/MAS NMR spectrum (75.47 MHz, MAS at 8 kHz) of $[Mg(^{13}CO_2{\cdot}pz^{Me,Me})_2]_n$ (**7-CO₂**) (**+** rotation side band; **#** $^{13}CO_2$ unknown side product).

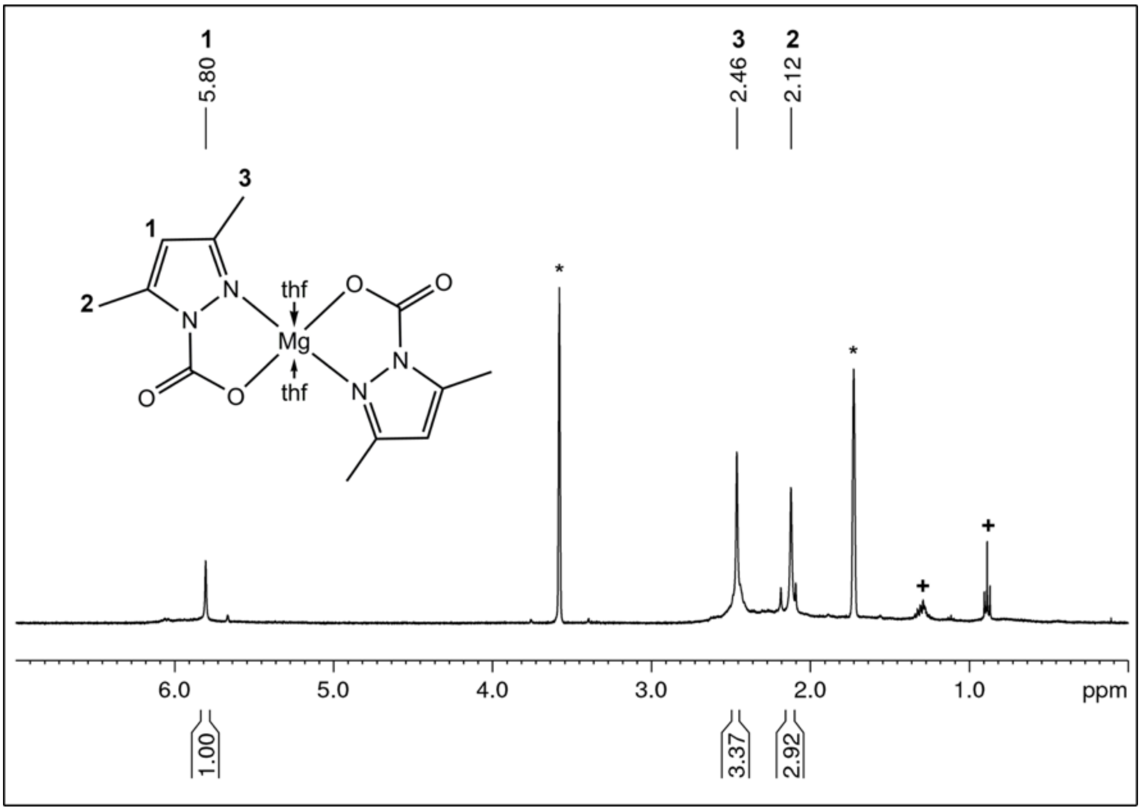

Figure S48. ^{1}H NMR spectrum (26 °C, 400.11 MHz, [D$_8$]THF) of $[Mg(CO_2{\cdot}pz^{Me,Me})_2]_n$ (**7-CO$_2$,thf**) (**+** *n*-hexane).

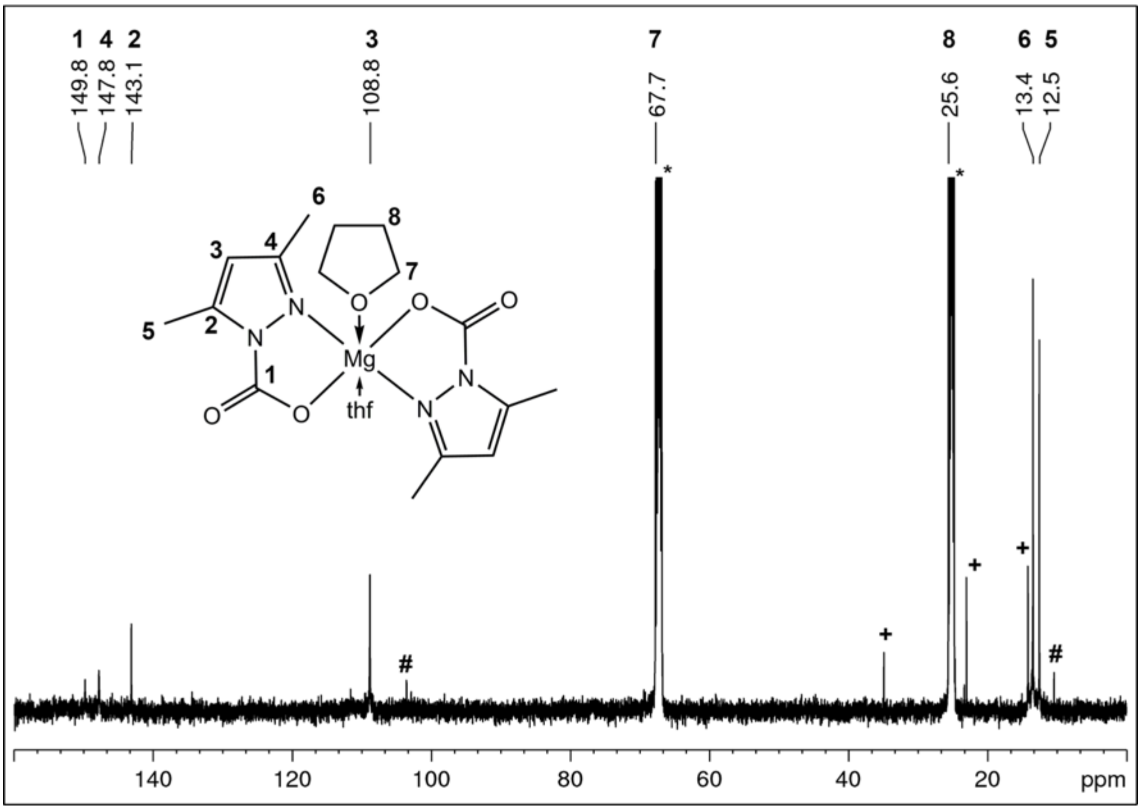

Figure S49. ^{13}C NMR spectrum (26 °C, 125.76 MHz, [D$_8$]THF) of $[Mg(CO_2{\cdot}pz^{Me,Me})_2]_n$ (**7-CO$_2$,thf**) (**+** *n*-hexane/**#** impurities).

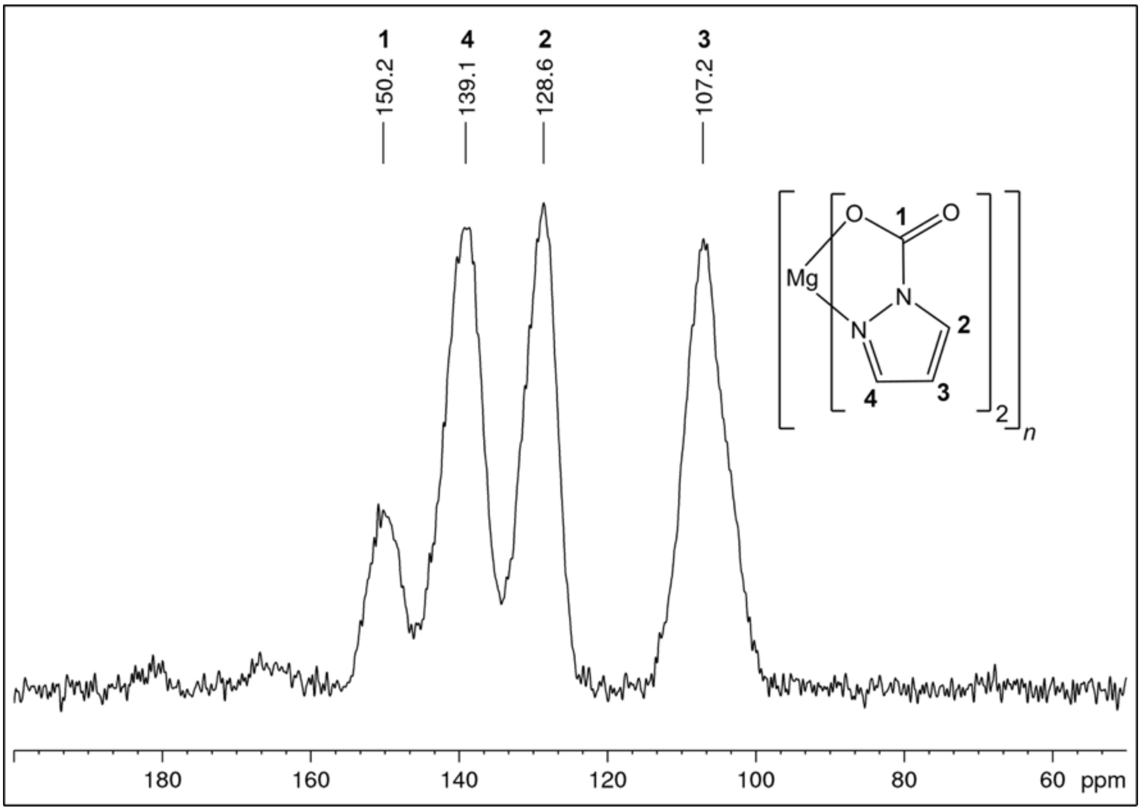

Figure S50. ^{13}C CP/MAS NMR spectrum (75.47 MHz, MAS at 8 kHz) of $[Mg(CO_2{\cdot}pz)_2]_n$ (**8-CO₂**).

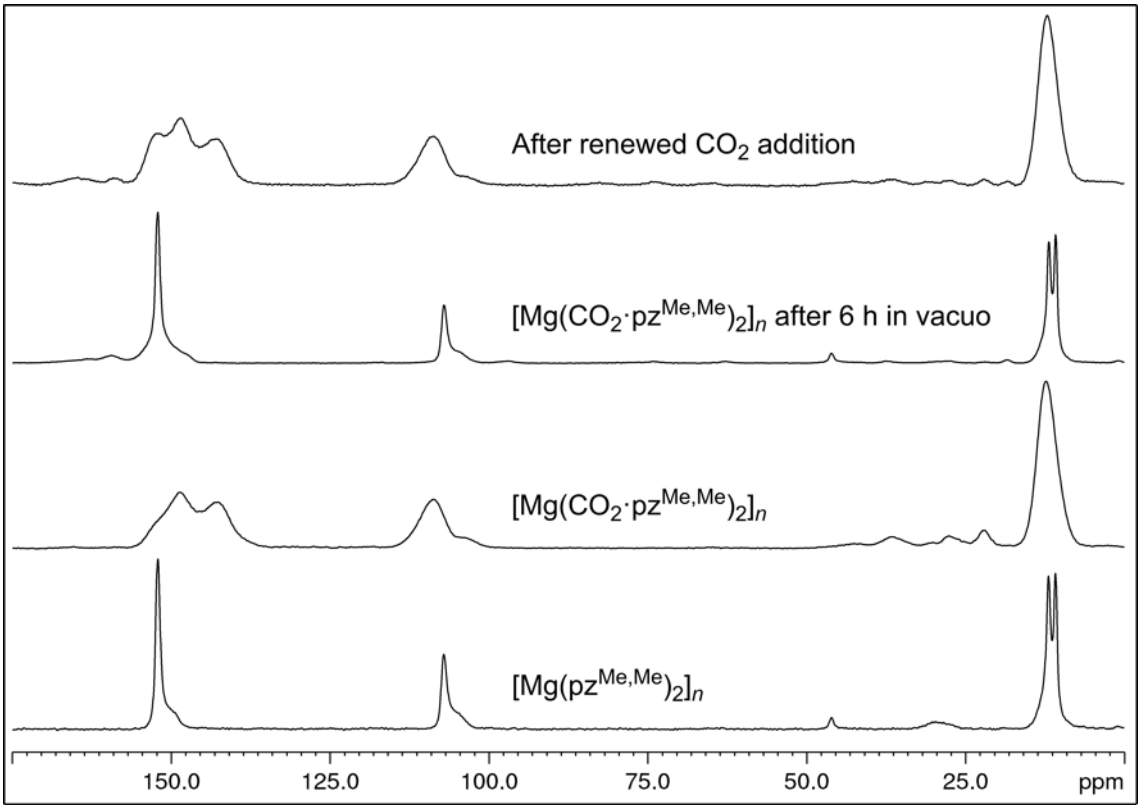

Figure S51. CO_2 insertion and release behavior of $[Mg(pz^{Me,Me})_2]_n$ (**7**) and $[Mg(CO_2{\cdot}pz^{Me,Me})_2]_n$ (**7-CO₂**) monitored by ^{13}C CP/MAS NMR spectra (75.47 MHz, MAS at 8 kHz).

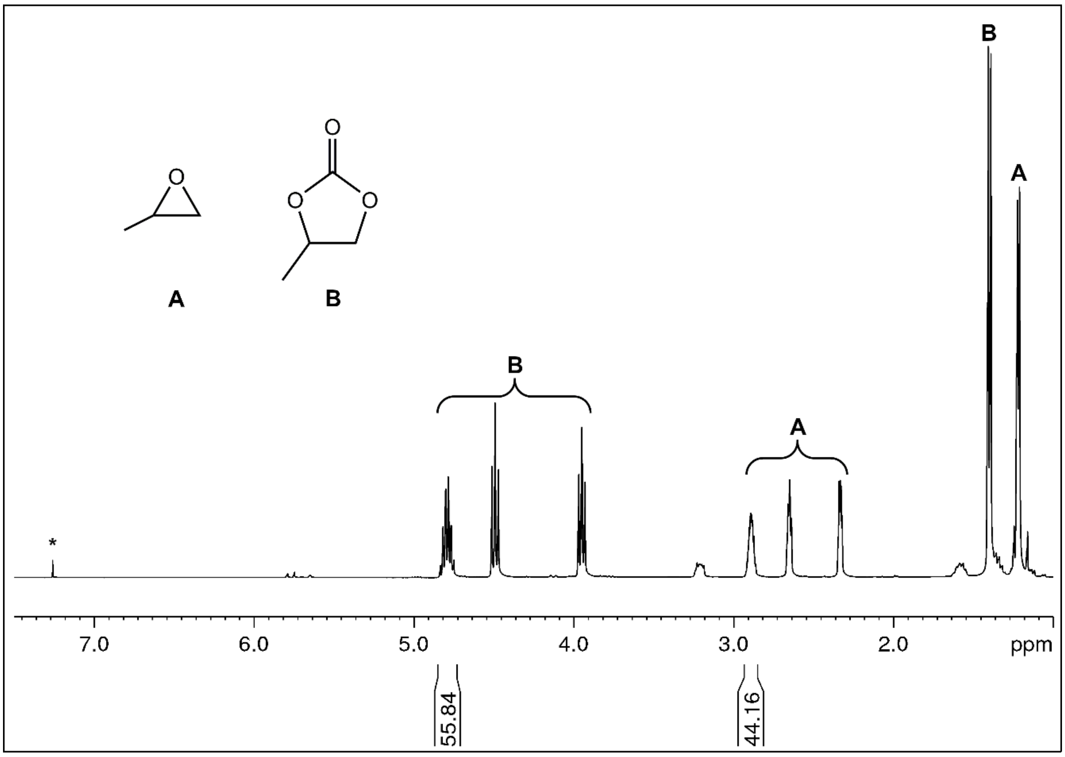

Figure S52. ^{1}H NMR spectrum (26 °C, 400 MHz, chloroform-*d*) of the reaction mixture of the catalytic conversion of propylene oxide and CO_2 to propylene carbonate by using 0.5 mol% (in relation to Mg) of $[Mg(pz^{tBu,tBu})_2]_2$ (**1**).

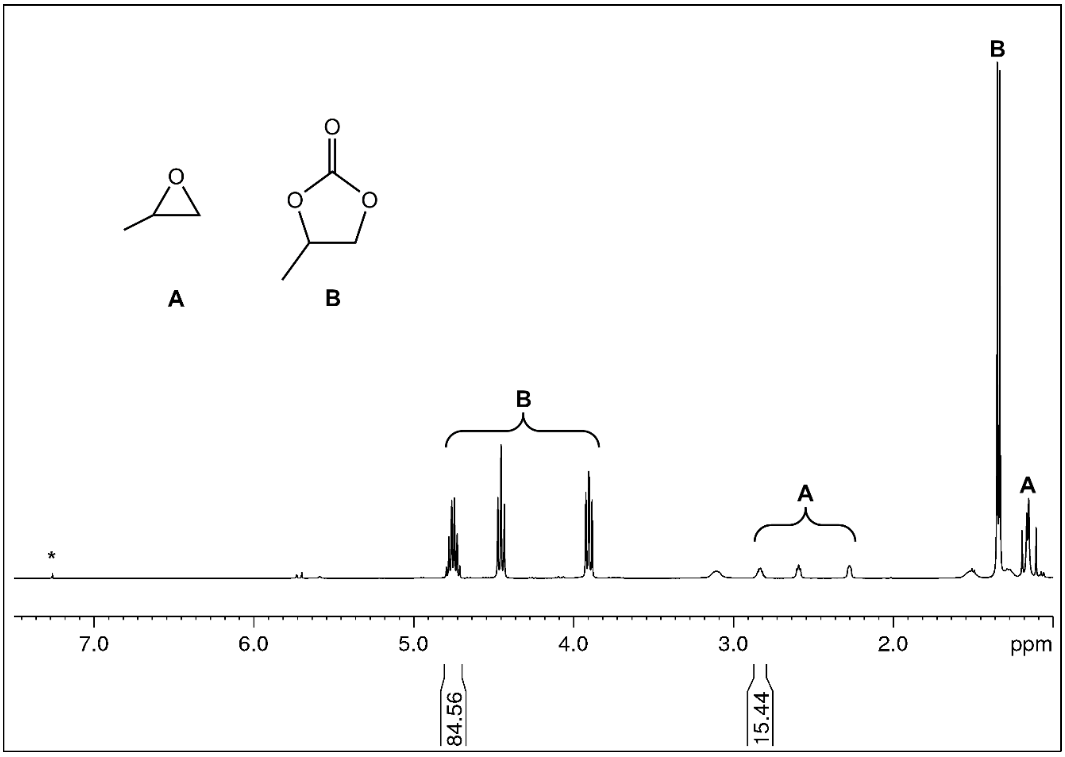

Figure S53. ^{1}H NMR spectrum (26 °C, 400 MHz, chloroform-*d*) of the reaction mixture of the catalytic conversion of propylene oxide and CO_2 to propylene carbonate by using 1 mol% (in relation to Mg) of $[Mg(pz^{tBu,tBu})_2]_2$ (**1**).

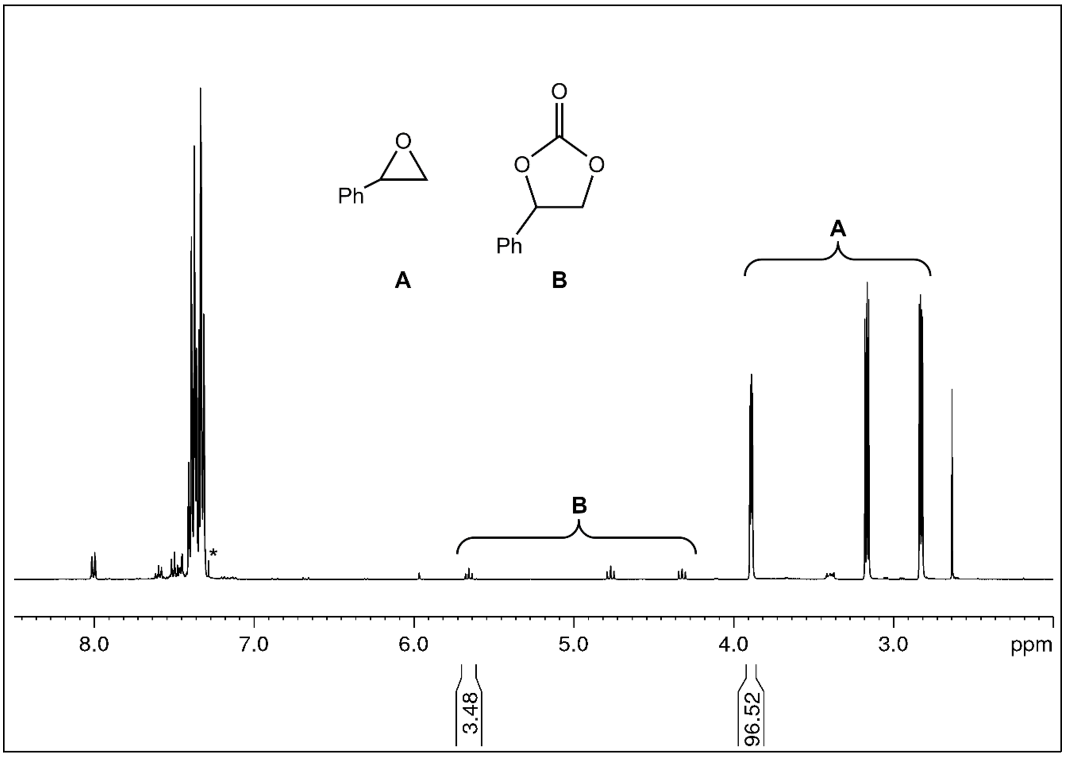

Figure S54. ^{1}H NMR spectrum (26 °C, 400 MHz, chloroform-*d*) of the reaction mixture of the catalytic conversion of styrene oxide and CO_2 to styrene carbonate by using 0.5 mol% (in relation to Mg) of [Mg(pz^{tBu,tBu})$_2$]$_2$ (**1**).

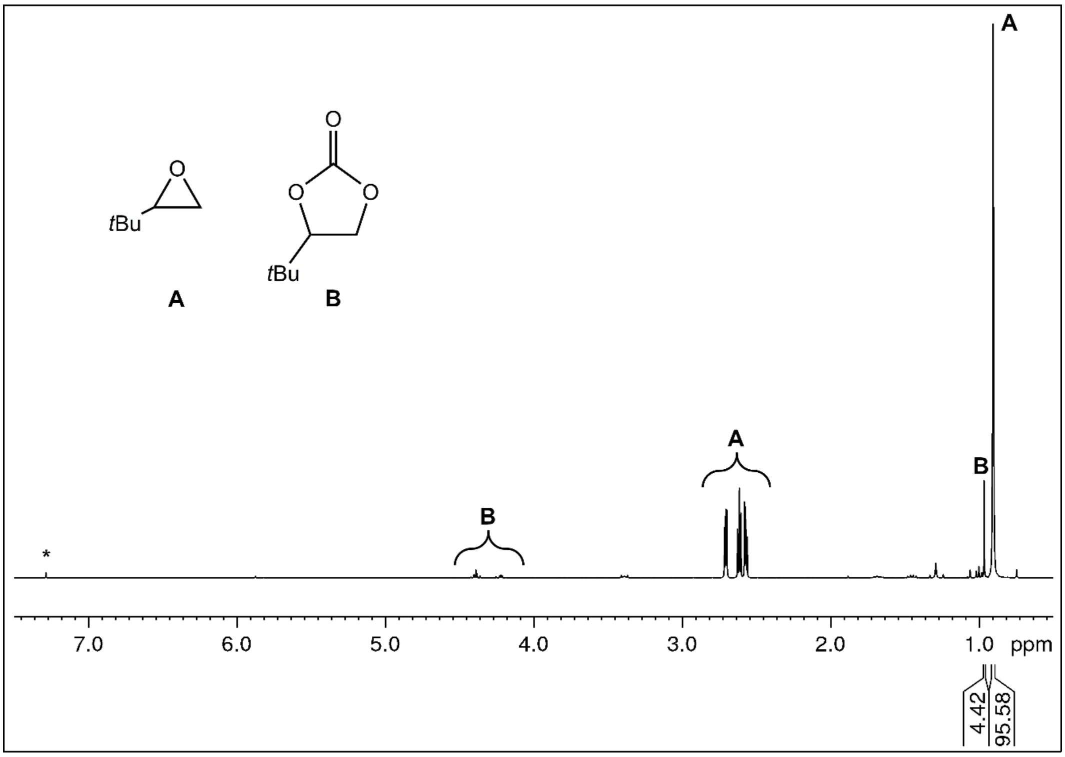

Figure S55. ^{1}H NMR spectrum (26 °C, 400 MHz, chloroform-*d*) of the reaction mixture of the catalytic conversion of 2-*tert*-butyloxirane and CO_2 to 3,3-dimethyl-1,2-butene carbonate by using 0.5 mol% (in relation to Mg) of [Mg(pz^{tBu,tBu})$_2$]$_2$ (**1**).

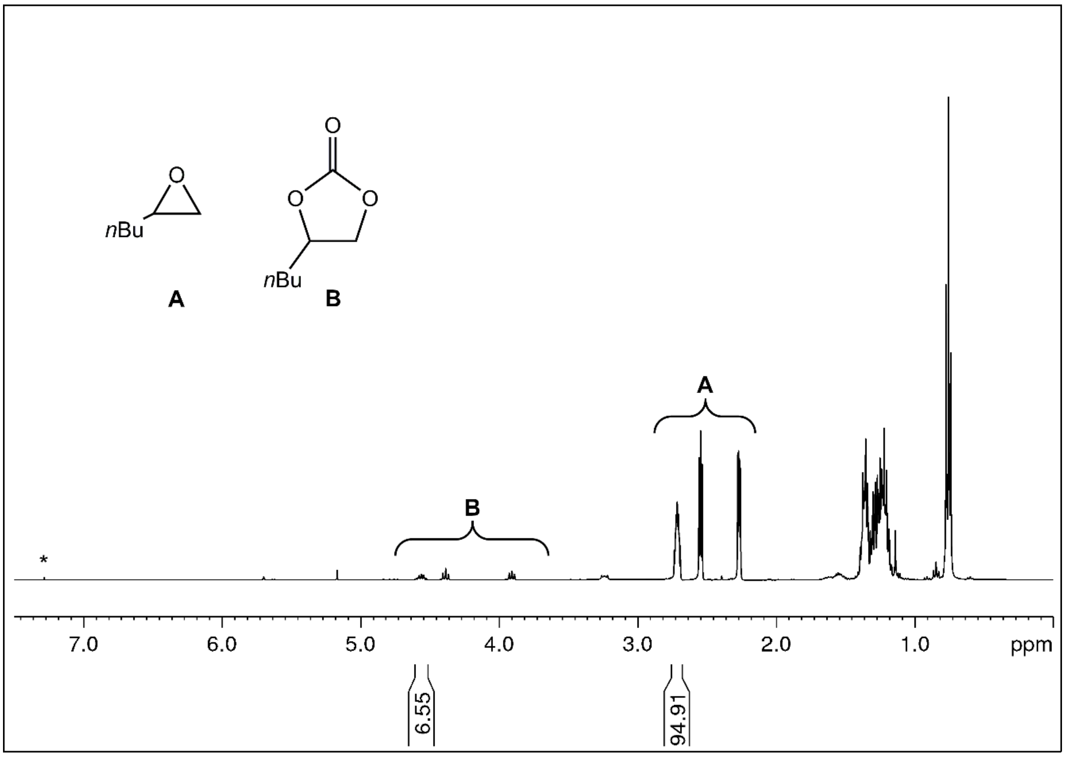

Figure S56. ^{1}H NMR spectrum (26 °C, 400 MHz, chloroform-*d*) of the reaction mixture of the catalytic conversion of 1,2-epoxyhexane and CO_2 to 1,2-*n*-hexylene carbonate by using 0.5 mol% (in relation to Mg) of $[Mg(pz^{tBu,tBu})_2]_2$ (**1**).

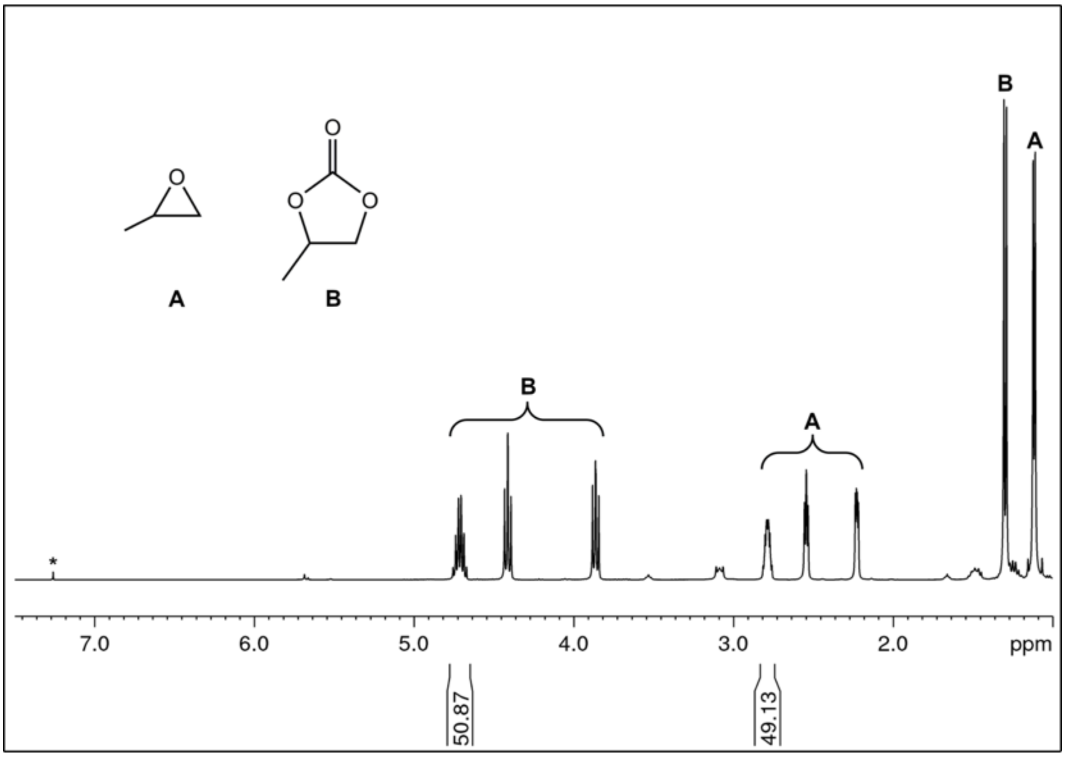

Figure S57. ^{1}H NMR spectrum (26 °C, 400 MHz, chloroform-*d*) of the reaction mixture of the catalytic conversion of propylene oxide and CO_2 to propylene carbonate by using 0.5 mol% (in relation to Mg) of $[Mg(pz^{tBu,tBu})_2(thf)]_2$ (**1-thf**).

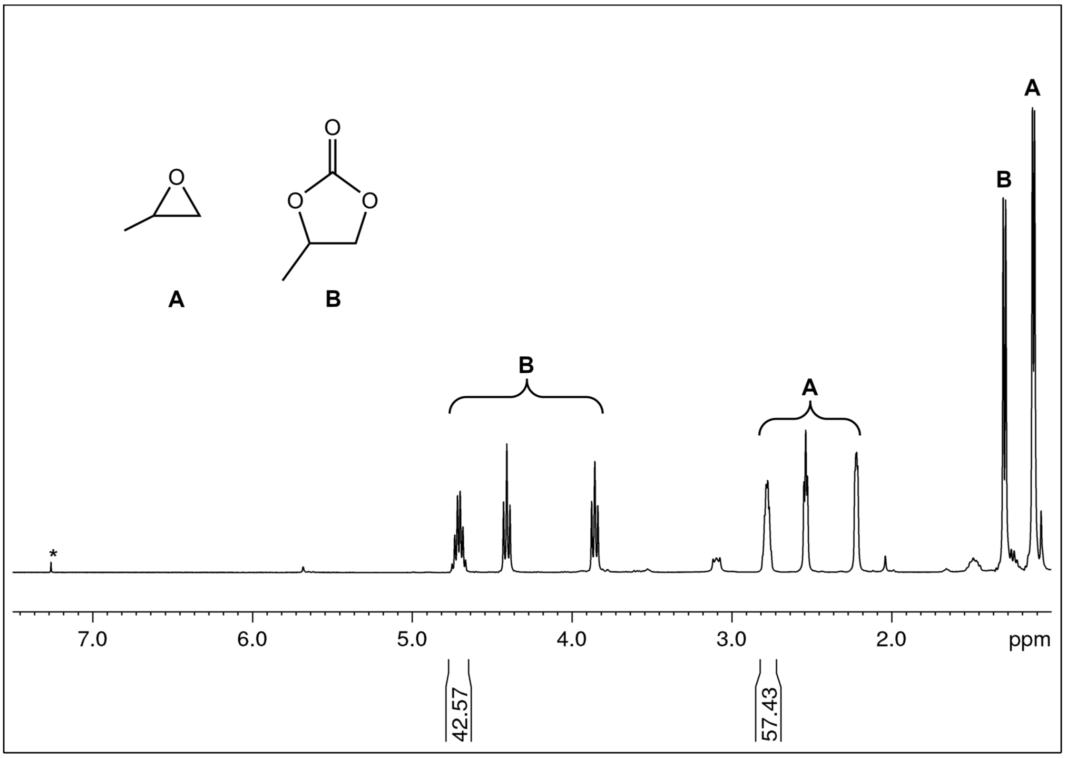

Figure S58. ^{1}H NMR spectrum (26 °C, 400 MHz, chloroform-*d*) of the reaction mixture of the catalytic conversion of propylene oxide and CO_2 to propylene carbonate by using 0.5 mol% (in relation to Mg) of $[Mg(pz^{tBu,Me})_2(thf)]_2$ (**3-thf**).

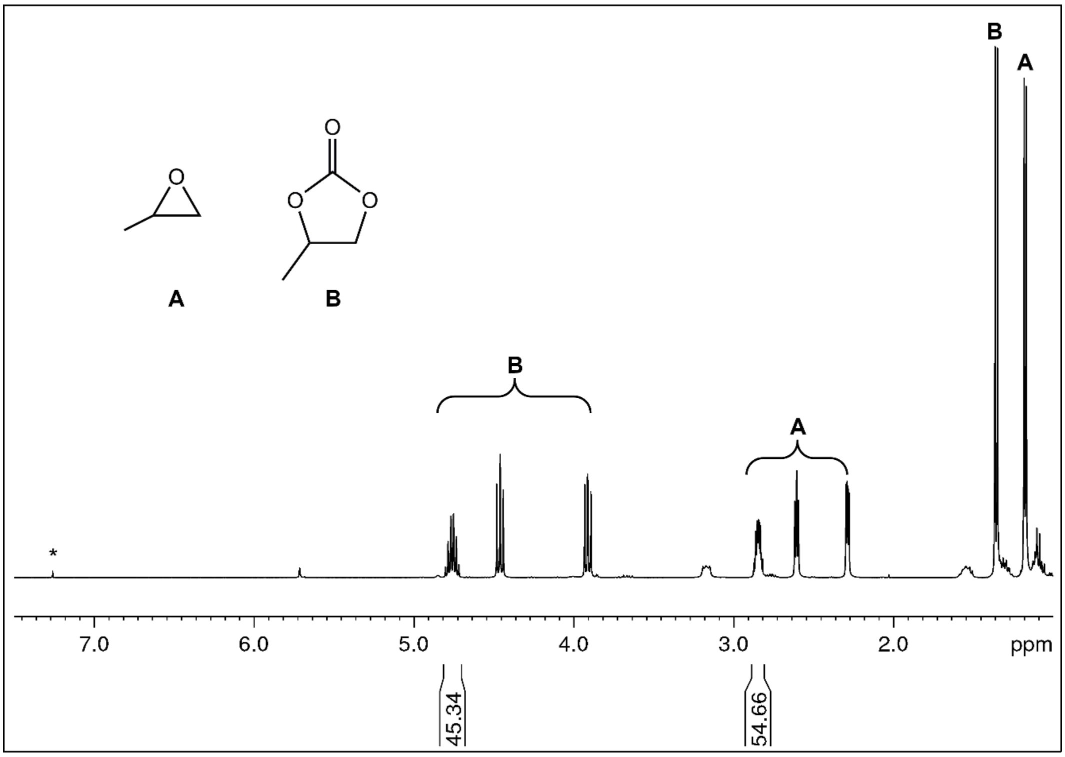

Figure S59. ^{1}H NMR spectrum (26 °C, 400 MHz, chloroform-*d*) of the reaction mixture of the catalytic conversion of propylene oxide and CO_2 to propylene carbonate by using 0.5 mol% (in relation to Mg) of $[Mg_3(pz^{iPr,iPr})_6(Hpz^{iPr,iPr})_2]$ (**4a-Hpz**).

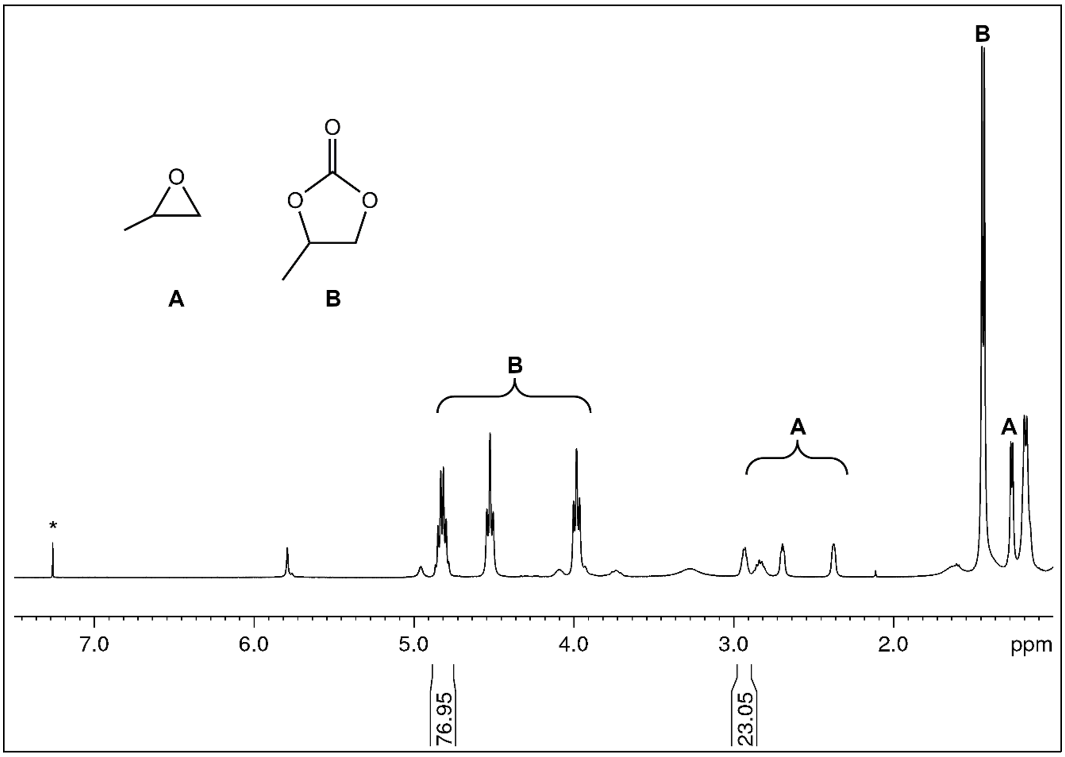

Figure S60. ^{1}H NMR spectrum (26 °C, 400 MHz, chloroform-*d*) of the reaction mixture of the catalytic conversion of propylene oxide and CO_2 to propylene carbonate by using 1 mol% (in relation to Mg) of $[Mg_3(pz^{iPr,iPr})_6(Hpz^{iPr,iPr})_2]$ (**4a-Hpz**).

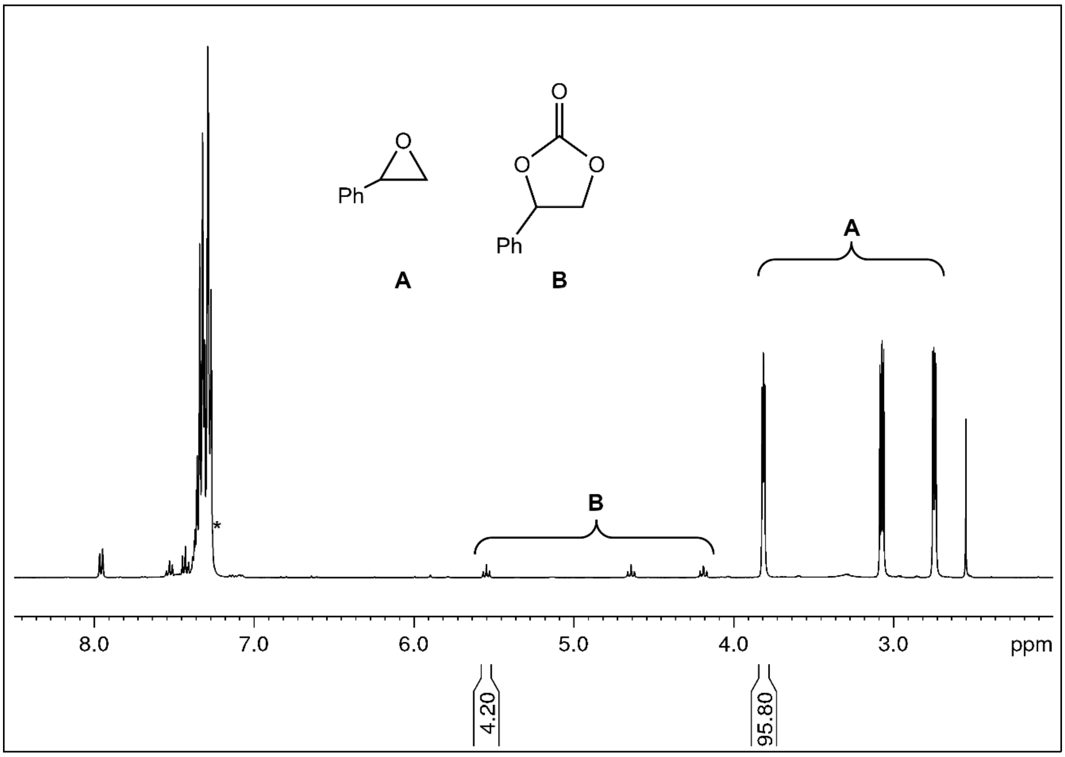

Figure S61. ^{1}H NMR spectrum (26 °C, 400 MHz, chloroform-*d*) of the reaction mixture of the catalytic conversion of styrene oxide and CO_2 to styrene carbonate by using 0.5 mol% (in relation to Mg) of $[Mg_3(pz^{iPr,iPr})_6(Hpz^{iPr,iPr})_2]$ (**4a-Hpz**).

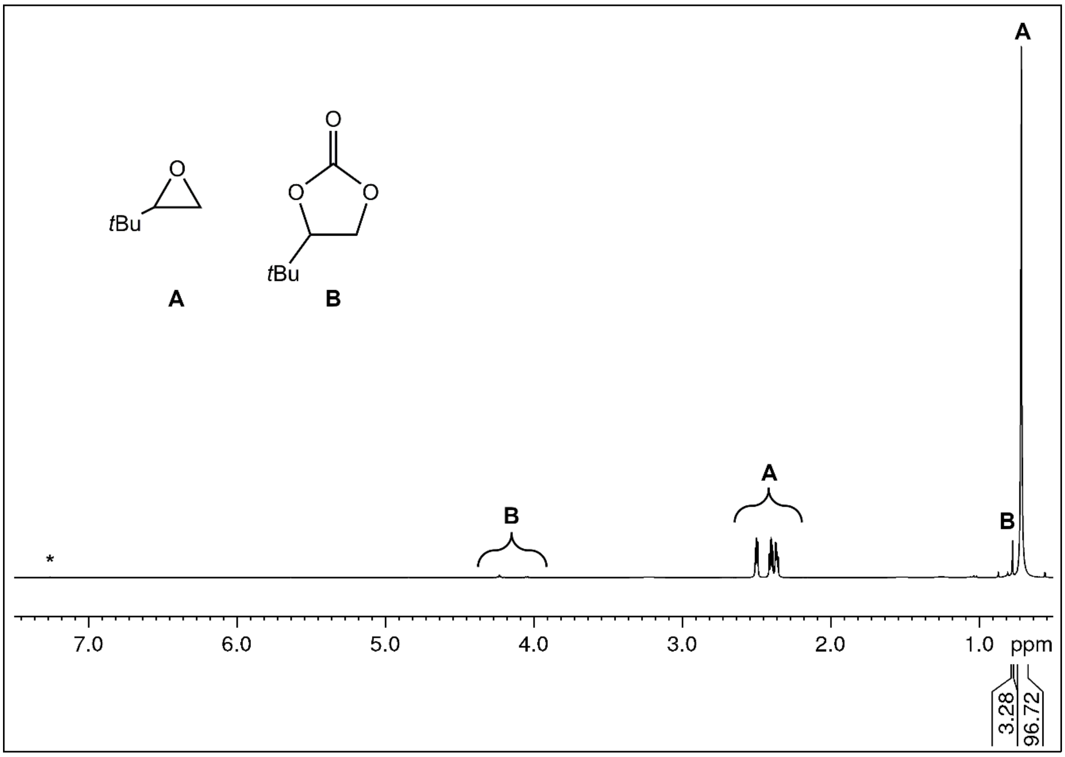

Figure S62. ^{1}H NMR spectrum (26 °C, 400 MHz, chloroform-*d*) of the reaction mixture of the catalytic conversion of 2-*tert*-butyloxirane and CO_2 to 3,3-dimethyl-1,2-butene carbonate by using 0.5 mol% (in relation to Mg) of [$Mg_3(pz^{iPr,iPr})_6(Hpz^{iPr,iPr})_2$] (**4a-Hpz**).

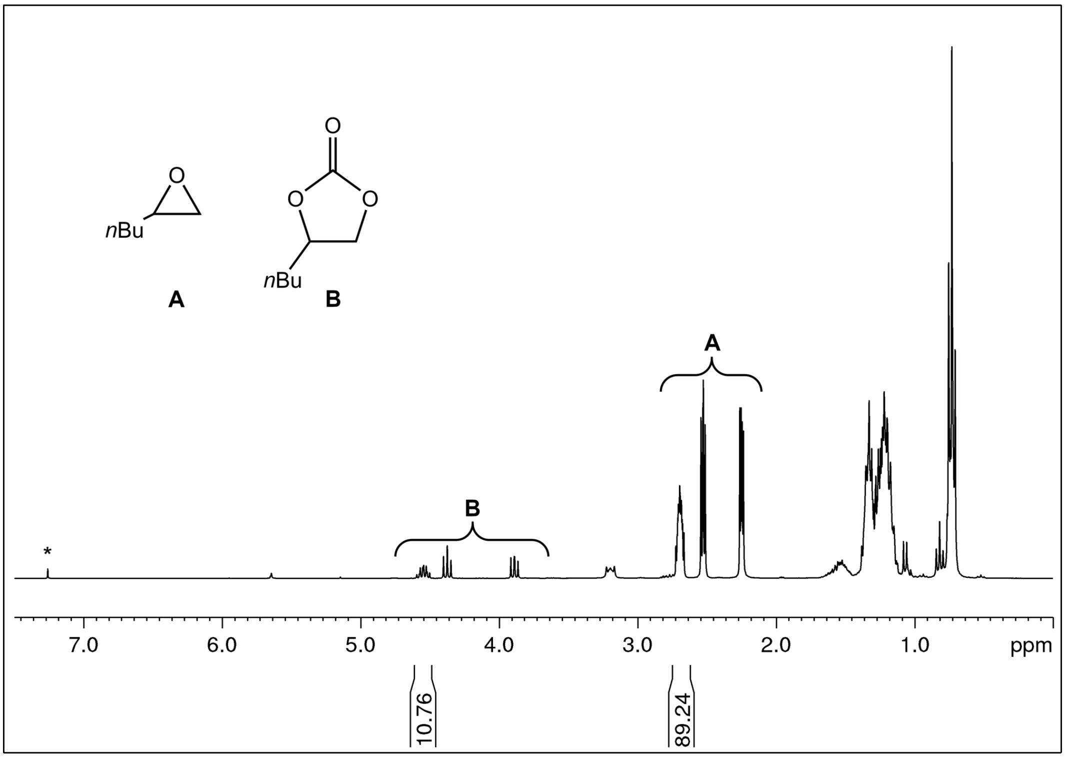

Figure S63. ^{1}H NMR spectrum (26 °C, 400 MHz, chloroform-*d*) of the reaction mixture of the catalytic conversion of 1,2-epoxyhexane and CO_2 to 1,2-*n*-hexylene carbonate by using 0.5 mol% (in relation to Mg) of [$Mg_3(pz^{iPr,iPr})_6(Hpz^{iPr,iPr})_2$] (**4a-Hpz**).

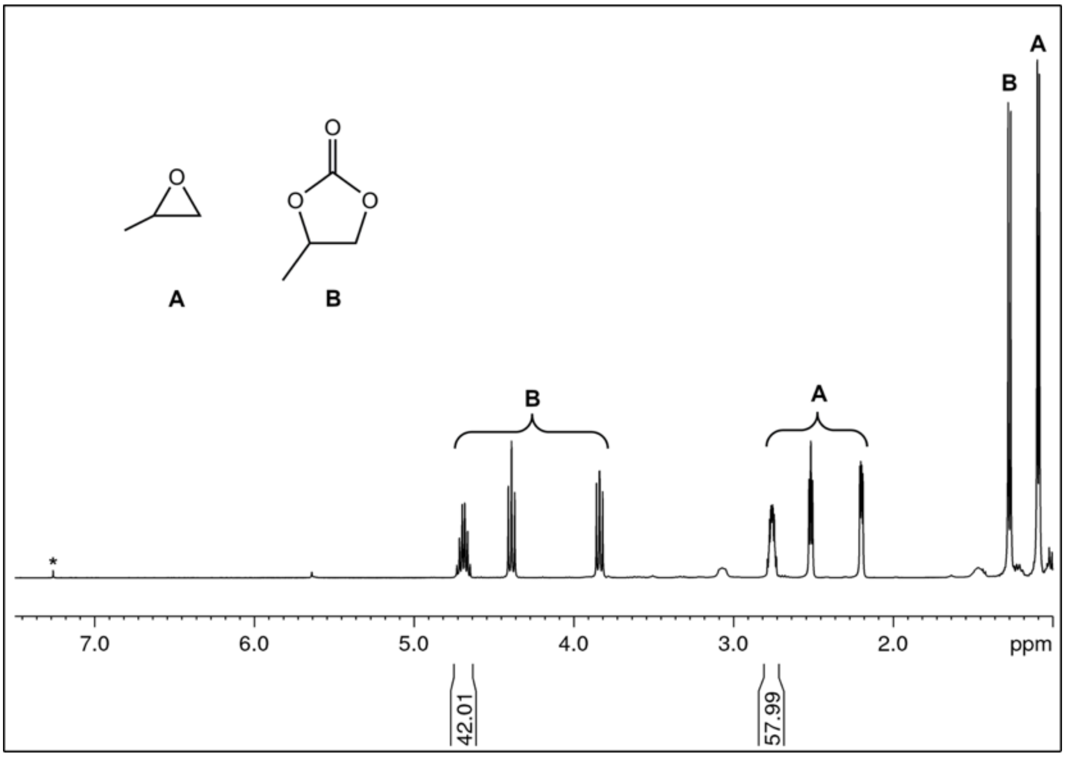

Figure S64. ^{1}H NMR spectrum (26 °C, 400 MHz, chloroform-*d*) of the reaction mixture of the catalytic conversion of propylene oxide and CO_2 to propylene carbonate by using 0.5 mol% (in relation to Mg) of $[Mg_3(pz^{iPr,iPr})_6(thf)_2]$ (**4-thf**).

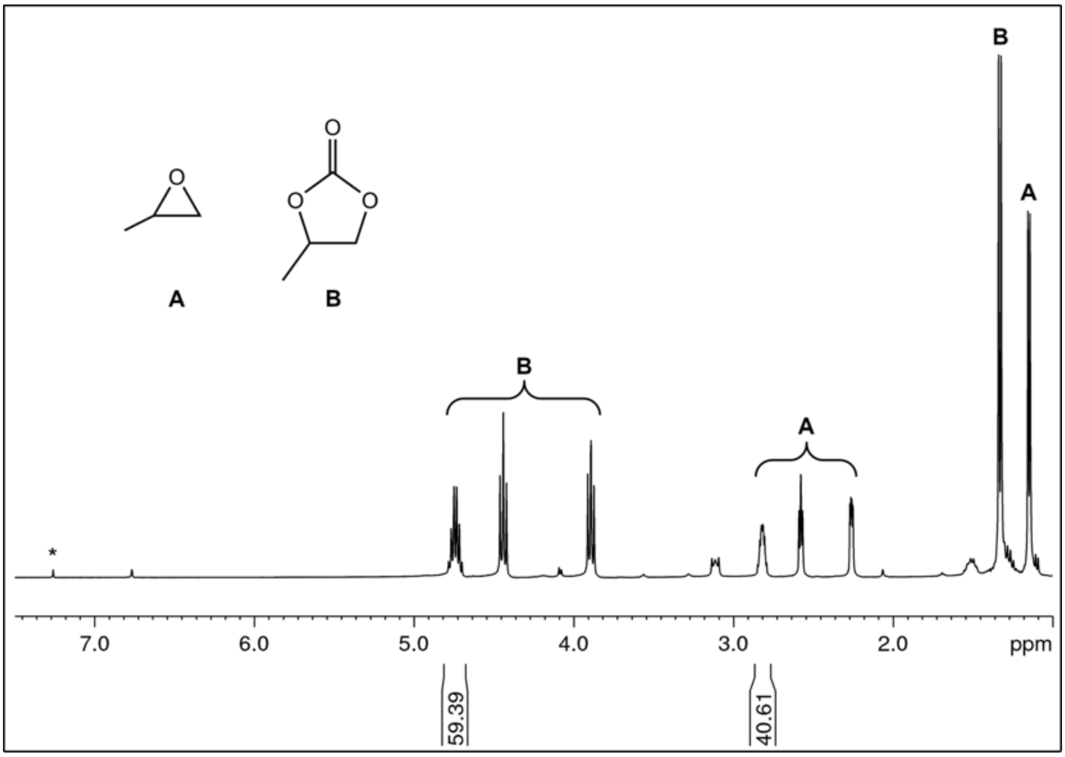

Figure S65. ^{1}H NMR spectrum (26 °C, 400 MHz, chloroform-*d*) of the reaction mixture of the catalytic conversion of propylene oxide and CO_2 to propylene carbonate by using 0.5 mol% (in relation to Mg) of $[Mg_2(pz^{CF_3,CF_3})_4(thf)_3]$ (**6-thf**).

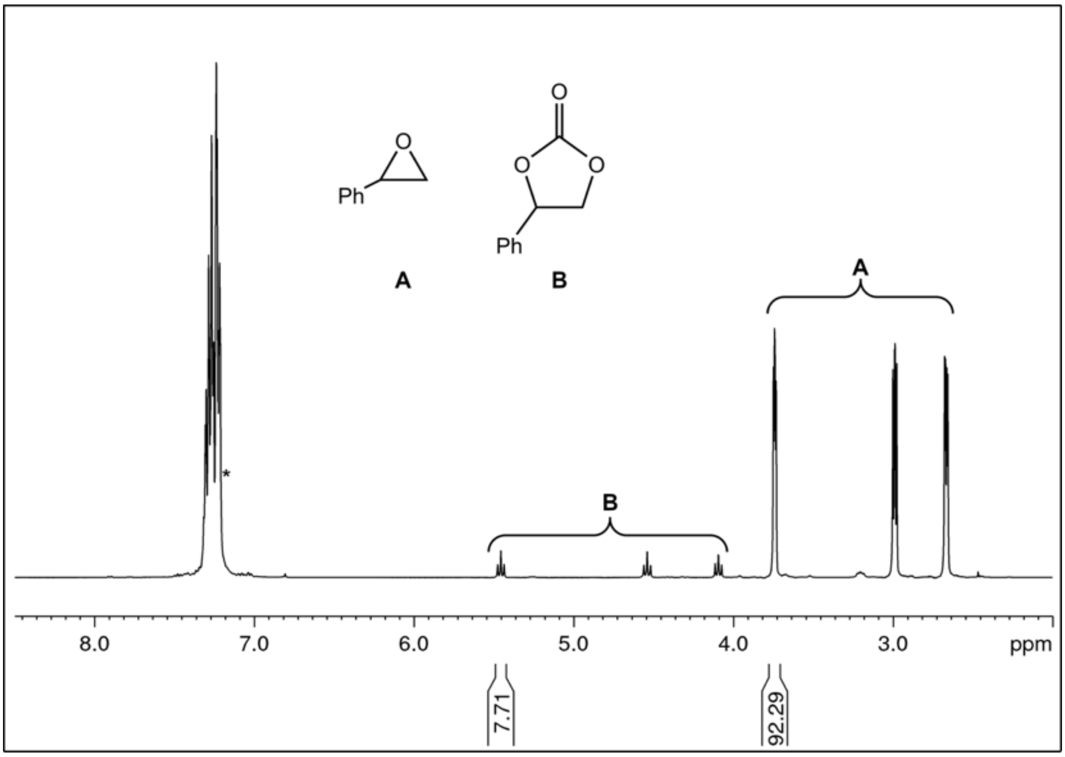

Figure S66. ^{1}H NMR spectrum (26 °C, 400 MHz, chloroform-*d*) of the reaction mixture of the catalytic conversion of styrene oxide and CO_2 to styrene carbonate by using 0.5 mol% (in relation to Mg) of [$Mg_2(pz^{CF_3,CF_3})_4(thf)_3$] (**6-thf**).

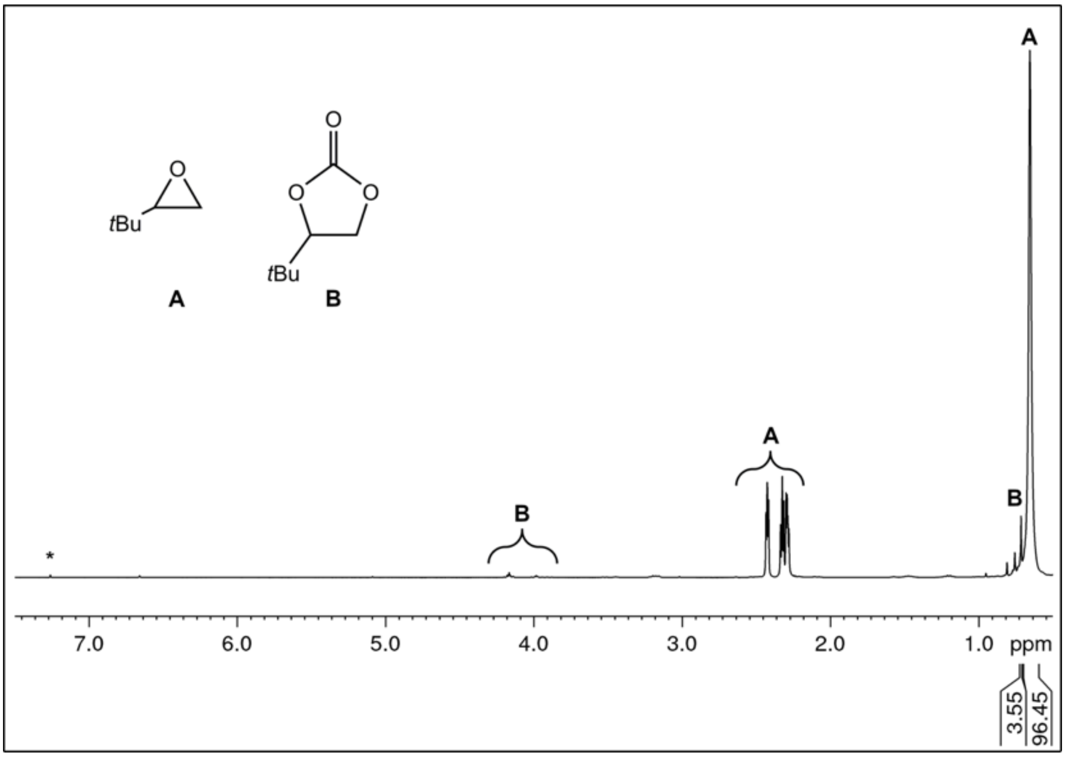

Figure S67. ^{1}H NMR spectrum (26 °C, 400 MHz, chloroform-*d*) of the reaction mixture of the catalytic conversion of 2-*tert*-butyloxirane and CO_2 to 3,3-dimethyl-1,2-butene carbonate by using 0.5 mol% (in relation to Mg) of [$Mg_2(pz^{CF_3,CF_3})_4(thf)_3$] (**6-thf**).

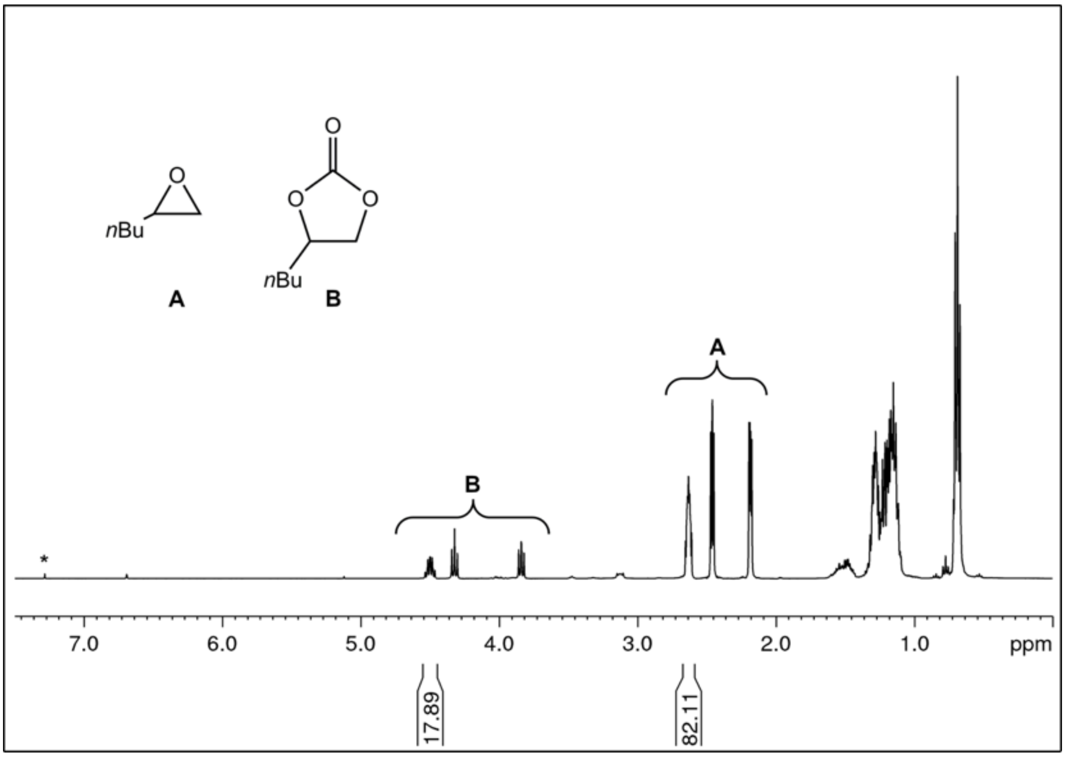

Figure S68. ^{1}H NMR spectrum (26 °C, 400 MHz, chloroform-*d*) of the reaction mixture of the catalytic conversion of 1,2-epoxyhexane and CO_2 to 1,2-*n*-hexylene carbonate by using 0.5 mol% (in relation to Mg) of $[Mg_2(pz^{CF_3,CF_3})_4(thf)_3]$ (**6-thf**).

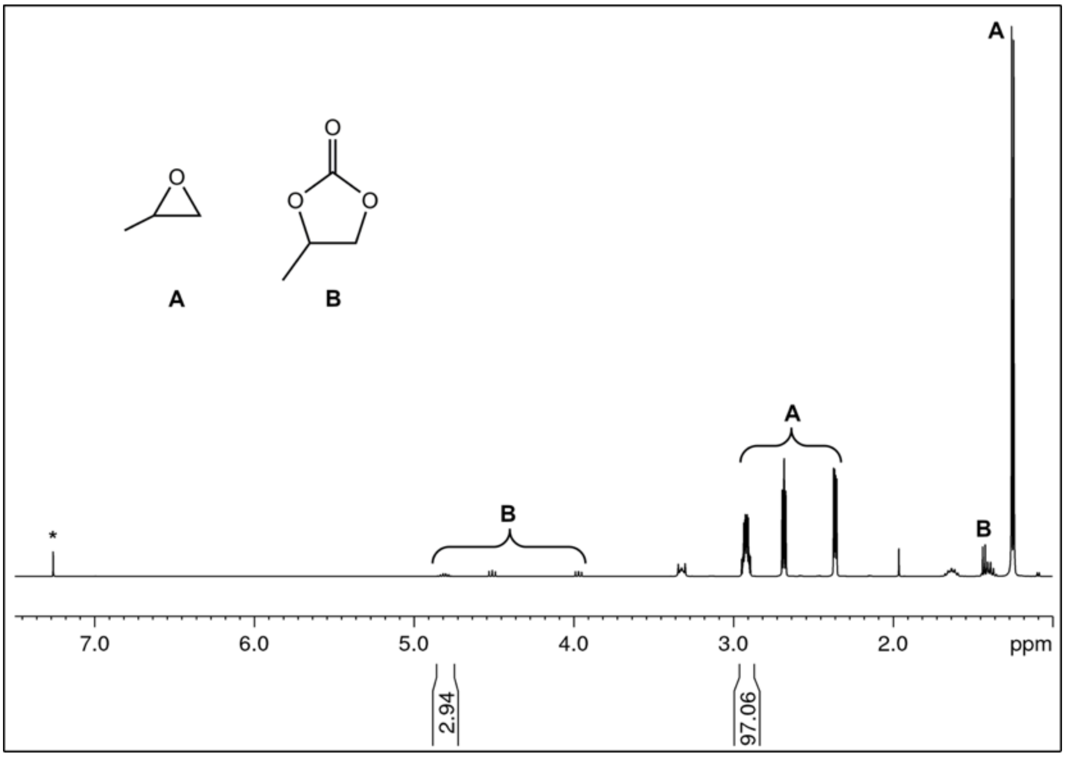

Figure S69. ^{1}H NMR spectrum (26 °C, 400 MHz, chloroform-*d*) of the reaction mixture of the conversion of propylene oxide and CO_2 to propylene carbonate in the absence of magnesium pyrazlate, by using only 1 mol% TBAB as catalyst.

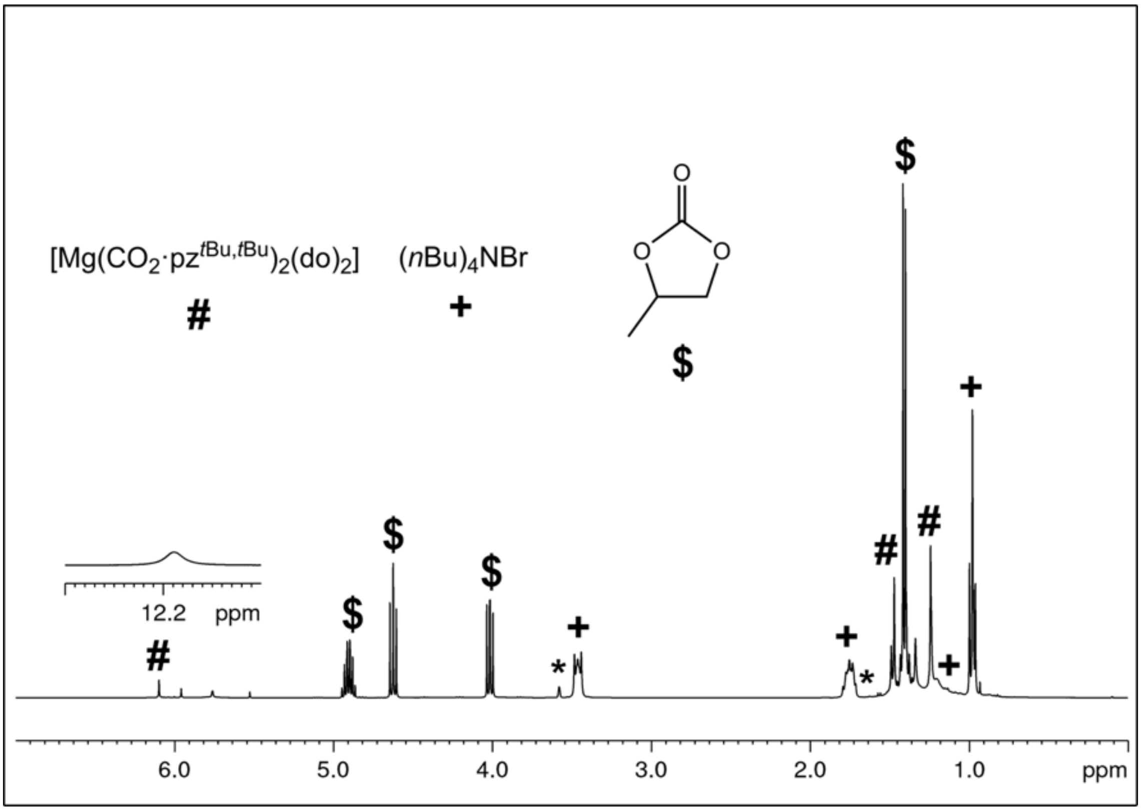

Figure S70. ^{1}H NMR spectrum (26 °C, 400 MHz, [D_8]THF) of the reaction mixture of the catalytic conversion of propylene oxide and CO_2 to propylene carbonate by using 10 mol% (in relation to Mg) of [Mg($pz^{tBu,tBu}$)$_2$]$_2$ (**1**). Visible products are [Mg(CO_2·$pz^{tBu,tBu}$)$_2$(thf)$_2$] (#), propylene carbonate ($), and TBAB (+).

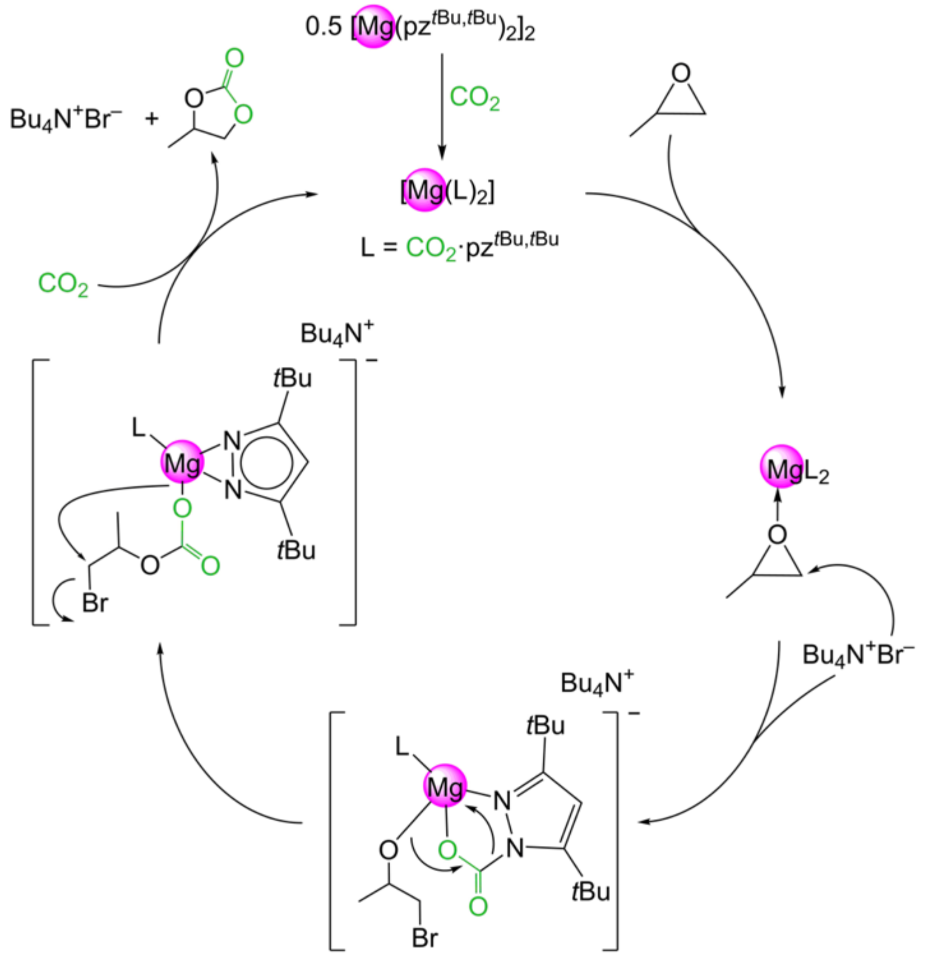

Scheme S2. Proposed mechanism for the catalytic conversion of propylene oxide to propylene carbonate with **1** as an exemplary catalyst.

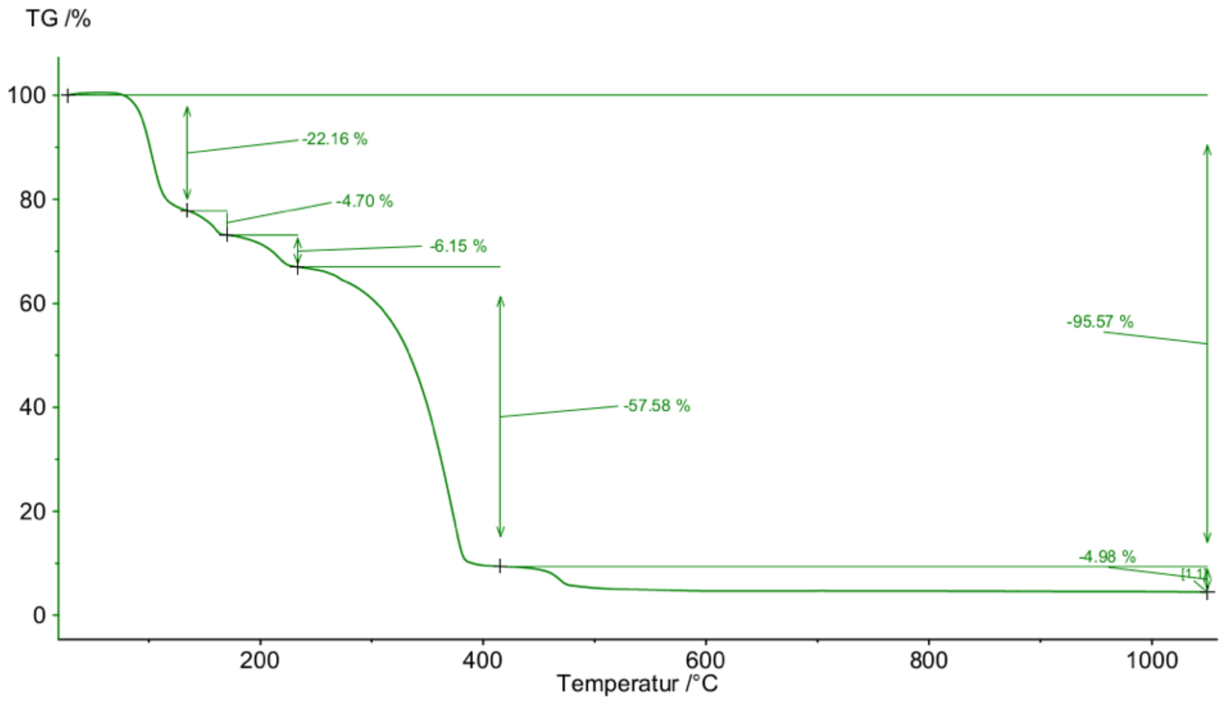

Figure S71. TGA of [Mg(CO_2·pz^{tBu,tBu})$_2$(thf)$_2$] (**1-CO₂,thf**). Sample was heated from 28 °C to 1000 °C with a heating ratio of 1 Kmin^{-1} under constant Ar flow.

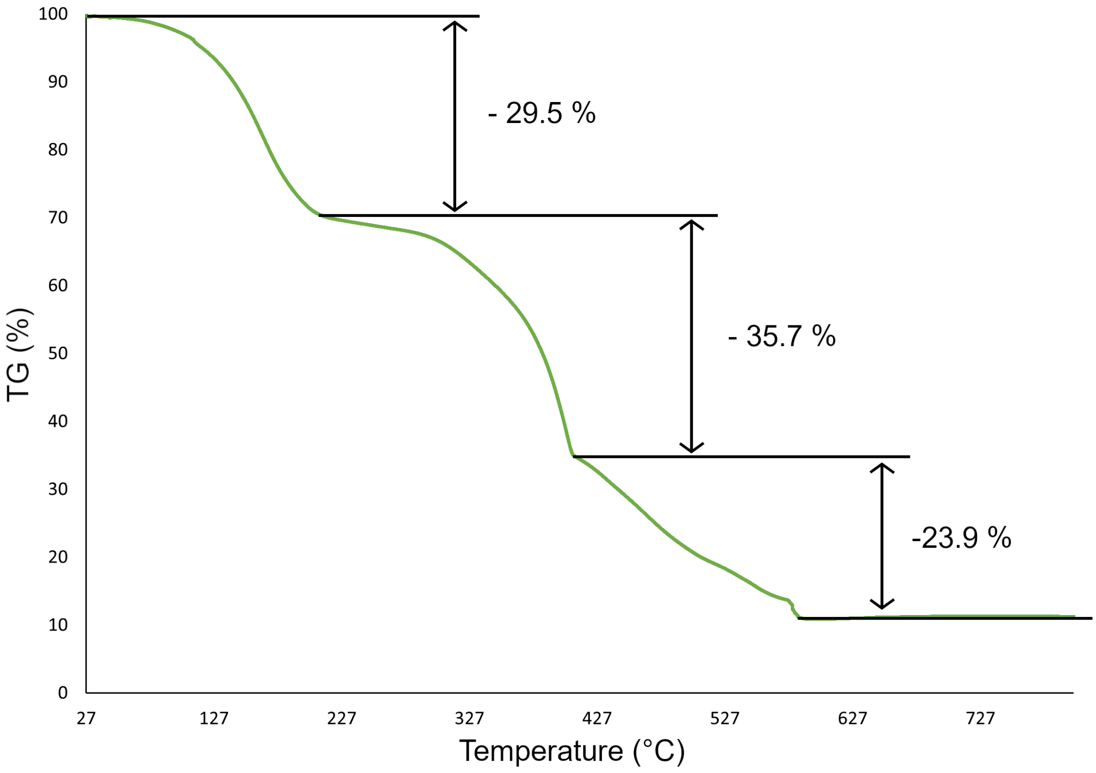

Figure S72. TGA of [Mg(CO_2·pz^{Me,Me})$_2$]$_n$ (**7-CO₂**). Sample was heated from 28 °C to 1000 °C with a heating ratio of 1 Kmin^{-1} under constant Ar flow.

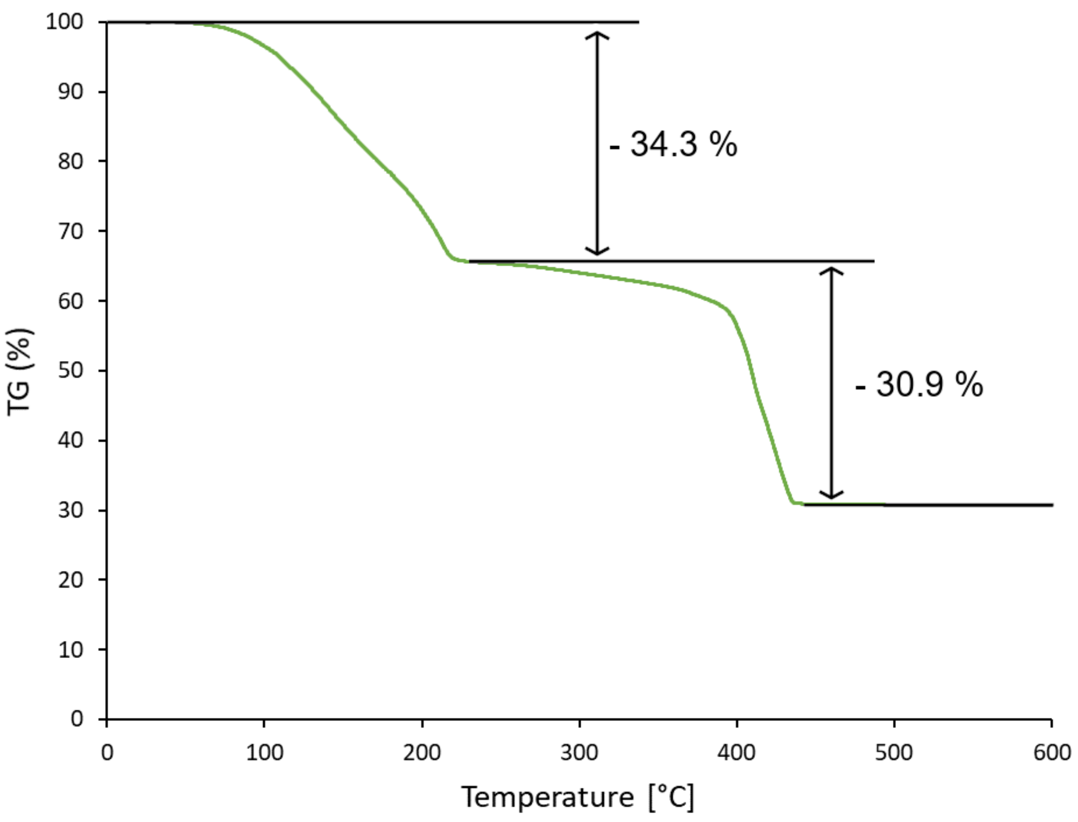

Figure S73. TGA of $[Mg(CO_2{\cdot}pz)_2]_n$ (**8-CO₂**). Sample was heated from 28 °C to 1000 °C with a heating ratio of 1 Kmin^{-1} under constant Ar flow.

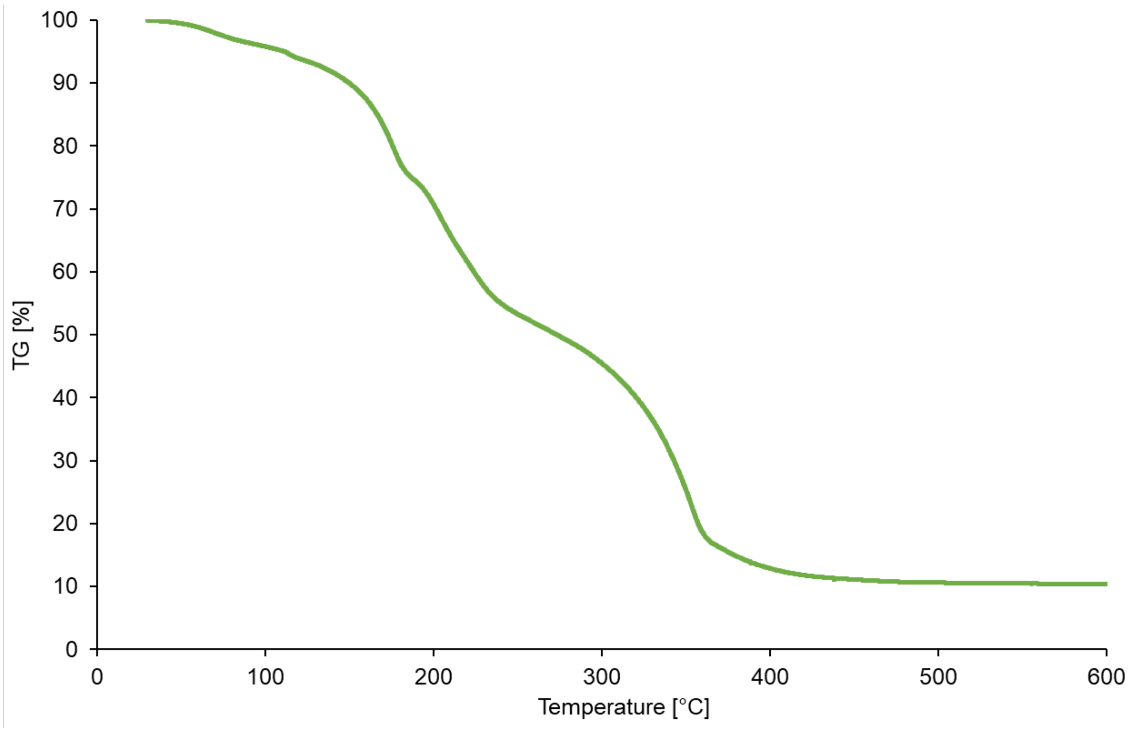

Figure S74. TGA of $[Mg(CO_2{\cdot}pz^{tBu,tBu})_2]_4$ (**1-CO₂**). Sample was heated from 28 °C to 1000 °C with a heating ratio of 1 Kmin^{-1} under constant Ar flow.

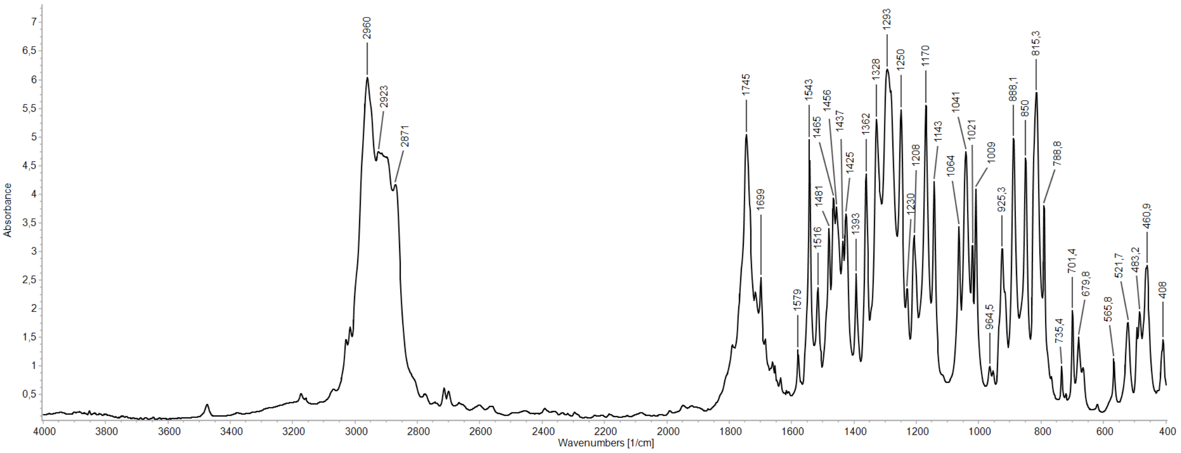

Figure S75. DRIFT spectrum of [Mg(CO_2·pz^{tBu,tBu})$_2$(thf)$_2$] (**1-CO$_2$,thf**) at 25 °C.

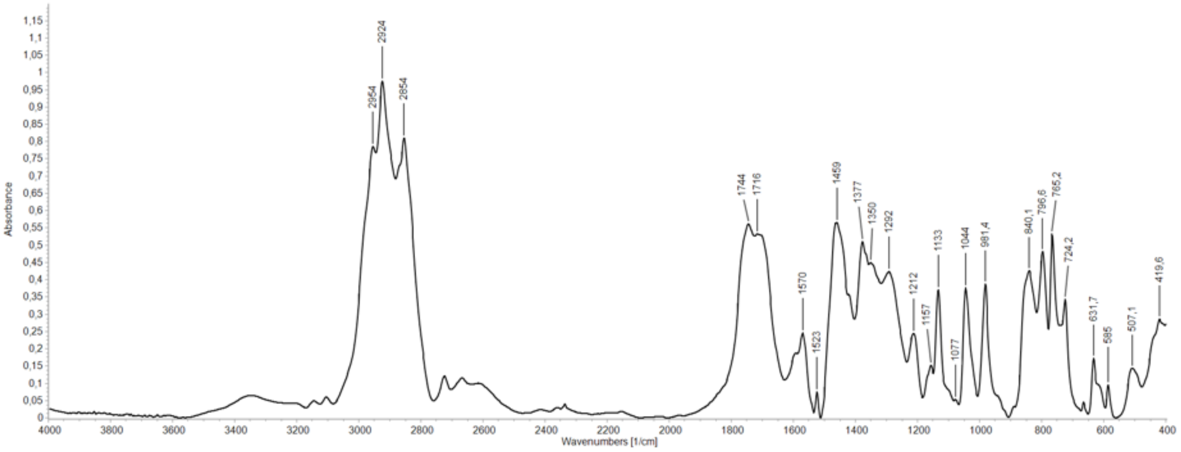

Figure S76. IR (Nujol) spectrum of [Mg(CO_2·pz^{Me,Me})$_2$]$_n$ (**7-CO$_2$**) at 25 °C.

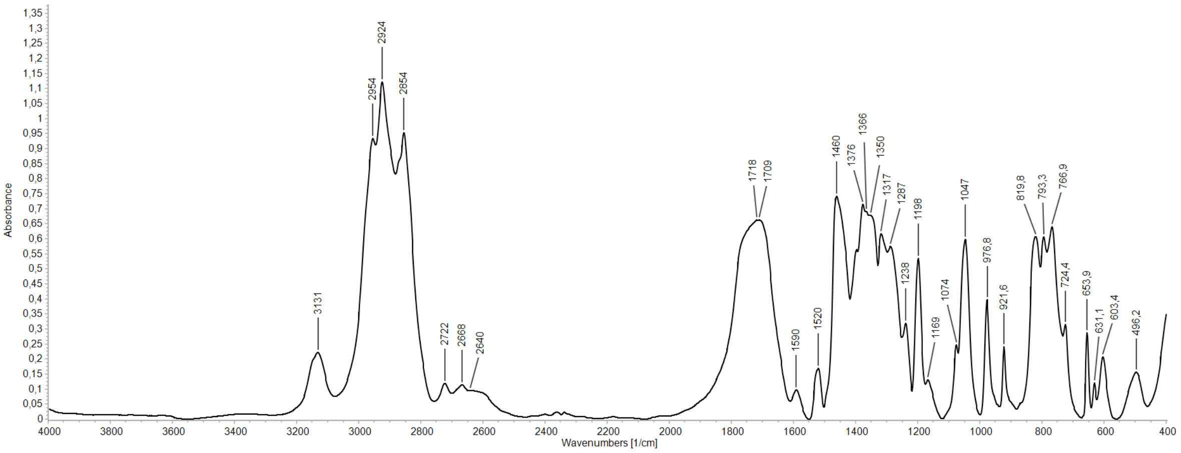

Figure S77. IR (Nujol) spectrum of [Mg(CO_2·pz)$_2$]$_n$ (**8-CO$_2$**) at 25 °C.

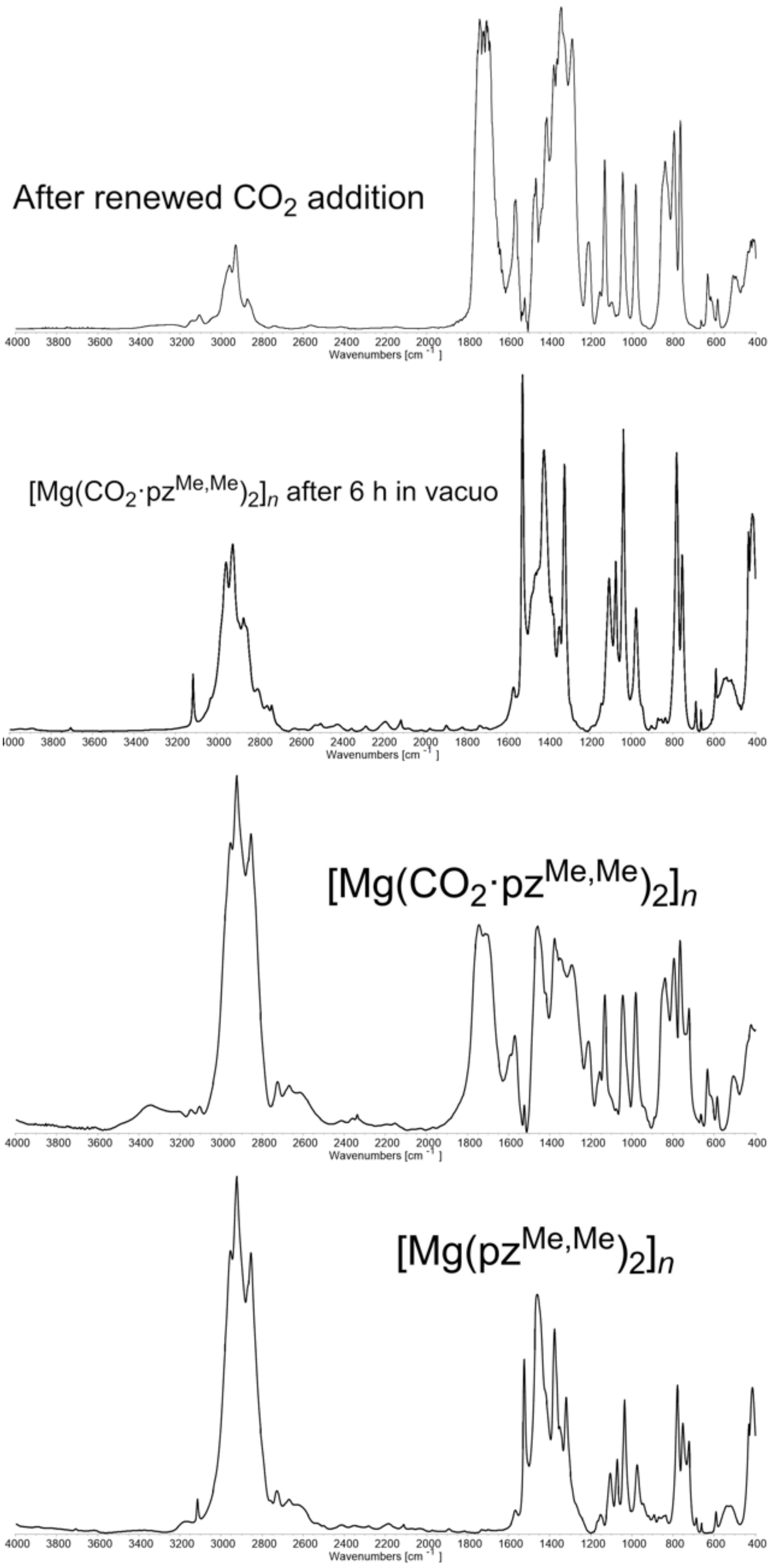

Figure S78. CO_2 insertion and release behavior of [Mg(pz^{Me,Me})$_2$]$_n$ (**7**) and [Mg(CO_2·pz^{Me,Me})$_2$]$_n$ (**7-CO₂**) monitored by IR spectra (Nujol bottom two, DRIFT top two).

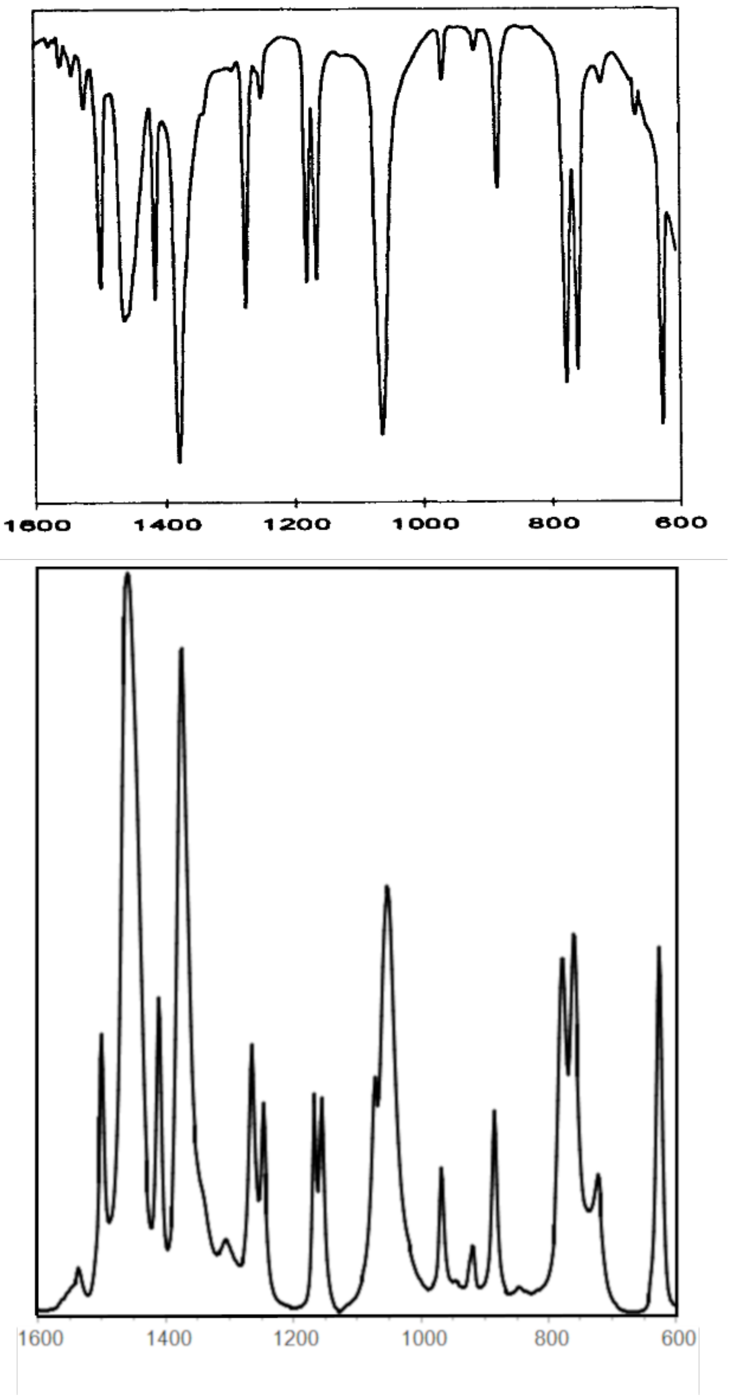

Figure S79. Comparison of the DRITF spectra of $[Mg(pz)_2]_n$ (**8**, bottom) and $[Zn(pz)_2]_n$ (top).[12]

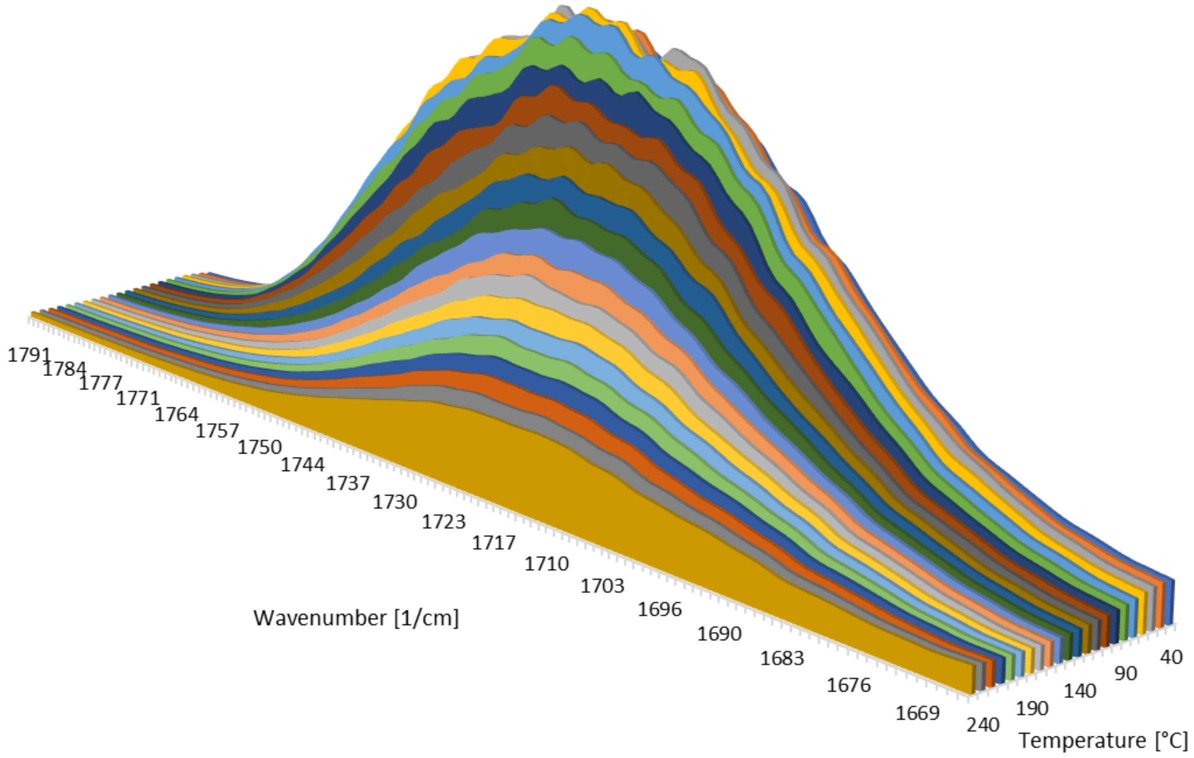

Figure S80. In situ VT DRIFT spectra of [Mg(CO_2·pz)$_2$]$_n$ (**8-CO₂**) after the atmosphere was changed from CO_2 to Ar.

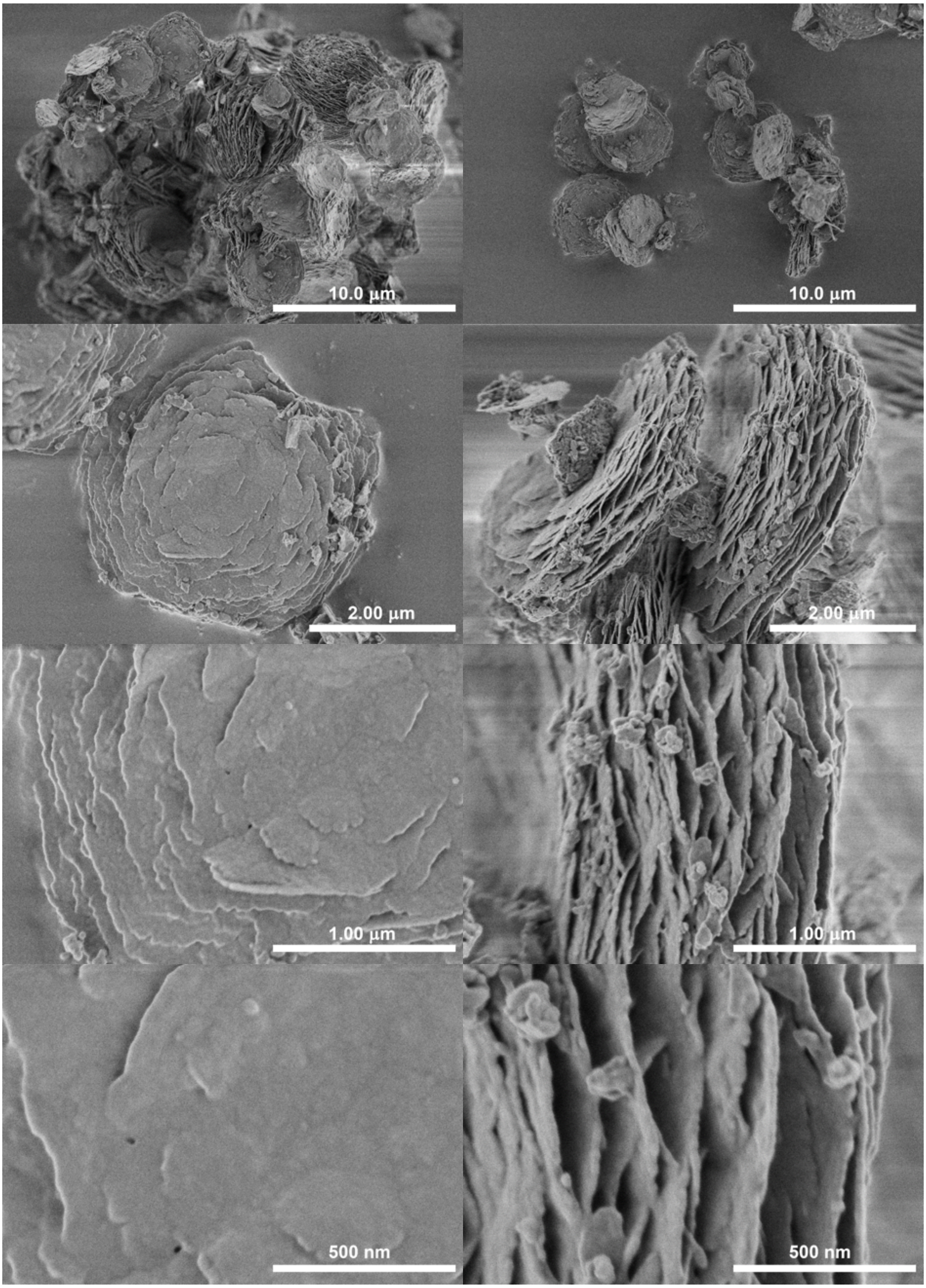

Figure S81. SEM images of $[Mg(pz)_2]_n$ (**8**). The overall morphology of **8** features thin layers (10-20 nm) of round platelets (3-6 μm). SEM images of **8-CO$_2$** were infeasible, due to immediate CO_2 release in high vacuum.

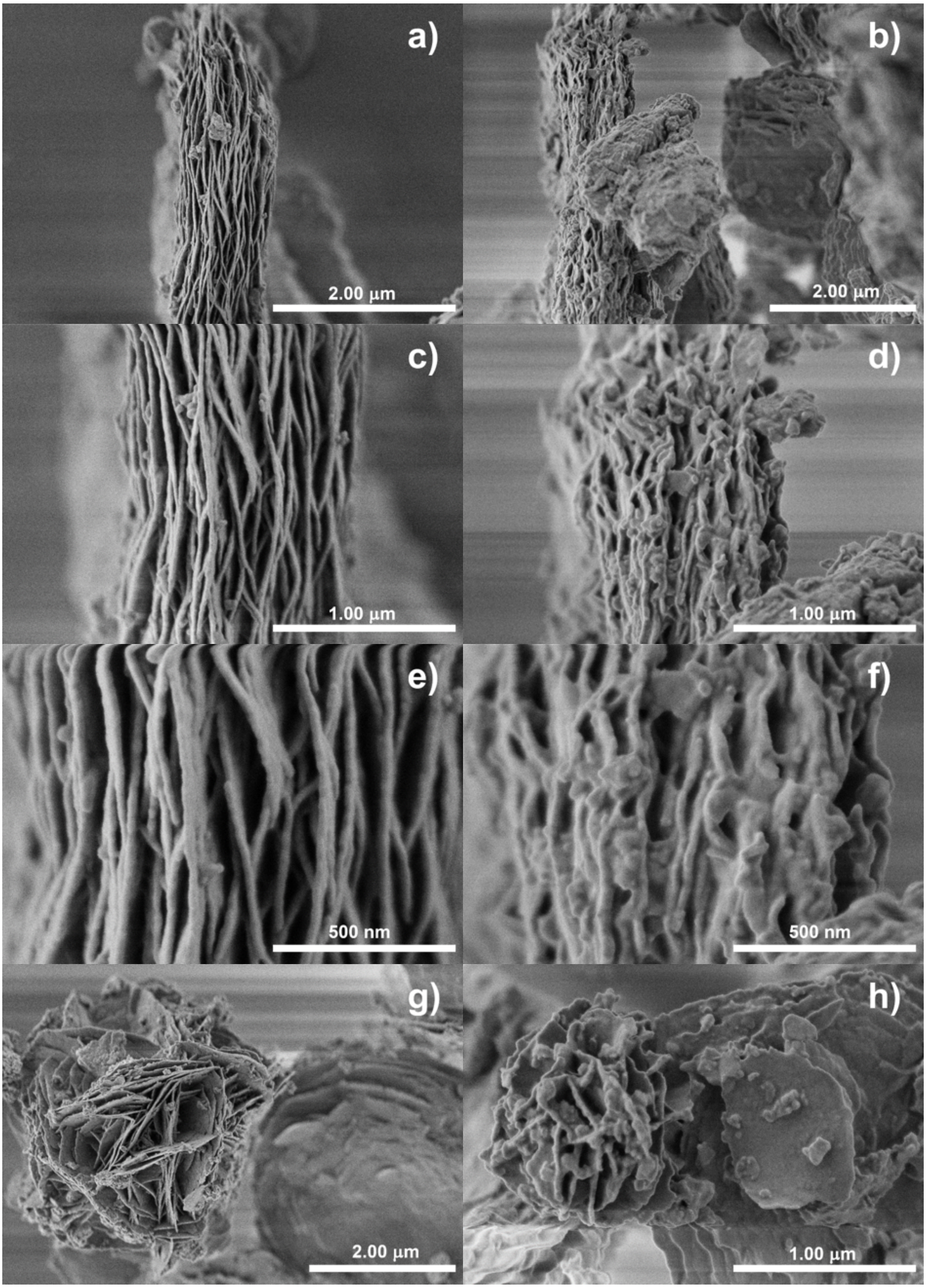

Figure S82. SEM images of $[Mg(pz)_2]_n$ (**8**, a), c), e), g)) and **8** after one week exposure to ambient atmosphere (b), d), f), h)).

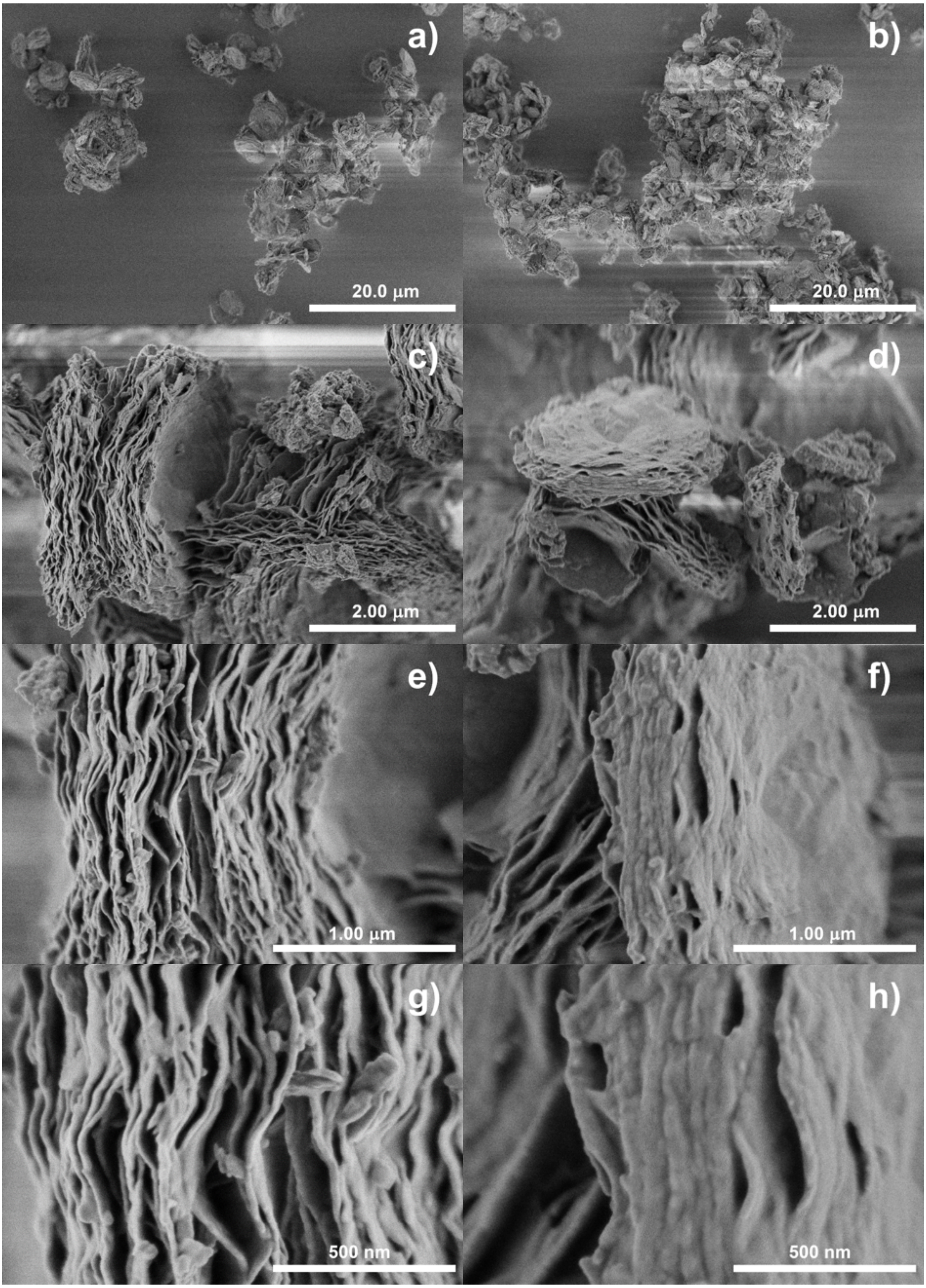

Figure S83. SEM images of $[Mg(pz)_2]_n$ (**8**, a), c), e), g)) and **8** after one week exposure to ambient atmosphere (b), d), f), h)).

Table S6. Crystallographic Data for **2**, **3-thf**, **5** and **4a-Hpz**

	**2**	**3-thf**	**5**	**4a-Hpz**
formula	$C_{48}H_{78}K_2Mg_2N_{12}$	$C_{40}H_{68}Mg_2N_8O_2$	$C_{52}H_{94}Mg_3N_8O_2$	$C_{72}H_{122}Mg_3N_{16}$
CCDC	2342200	2342196	2342195	2342199
M [g mol^{-1}]	950.04	741.64	936.28	1284.78
color/shape	colorless/prism	colorless/block	colorless/block	colorless/column
Crystal dimensions [mm]	0.137 x 0.114 x 0.076	0.251 x 0.204 x 0.101	0.249 x 0.129 x 0.101	0.437 x 0.184 x 0.139
cryst. system	triclinic	triclinic	monoclinic	monoclinic
space group	$P\bar{1}$	$P\bar{1}$	$P2/n$	$P2_1/c$
a [Å]	9.844(2)	10.6846(7)	16.1661(13)	21.9180(10)
b [Å]	11.547(3)	10.7816(7)	10.4304(8)	18.1705(8)
c [Å]	13.742(3)	11.3605(8)	18.4158(15)	20.3234(9)
[illegible] [°]	71.277(4)	66.078(2)	90	90
[illegible] [°]	74.381(4)	68.914(2)	112.411(2)	103.1070(10)
[illegible] [°]	66.942(4)	73.097(2)	90	90
V [Å^3]	1342.7(5)	1099.96(13)	2870.7	7883.2(6)
Z	1	1	2	4
T [K]	173(2)	100(2)	100(2)	100(2)
wavelength [Å]	0.71073	0.71073	0.71073	0.71073
ρ_{calcd} [g cm^{-3}]	1.175	1.120	1.083	1.083
μ [mm^{-1}]	0.243	0.096	0.096	0.087
F(000)	512	404	1028	2808
θ range [°]	1.586/28.734	2.041/28.723	1.430/26.375	1.472/28.334
unique reflns	6953	5697	10731	18652
observed reflns	53540	34062	5742	86840
R_1/wR_2 (I>2[illegible])[a]	0.0426/0.0990	0.0426/0.1102	0.0722/0.1555	0.0522/0.1170
R_1/wR_2 (all data)[a]	0.0617/0.1100	0.0530/0.1179	0.1148/0.1773	0.0913/0.1355
GOF[b]	1.037	1.031	1.036	1.017

[a] $R_1 = \Sigma(||F_0| - |F_c||) / \Sigma|F_0|$, $F_0 > 4\sigma(F_0)$, $wR_2 = \{\Sigma[w(F_0^2 - F_c^2)^2 / \Sigma[w(F_0^2)^2]\}^{1/2}$. [b] $GOF = [\Sigma w(F_0^2 - F_c^2)^2 / (n_0 - n_p)]^{1/2}$.

Table S7. Crystallographic Data for **4-thf**, **6-thf** and **$1\text{-}CO_2$,thf**, and **$LiMg_4(CO_2{\cdot}Pz^{iPr,iPr})_9$**

	**4-thf***	**6-thf**	**$1\text{-}CO_2$,thf**	**$LiMg_4(CO_2{\cdot}Pz^{iPr,iPr})_9$***
formula	$C_{62}H_{106}Mg_3N_{12}O_2$	$C_{32}H_{28}F_{24}Mg_2N_8O_3$	$C_{32}H_{54}MgN_4O_6$	2 $C_{90}H_{135}LiMg_4N_{18}O_{18}$, 5·$C_7H_8$
CCDC	2342201	2342198	2342197	2352312
M [g mol^{-1}]	1124.51	1077.24	615.10	4183.33
color/shape	colorless/block	yellow/column	colorless/column	colorless/plate
Crystal dimensions [mm]	0.088 x 0.038 x 0.035	0.391 x 0.143 x 0.120	0.303 x 0.138 x 0.105	0.148 x 0.118 x 0.045
cryst. system	monoclinic	monoclinic	monoclinic	orthorhombic
space group	*P*2$_1$/*c*	*C*2/*c*	*P*2$_1$/*n*	*Pbca*
a [Å]	32.802(3)	27.807(3)	8.6250(7)	21.2556(7)
b [Å]	10.5871(11)	9.5838(9)	10.9218(9)	22.0236(8)
c [Å]	19.763(2)	18.5235(17)	17.9314(14)	50.9504(16)
[illegible] [°]	90	90	90	90
[illegible] [°]	103.477(7)	123.1410(10)	93.313(2)	90
[illegible] [°]	90	90	90	90
V [Å^3]	6674.3(12)	4133.4(7)	1686.3(2)	23851.2(14)
Z	4	4	2	4
T [K]	100(2)	100(2)	100(2)	100
wavelength [Å]	1.54184	0.71073	0.71073	1.54178
ρ_{calcd} [g cm^{-3}]	1.119	1.731	1.211	1.165
μ [mm^{-1}]	0.789	0.213	0.100	0.832
F(000)	2456	2160	668	8968
θ range [°]	2.770/50.221	2.221/28.724	2.184/29.552	2.707/50.501
unique reflns	6222	5336	4700	12499
observed reflns	79269	32769	42441	94548
R_1/wR_2 (I>2[illegible])[a]	0.0888/0.1572	0.0372/0.0911	0.0375/0.0958	0.0848/ 0.2006
R_1/wR_2 (all data)[a]	0.2489/0.2221	0.0490/0.0998	0.0446/0.1021	0.1520/ 0.2395
GOF[a]	1.015	1.041	1.037	1.046

[a]$R_1 = \Sigma(||F_0| - |F_c||) / \Sigma|F_0|$, $F_0 > 4\sigma(F_0)$, $wR_2 = \{\Sigma[w(F_0^2 - F_c^2)^2 / \Sigma[w(F_0^2)^2]\}^{1/2}$. [b]$GOF = [\Sigma w(F_0^2 - F_c^2)^2 / (n_0 - n_p)]^{1/2}$.

* Connectivity structure only.

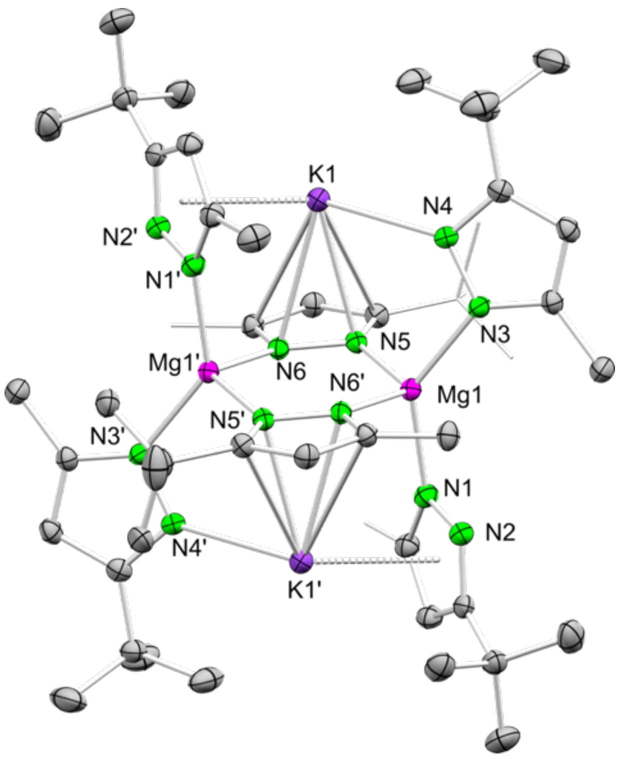

Figure S84. Crystal structure of $[Mg(pz^{tBu,Me})_2(Kpz^{tBu,Me})]_2$ (**2**). Ellipsoids are set at the 50% probability level. Hydrogen atoms are omitted and some Me/*t*Bu moieties are displayed as wireframe for clarity. Selected interatomic distances [Å]: Mg1–N1 2.0541(14), Mg1–N3 2.0414(14), Mg1–N5 2.0759(14), Mg1–N6' 2.1061(14), K1–N4 2.8419(14), K1–N5 2.7940(14), K1–N6 2.9040(14), K1–Ct 2.800, N(pz)–C(pz(3/5-position)) 1.340(2)-1.3567(19), C(pz(4-position))–C(pz(3/5-position)) 1.381(2)-1.406(2). Selected angles [°]: N3–Mg1–N1 135.24(6), N3–Mg1–N5 104.92(6), N1–Mg1–N5 99.90(6), N3–Mg1–N6' 103.18(6), N1–Mg1–N6' 99.59(5), N5–Mg1–N6' 114.27(5). '= -x+1, -y+1, -z+1

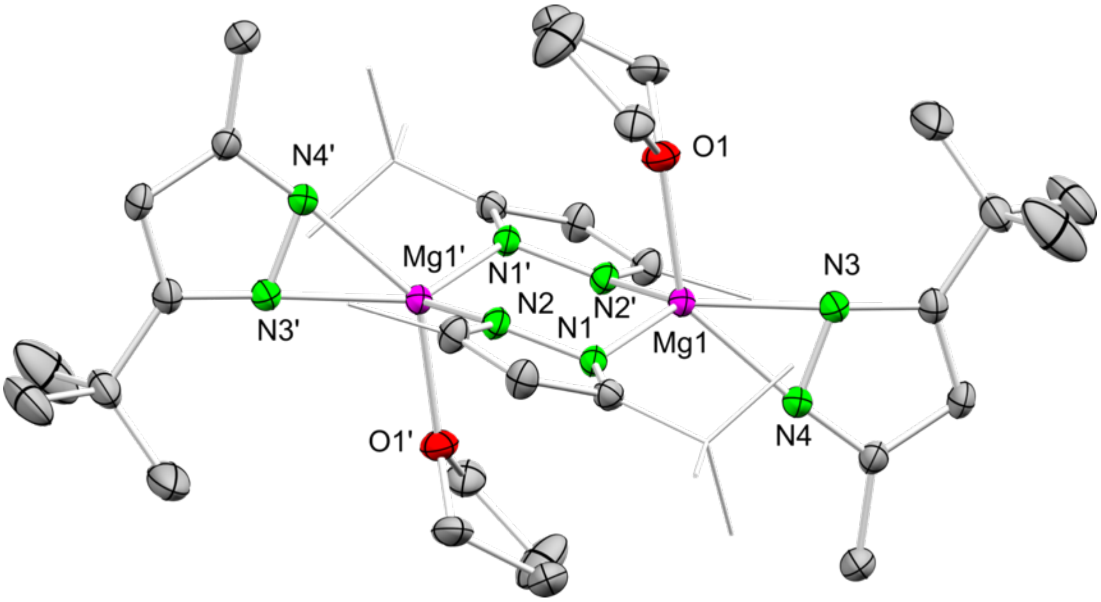

Figure S85. Crystal structure of [Mg(pz^{tBu,Me})$_2$(thf)]$_2$ (**3-thf**). Ellipsoids are set at the 50% probability level. Hydrogen atoms are omitted and some Me/*t*Bu moieties are displayed as wireframe for clarity. Selected interatomic distances [Å]: Mg1–N1 2.0783(10), Mg1–N2' 2.0890(11), Mg1–N3 2.0454(11), Mg1–N4 2.0965(11), N(pz)–C(pz(3/5-position)) 1.3377(15)-1.3530(15), C(pz(4-position))–C(pz(3/5-position)) 1.3898(17)-1.3970(17). Selected angles [°]: N3–Mg1–O1 101.22(4) N3–Mg1–N2' 114.10(4), O1–Mg1–N2' 93.98(4), N3–Mg1–N4 39.25(4), O1–Mg1–N4 140.35(4), N2'–Mg1–N4 104.89(4), O1–Mg1–N1 92.42(4), N1–Mg1–N2' 113.83(4), N1–Mg1–N4 110.40(4), N3– Mg1–N2' 114.10(4). '= -x+1, -y, -z+1

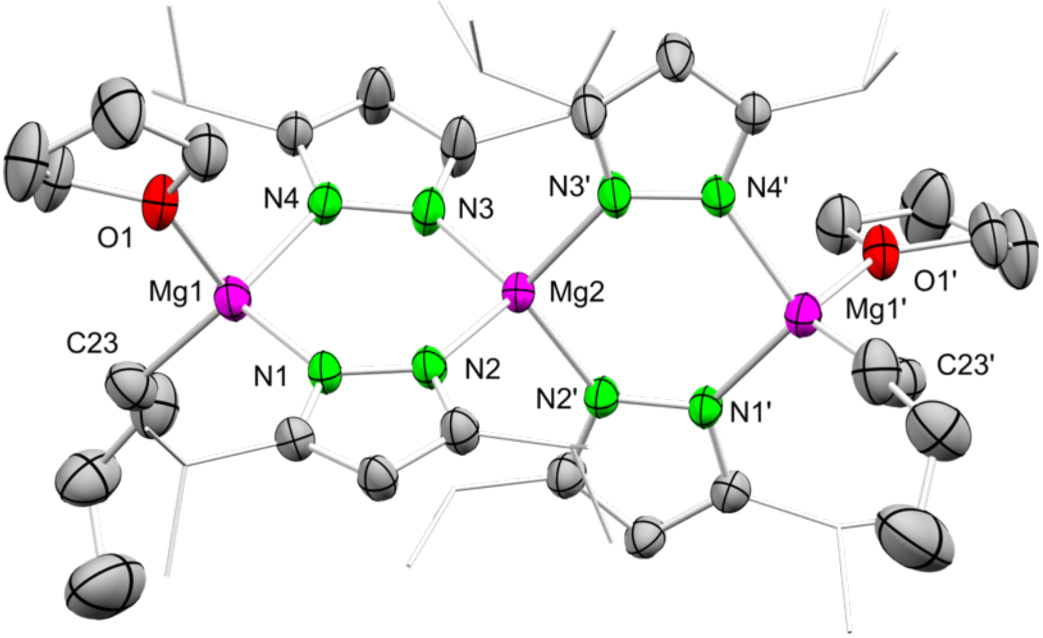

Figure S86. Crystal structure of [Mg$_3$(pz^{iPr,iPr})$_4$(*n*Bu)$_2$(thf)$_2$] (**5**). Ellipsoids are set at the 50% probability level. Hydrogen atoms are omitted and *i*Pr moieties are displayed as wireframe for clarity. Selected interatomic distances [Å]: Mg1–N1 2.081(3), Mg1–N4 2.071(3), Mg2–N2 2.059(3), Mg2–N3 2.055(3), Mg1–O1 2.051(3), Mg1–C23/C23A 2.140(13)/2.09(2)*, N(pz)–C(pz(3/5-position)) 1.338(5)-1.347(4), C(pz(4-position))–C(pz(3/5-position)) 1.376(5)-1.392(5). Selected angles [°]: O1–Mg1–N4 102.97(12), O1–Mg1–N1 99.40(12), N4–Mg1–N1 106.52(11), O1–Mg1–C23A 100.3(6), N4–Mg1–C23 121.0(7), N1–Mg1–C23 122.0(8), O1–Mg1–C23 113.0(3), N4–Mg1–C23 114.5(4), N1–Mg1–C23 118.4(4), N3'–Mg2–N3 108.63(19), N3'–Mg2–N2' 109.12(11), N3'–Mg2–N2 110.92(11), N3'–Mg2–N2 110.92(11), N3–Mg2–N2 109.12(11), N2'–Mg2–N2 108.15(18). '= -x+1/2, y, -z+3/2 *disorder

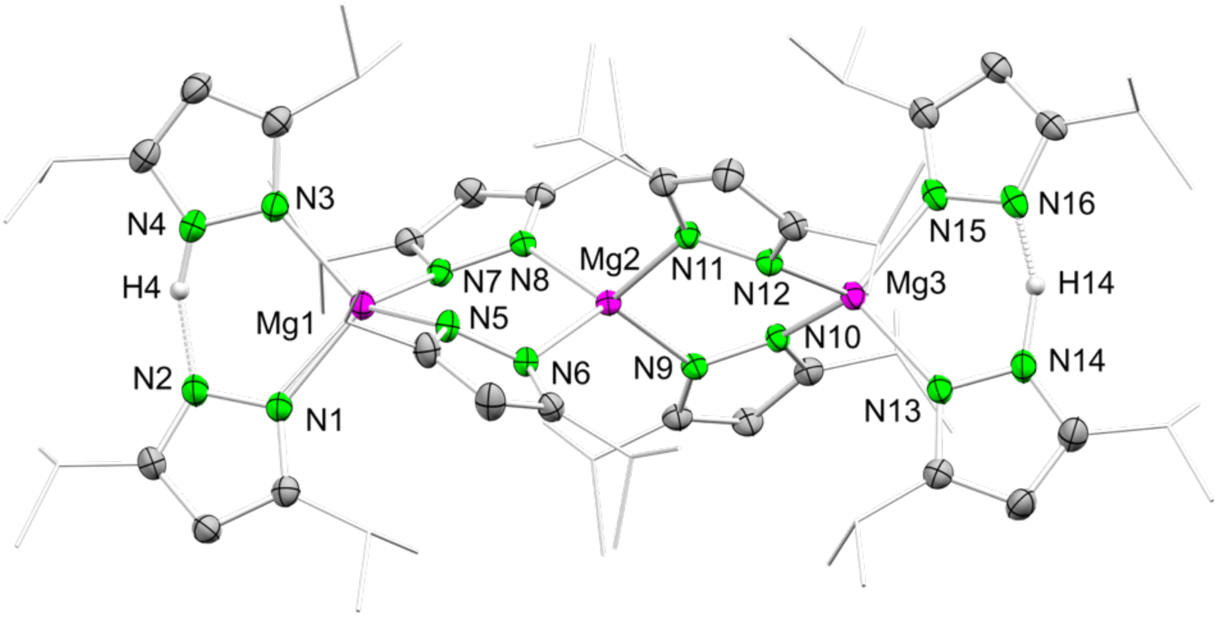

Figure S87. Crystal structure of [$Mg_3(pz^{iPr,iPr})_6(Hpz^{iPr,iPr})_2$] (**4a-Hpz**). Ellipsoids are set at the 50% probability level. Hydrogen atoms are omitted and *i*Pr moieties are displayed as wireframe for clarity. Selected interatomic distances [Å]: Mg1–N 2.0485(15)-2.1000(16), Mg2–N 2.0846(15)-2.1008(15), Mg3–N 2.0374(15)-2.0915(16), N2–H4A 1.61(3), N4–H4A 1.05(3), N14–H14 1.06(3), N16–H14 1.60(3). Selected angles [°]: N5–Mg1–N7 102.25(6), N5–Mg1–N1 109.91(6), N7–Mg1–N1 121.34(6), N5–Mg1–N3 118.44(6), N7–Mg1–N3 106.26(6), N1–Mg1–N3 99.66(6), N8–Mg2–N11 122.50(6), N8–Mg2–N6 101.06(6), N11–Mg2–N6 101.74(6), N8–Mg2–N9 105.56(6), N11–Mg2–N9 102.95(6), N6–Mg2–N9 124.94(6), N12–Mg3–N10 103.85(6), N12–Mg3–N15 108.94(6), N10–Mg3–N15 118.91(6), N12–Mg3–N13 114.92(6), N10–Mg3–N13 110.26(6), N15–Mg3–N13 100.48(6).

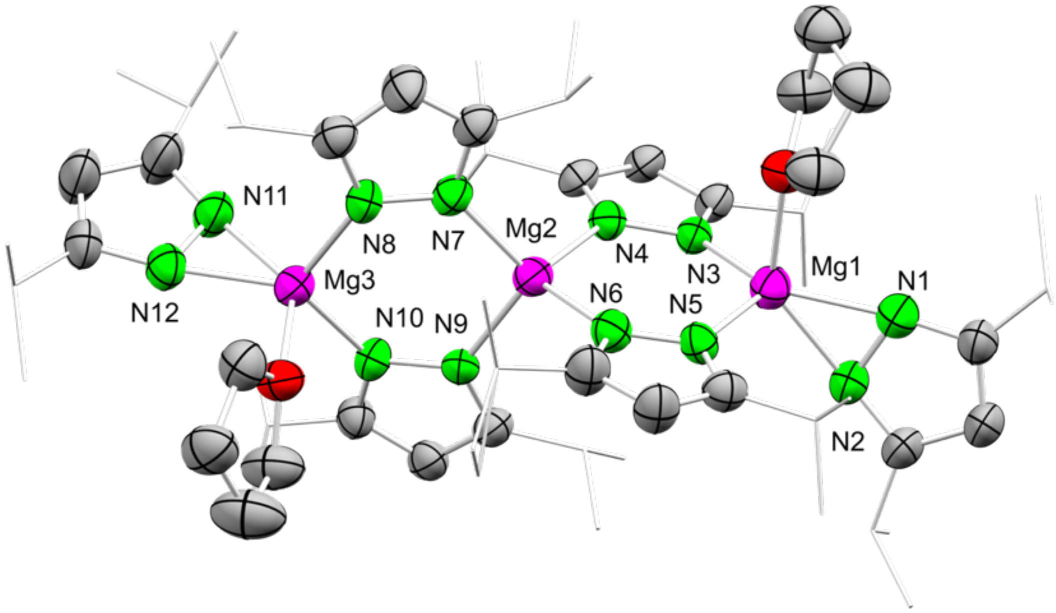

Figure S88. Crystal structure of [$Mg_3(pz^{iPr,iPr})_6(thf)_2$] (**4-thf**). Ellipsoids are set at the 50% probability level. Hydrogen atoms are omitted and *i*Pr moieties are displayed as wireframe for clarity (connectivity structure only).

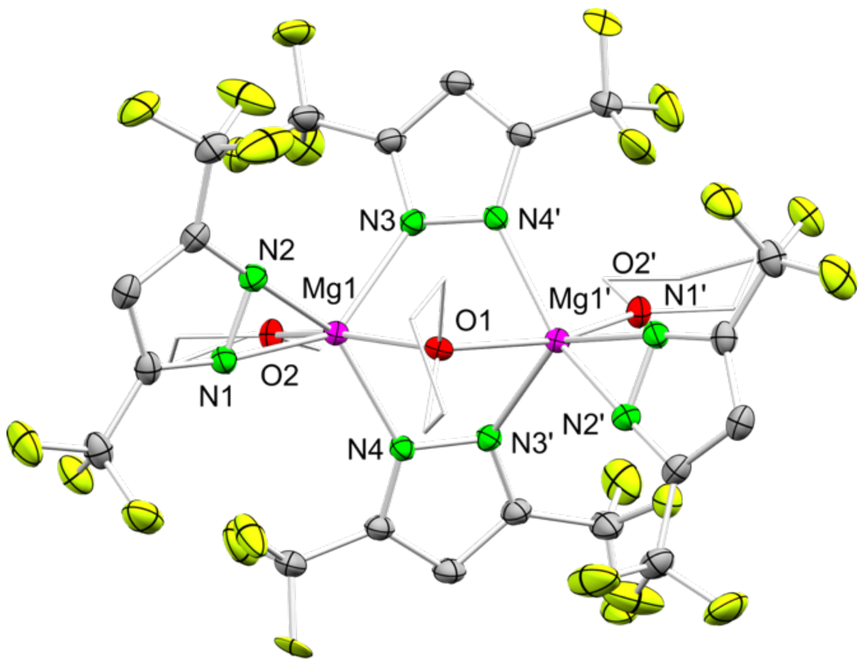

Figure S89. Crystal structure of $[Mg_2(pz^{CF_3,CF_3})_4(thf)_3]$ (**6-thf**) Ellipsoids are set at the 50% probability level. Hydrogen atoms are omitted and THF donors are displayed as wireframe for clarity. Selected interatomic distances [Å]: Mg1–N1 2.1533(13), Mg1–N2 2.0939(13), Mg1–N3 2.1577(12), Mg1–N4 2.1386(12), Mg1–O1 2.2979(10), Mg1–O2 2.0554(11). Selected angles [°]: O2–Mg1–N2 107.94(5), O2–Mg1–N4 88.27(4), N2–Mg1–N4 145.30(5), O2–Mg1–N1 95.86(5), N2–Mg1–N1 37.44(5), N4–Mg1–N1 112.47(5), O2–Mg1–N3 92.92(5), N2–Mg1–N3 101.02(5), N4–Mg1–N3 108.74(5), N1–Mg1–N3 138.02(5), O2–Mg1–O1 163.32(4), N2–Mg1–O1 88.73(4), N4–Mg1–O1 77.58(4), N1–Mg1–O1 97.68(4), N3–Mg1–O1 83.47(4).

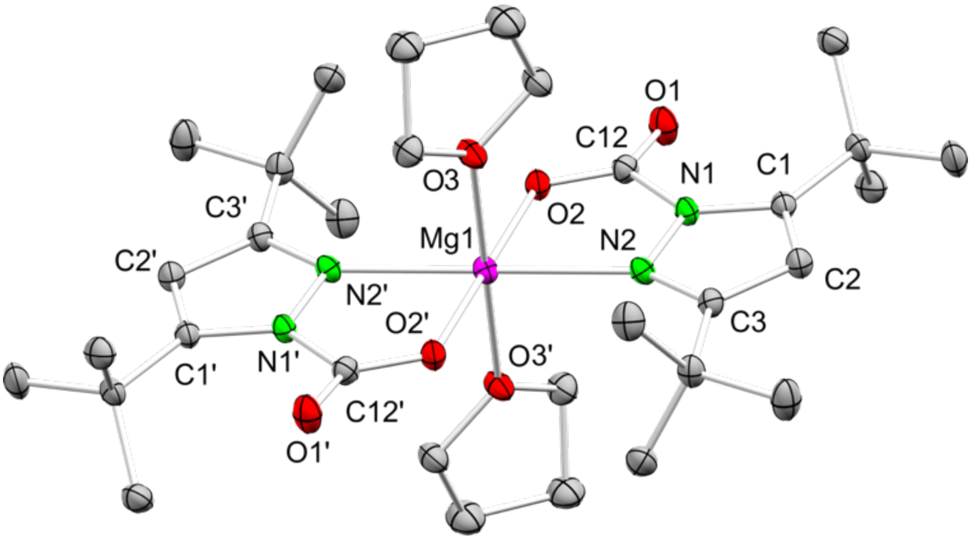

Figure S90. Crystal structure of $[Mg(CO_2{\cdot}pz^{tBu,tBu})_2(thf)_2]$ (**1-CO₂,thf**). Ellipsoids are set at the 50% probability level. Hydrogen atoms as well as disorder in the thf ligands are omitted for clarity. Selected interatomic distances [Å]: Mg1–O2 1.9917(7), Mg1–N2 2.2273(8), Mg1–O3 2.1095(7), C12–O1 1.2165(12), C12–O2 1.2621(11), C12–N1 1.4773(12), C2–C1 1.3788(13), C2–C3 1.4073(13), N1–C1 1.3800(12), N2–C3 1.3310(12). Selected angles [°]: O1–C12–O2 129.23(9), O2–Mg1–N2 75.33(3), O2–Mg1–O3 90.95(3), O2–Mg1–N2 75.33(3), O2'–Mg1–N2 104.67(3), O2–Mg1–O2' 180.0. '= -x+1, -y+1, -z+1

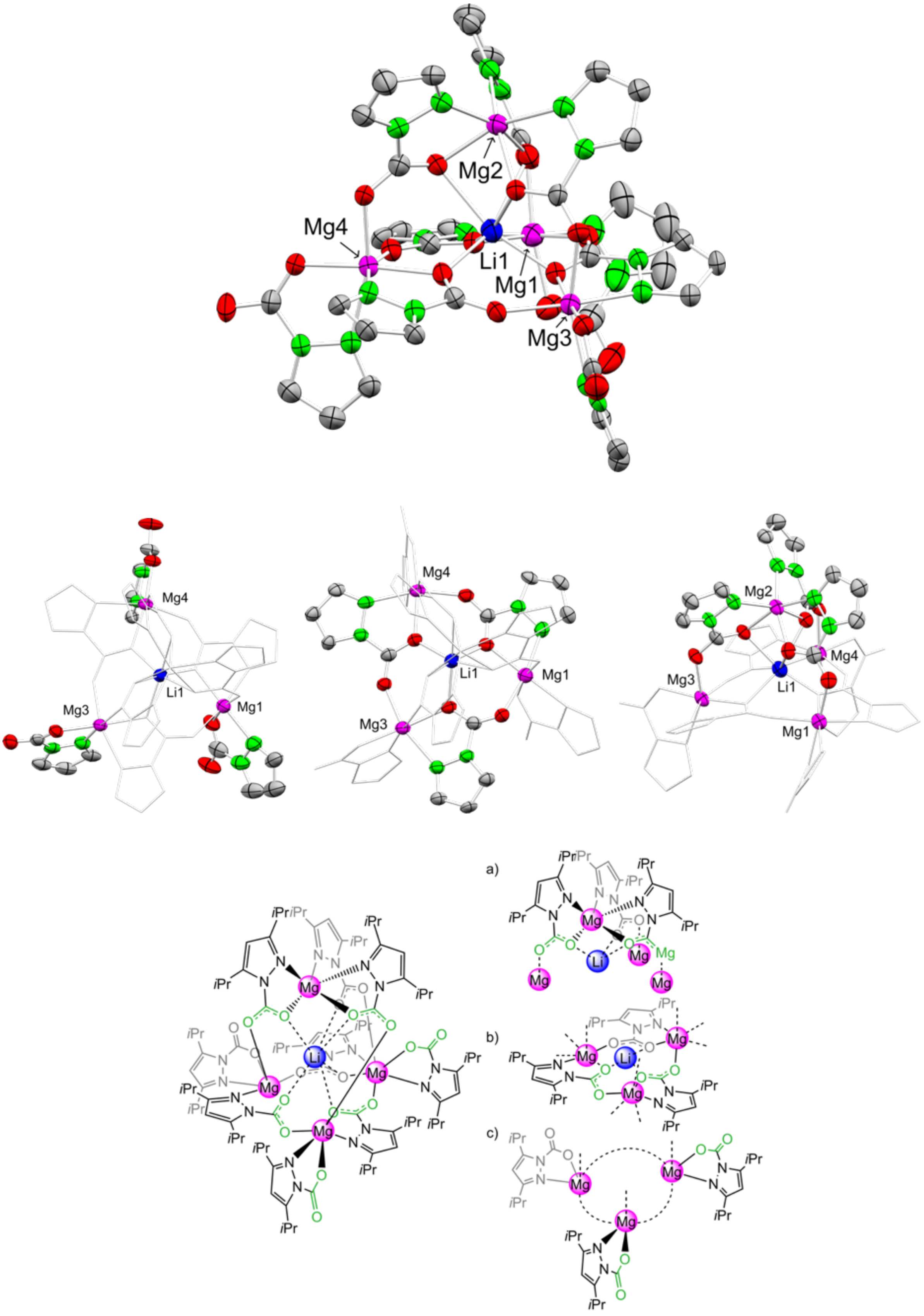

Figure S91. Crystal structure of $[LiMg_4(CO_2{\cdot}pz^{iPr,iPr})_9]$ (connectivity). Ellipsoids are set at the 50% probability level. Hydrogen atoms as well as lattice solvent toluene and THF are omitted for clarity. For a better description of the molcular structure, sections of the distinct metal-carbamato coordination are highlighted and further clarified by ChemDraw representations.

[1] G. Yang, R. G. Raptis, *Inorg. Chim. Acta* **2003**, *352*, 98–104.
[2] *COSMO V. 1.61*, Bruker AXS Inc., Madison, WI, **2012**.
[3] *APEX3 V. 2019.11-0*, Bruker AXS Inc., Madison, WI, **2019**.
[4] *SAINT V. 8.38A*, Bruker AXS Inc., Madison, WI, **2017**.
[5] SADABS L. Krause, R. Herbst-Irmer, G. M. Sheldrick D. Stalke, *J. Appl. Cryst.* **2015**, *48*, 3–10.
[6] a: G. M. Sheldrick, *Acta Cryst. A* **2015**, *71*, 3–8. b: G. M. Sheldrick, *Acta Cryst. C* **2015**, *71*, 3–8.
[7] SHELXLE, C. B. Hübschle, G. M. Sheldrick, B. Dittrich, *J. Appl. Cryst.* **2011**. *44*, 1284–1284
[8] D. Kratzert, J. J. Holstein, I. Krossing, DSR: enhanced modelling and refinement of disordered structures with SHELXL. *J. Appl. Cryst.* **2015**, *48*, 933–938.
[9] C. F. Macrae, I. J. Bruno, J. A. Chisholm, P. R. Edgington, P. McCabe, E. Pidcock, L. Rodriguez-Monge, R. Taylor, J. Van de Streek, P. A. Wood, *J. Appl. Cryst.* **2008**, *41*, 466–470.
[10] D. Pfeiffer, M. J. Heeg, C. H. Winter, *Angew. Chem. Int. Ed.* **1998**, *37*, 2517–2519.
[11] R. Neufeld, D. Stalke, *Chem. Sci.* **2015**, *6*, 3354–3364.
[12] N. Masciocchi, S. Galli, E. Alberti, A. Sironi, C. Di Nicola, C. Pettinari, L. Pandolfo, *Inorg. Chem.* **2006**, *45*, 9064–9074.
